# Move-PCD—a multi-center longitudinal randomized controlled superiority trial on the effect of a 6-month individualized supported physical activity (PA) program on quality of life (QoL) in children, adolescents, and adults with primary ciliary dyskinesia

**DOI:** 10.1186/s13063-024-08379-0

**Published:** 2024-08-15

**Authors:** Anna Teresa Hoffmann, Anna Mai, Klaus Baum, Anne Schlegtendal, Christoph Maier, Julien Stein, Marianne Tokic, Stefanie Dillenhöfer, Thomas Lücke, Nina Timmesfeld, Folke Brinkmann

**Affiliations:** 1 University Clinic for Pediatrics and Adolescent Medicine, Ruhr University Bochum, Alexandrinenstr. 5, 44791 Bochum, Germany; 2 Medical Informatics, Biometry and Epidemiology (AMIB), Ruhr University Bochum, 44780 Bochum, Germany; 3 Section for Pediatric Pneumology and Allergology, University Clinic Schleswig Holstein, Campus Lübeck, Ratzeburger Allee 160, 23538 Lübeck, Germany; 4 Trainingsinstitut Prof. Dr. Baum, Wilhelm-Schlombs-Allee 1, 50858 Cologne, Germany

**Keywords:** PCD, Standard therapy, Physical fitness, Motor skills, Physical activity program

## Abstract

**Background:**

Primary ciliary dyskinesia (PCD) is a rare genetical disease with malfunction of the motile cilia leading to impaired muco-ciliary clearance in the respiratory tract. There is no cure for PCD, only supportive therapy aimed at minimizing the progression of the disease and improving the patient’s quality of life (QoL). Physical activity (PA) is one of these recommended supportive therapies for people with PCD (pwPCD). However, there is no scientific evidence to support this recommendation. In addition, regular medical advice to increase PA remains largely ineffective in pwPCD.

**Methods:**

To test the main hypothesis, that an individualized and supported PA program leads to a better QoL 6 months after randomization (QoL-PCD questionnaire) compared to usual recommendation in pwPCD, 158 pwPCD aged 7 to 55 years are to be included in this multi-center randomized controlled trial (RCT). After the screening visit, a 1:1 randomization stratified by age group and FEV1 will be performed. A QoL-PCD questionnaire, motor test, and lung function will be carried out at regular intervals in both groups. PA is recorded in both groups using activity trackers during the study period. The main aim of the trial is to estimate the difference in the change of QoL between the groups after 6 months. Therefore, our full analysis set consists of all randomized patients and analysis is performed using the intention-to-treat principle. Statistical software R (http://www.r-project.org) is used. Ethical approvement without any reservations: RUB Bochum Ethics Committee (No. 23–7938; December 4, 2023). Recruitment start: March 2024.

**Discussion:**

Limitations result from the rarity of PCD with its broad disease spectrum and the large age range. These are reduced by stratified randomization and the measurement of the individual change in QoL as primary endpoint. In our view, only a PA program tailored to individual needs with close contact to trainers offers the chance to meet personal needs of pwPCD and to establish PA as a pillar of therapy in the long term. The study protocol explains all procedures and methods of recruitment, implementation of the study visits and intervention, measures for patient and data safety, and for minimizing risks and bias.

**Trial registration:**

German Clinical Trials Register (DRKS) 00033030. Registered on December 7, 2023. Update 10 July 2024.

**Study protocol version 10:**

Version 1.2; 12 June 2024

**Supplementary Information:**

The online version contains supplementary material available at 10.1186/s13063-024-08379-0.

## Introduction

### Background and rationale

Primary ciliary dyskinesia (PCD) is a rare genetical disease characterized by malfunction of the motile cilia leading to impaired muco-ciliary clearance in the respiratory tract. More than 50 different gene mutations causing different ciliary defects are already known, which explains the heterogeneity of the disease. A chronic inflammation leads to loss of lung function and irreversible lung damage. There is no cure for primary ciliary dyskinesia, only supportive therapy, which aims to mitigate the progression of the disease as much as possible and improves the patient’s quality of life (QoL). Many of the therapy recommendations originate from the therapy of people with cystic fibrosis (pwCF). Physical activity (PA) is also recommended as a therapy for people with PCD (pwPCD). Along with respiratory physiotherapy, it is already one of the essential pillars of therapy [1–6]. However, regular recommendations by the physician to increase physical activity remain mostly ineffective.

As far as we know, no studies exist on long-term impact of physical activity on QoL, lung function, and survival for pwPCD [3, 7]. This randomized, multi-center control trial will be the first to determine the effects of improving motivation to exercise, i.e., of individualized and supervised PA over 6 months in pwPCD on QoL and clinical outcome. Based on clinical practice and experience from studies with pwCF [8–10], we assume a significant improvement in QoL as well as a slowing of disease progression through regular and, above all, mentored PA. This study could thus close the evidence gap for PA as part of PCD therapy and lead to improved adherence to PA in pwPCD.

Physical activity and aerobic fitness of pwPCD is significantly lower than in healthy individuals [11, 12]. In children with PCD, cardiopulmonary fitness is equally and consistently reduced as in pwCF [5]. In addition, lower physical performance is known to be associated with reduced lung function [2].

As we know from studies in pwCF, external motivation is essential for adherence to exercise [13]. The international study ACTIVATE CF about PA in pwCF concluded that pwCF need to be motivated to be physically active without pressure but with adequate support to build positive long-term activity behavior [9]. The CFmobil study demonstrated the benefits of an individually adapted and supervised long-term physical activity intervention for motor performance in children and adolescents with CF. It could be shown that motor skills improved during the 6-month intervention phase of the study but stagnated afterwards [13–15]. The results suggest that an individually adapted, guided, and regularly performed exercise program should include all aspects of physical fitness [13, 15]. In addition, health-related QoL in pwCF is linked to physical fitness [8–10, 16–18].

Sonbahar-Ulu et al. were the first to analyze the influence of PA in pwPCD. They demonstrated a positive effect of PA for 6–18-year-olds on lung function and QoL using active video gaming over 8 weeks [19, 20], but there are no studies on long-term effects of physical activity on QoL, lung function, and survival for pwPCD [3, 7]. According to the therapy guidelines, PA is recommended for pwPCD, but without scientific evidence.

We searched for evidence using the electronic databases for the keywords “primary ciliary dyskinesia,” “cystic fibrosis,” “bronchiectasis,” “physical activity,” and “quality of life.”

### Objectives

Against this background, the aim of this study is to evaluate the efficacy of PA in pwPCD. Based on clinical practice and experience from studies with pwCF [8–10], we assume a significant improvement in QoL as well as a slowing of disease progression through regular and, above all, mentored PA. This study shall close the evidence gap for PA as part of PCD therapy and lead to improved adherence to PA in pwPCD.

The main hypothesis of the trial is as follows: The support from an individualized and supported PA program results in higher PA and hence in a better quality of life 6 months after randomization (measured by physical functioning domain of QoL-PCD questionnaire) compared to treatment as usual in pwPCD with sole recommendation for PA (Table 1).

**Table 1:** Objectives and endpoints

Objectives	Endpoints	Justification for endpoints
Primary		
To improve quality of life after 6 months of individual physical activity	Quality of life (physical functioning domain of the QoL-PCD questionnaire)	Well-established patient-relevant outcome
Secondary		
To evaluate the effect of the intervention on lung health, physical activity and fitness, as well as safety	Lung function parameters (VCmax (maximum vital capacity), FVC (forced vital capacity), FEV1 (forced expiratory volume in 1 s), PEF (maximum expiratory flow or peak flow), MEF 75/25 (maximum expiratory flow at 75/25% of FVC), sReff (specific airway resistance, if available), TLC (total lung capacity, if available)), Lung Clearance Index (LCI), if available	Relevant surrogates for health status and physical fitness of pwPCD with a chronic lung disease
	Physical fitness (motor test)	
	Physical activity (step count via activity tracker Garmin Vivo fit 4®)	Detection of the mechanism of action
	Quality of life (overall and single domains of the QoL-PCD questionnaire)	Detailed analysis of a well-established patient-relevant outcome
	Adverse events	Safety of intervention must be established

### Study design

The main hypothesis will be tested in a multi-center, parallel group, randomized controlled superiority trial (RCT). Randomization 1 will be done on patient-level. This RCT is used for investigation of the efficacy of PA in pwPCD.

## Methods

### Study population

The aim is to recruit a population of people with PCD in seven study centers in Germany, all of which are experienced in the care of pwPCD, and with the support of the patient organization “Kartagener Syndrom und Primäre Ciliäre Dyskinesie e.V.” (e.g., from the 2024 PCD Patient Congress).

The participants in the study should be able to perform a PA program safely, to perform the motor tests reliably, and to have a certain level of media literacy and a sufficient attention span to complete the online PA courses. The study centers require a PCD outpatient clinic with physicians experienced in care, the possibility of ECG, and pulmonary function diagnostics.

#### Inclusion criteria

To be eligible to participate in this study, an individual has to meet all the following criteria:

- Children and adolescents ≥ 7 years and adults ≤ 55 years old
- Confirmed diagnosis of primary ciliary dyskinesia according to ERS criteria [21] (PCD confirmed or highly likely)
- Signed and dated informed consent form of the participants and of the guardian (in case of minors)
- Willingness to participate in the activity program and weekly phone contacts, and to wear the activity tracker for 1 year

#### Exclusion criteria

Meeting one of the following criteria leads to an exclusion of this study:

- Disability to take part in a PA program due to medical or other conditions [22–28]
  - Decompensated heart failure
  - Cor pulmonale
  - Hypertrophic cardiomyopathy
  - Arterial hypertension that is not normal under medical therapy
  - Myocarditis in the last 6 months
  - Unstable/progressive angina pectoris (e.g., new onset of angina, increase in symptoms, more need for medication)
  - Uncontrolled arrhythmias (e.g., recurrent ventricular tachycardia, tachyarrhythmias, atrial fibrillation)
  - Medium- to high-grade cardiac vitals, especially of the left heart (high-grade or symptomatic aortic valve stenosis, high-grade mitral valve insufficiency)
  - Heart attack in the last 3 months incl. PTCA (percutaneous coronary intervention)
  - Heart operations (incl. pacemaker/ICD operation in the last 3 months)
  - Apoplexy in the last 5 years
  - Marfan/Ehlers-Danlos syndrome
  - Uncontrolled bronchial asthma (i.e., on-demand medication more than 2 days a week or nocturnal symptoms)
  - Lung transplantation (also planned)
  - Respiratory partial insufficiency with intermittent or continuous oxygen therapy
  - Continuous NIV (non-invasive ventilation)
  - Hemato-oncological diseases currently under therapy
  - Physical inability to participate in PA or orthopedic limitations that preclude participation in the motor test battery or inclination to fall
  - Any condition, fact, or comorbidity for which, in the opinion of the study investigator, study participation is not possible to participate, which represent an additional risk for study participation or lead to a falsification of the result
  - Alcohol or drug abuse
  - Pregnancy (during the study drop-out criterion)
  - Physical complaints on exertion
    - Dyspnea beyond the expected level
    - Syncope
    - Chest pain/nausea
    - Tendency to fall (including musculoskeletal)
- Examination results leading to exclusion (see “Screen failures”)
  - Missing foot pulses
  - Central cyanosis
  - ST elevation, AV block of grade II or higher, Long-QT
  - FEV1 < 30%
  - sO2 < 90%
- Participation in another intervention trial

#### Screen failures

Screen failures are defined as participants who consent to participate in this study but are not subsequently randomized. Minimal information on demography, screening failure details, and eligibility criteria will be given on the screening log. Screen failures due to medical contraindications may receive a recommendation for further medical clarification and counseling.

Individuals who do not meet the criteria for participation in this study (screening failure), but who are suspected during screening to be more eligible after some time, e.g., because expected therapy effects will occur or an acute infection will have been cured, can be rescreened after 4 weeks and will be classified as “re-screening required before randomization.” Examples include termination of participation in another clinical trial or clinical examination results, such as exacerbations score according to Lucas ≥ 3 [29]. Rescreened participants will be assigned the same participant number as for the initial screening.

### Strategies for recruitment and retention

In the 7 study centers, 280 pwPCD are regularly supervised annually. According to these study centers, 193 of these pwPCD meet the inclusion criteria, so that recruitment of 100 participants is realistically feasible.

Patients will be informed by flyers and posters in their outpatient department. In addition, patients will be recruited through the patient organization “Kartagener Syndrom und Primäre Ciliäre Dyskinesie e.V.” (200 association members with PCD) and the annual patient congress (on average 40–50 participants with PCD in all age groups).

Recruitment procedures will differ between hospitals and the congress setting. Recruitment should fit as well as possible into the respective clinic’s daily routine and the congress program to ensure feasibility and success. The procedure described below will therefore be adapted to the conditions in the respective setting; screening manuals will be written. To allow an analysis of the feasibility of recruitment, numbers (*N*) of patients screened in the different steps shall be documented in a screening log.

#### General procedure in the hospital

In the seven study centers, eligible patients will be informed about the study by a study physician of the center during a regular follow-up visit. If this discussion (pre-screening) does not reveal any further exclusion criteria, the patient can be included in the study. After verbal and written information, written consent for study participation is obtained (in the case of minors, by the legal guardian) at pre-screening or at least at the beginning of the screening visit. If exclusion criteria arise during the screening visit, the patient will be classified as “non-participating” and will not be randomized. The screening visit data will be stored in the central study database and analyzed if the participant does not actively object to this. Re-screening is possible in case of remediable findings, for details see “Screen failures.” Participants for whom all inclusion criteria and no exclusion criteria apply are classified as “eligible to participate” and are randomized at the earliest 2 weeks after the motor test as part of the baseline examinations.

The dates for the following study visits are assigned by the study centers in coordination with the dates for the motor test by the training institute Prof. Dr. Baum. Whenever possible, the study visits should be combined with regular outpatient appointments.

#### General procedure for recruitment via the patient organization

- Recruitment as part of the patient congress

With the registration confirmation for the 2024 patient congress in Hamburg, the patient organization will send a reminder for the study. The inclusion and exclusion criteria are again pointed out here and congress participants are asked to bring all medical documents including cilia and genetic findings to the congress, if interested in study participation. This is necessary to be able to decide about eligibility for study participation at the congress. Congress participants who could take part in the study according to the inclusion and exclusion criteria will be informed verbally and in writing. After written information has been provided (see above), the screening visit including the motor test will be carried out on the same or following day. For the follow-up visits, appointments are either made at the lead study center in Bochum or the participants agree to a telephone visit. In this case, an examination close to home, including pulmonary function tests, is required by the treating doctor, for whom a confidentiality agreement must be in place. The following motor tests are carried out close to home after an appointment has been made with the training institute. The rest of the procedure corresponds to that in the study centers (see above).

b) Recruitment via mailing list or homepage

If a pwPCD contacts the central study center Bochum via advertising on the homepage or via the email distribution list of the patient organization, they will receive telephone and written information about the study. The inclusion and exclusion criteria will be checked in a telephone conversation. If participation in the study is not excluded in principle, an appointment will be made in Bochum for the screening visit and the motor test. Both will take part as described above for congress participants.

#### Procedures to enhance participant retention and motivation

All visit data are entered into the central database. The participants receive an automatic reminder email 1 week before the appointment. To improve adherence, all participants will also receive an automatic email once a month thanking them for taking part in the study (e.g., “It’s nice that you are part of the MOVE-PCD study”) and reminding them to wear the activity tracker. For added motivation, the online PA courses can continue to be taken at any time after receiving the release link, even beyond the intervention period. This also applies after the V4 has been reached for the control group.

### Study intervention

The intervention group receives an individualized PA program for 6 months considering personal preferences as well as the current state of fitness. The individual training plans are compiled by sports scientists with long-term experience in exercising with chronically ill people [30, 31]. The participant has the option of expanding his or her PA program at any time and is provided with additional training content for this purpose. To keep the entry threshold for training as low as possible, no minimum duration or frequency per week is specified. Nor are there any specifications regarding the weighting of endurance, strength, coordination, or mobility; instead, the patient’s wishes are considered. For standardization, the intervention consists of weekly online or telephone consultations with a personal coach. Possible topics to be discussed are implementation of the individual training plan, motivation, adaptation of the training as well as barriers to physical activities and their management.

In addition to the individual training plan, each week three different online courses are offered (1. Enjoy the day: mixture of flexibility, strengthening, and relaxation elements. 2. Strengthen the body: focus on strength build-up. 3. Move the rhythm: movement choreography). The advantage of the online PA courses, apart from hygienic aspects (no transfer of resistant pathogens), is that they can be implemented flexibly in terms of time and allow participants to enter the study at any time, even though the courses build on each other.

The intervention is based on the theory that personalized, regular, and individualized care in pwPCD will significantly increase and improve PA, thereby increasing treatment adherence to the extent that it has relevant effects on QoL and disease progression.

Standard therapy with inhalations of high-percentage saline and antibiotics, respiratory physiotherapy, nasal rinses, and oral intake of medications such as antibiotics and proton pump inhibitors are allowed. The standard therapy should not change relevantly during the study period.

If a medical condition during the study is decreased leading to inability to take part in PA, the patient has to stop the intervention but will be encouraged to complete at least the assessment of the primary efficacy endpoint.

#### Scientific rationale and justification for study design

To receive a high level of evidence, a randomized controlled trial is necessary. The study intervention is compared to the current standard of care [2]. Participants allocated to the control group receive treatment as usual with the mere recommendation to be physically active. Only by comparing standard therapy with individual PA in pwPCD is it possible to evaluate the effect on quality of life in PCD.

The duration of the intervention results from the fact that the physical activity of chronically ill people must be increased gradually and that the positive effects only appear after weeks of regular activity. However, this can be interrupted again and again in people by a worsening of the underlying disease, among other things. Participants may pause for a few days or weeks and then resume activity at a reduced level. Measuring the success of the intervention after a few weeks would therefore not be meaningful.

An evaluation of the success of the intervention after 6 months allows the assessment of the patients’ longer-term motivation for PA, and the ability of the qualified trainers to maintain this motivation, reduce barriers, strengthen resources, and thus initiate a sustainable behavior change in pwPCD.

We deliberately refrained from offering the control group the same number of personal contacts in order not to distort the intervention effect. Our study tests the effectiveness of a complex PA intervention in a real world setting against the current standard of care.

To also offer the control group a benefit of the study without confounding the outcome, all participants in the control group will receive access to the online PA courses after their last study visit (V4). It is up to the participants themselves whether and to what extent they use the offer.

#### Fidelity—interventionist training and tracking

The training institute Prof. Dr. Baum, consisting of sports scientists, sports physiotherapists, and physiologists experienced in training and research with patients with chronic lung diseases such as COPD [30, 31], develops individual training plans based on the results of the baseline motor tests and questionnaires. The assessors will all be trained for a standardized performance of assessments and tests. The team around Prof. Dr. Klaus Baum also carries out the weekly contacts to review and, if necessary, adjust the training plans and determine the safety of the intervention. The conversations are documented in the central database and will be standardized. Prior to the start of the study, Prof. Dr. Baum will instruct all employees on the special features of the disease PCD. During the intervention phase, a weekly online conference of all personal coaches is planned to exchange experiences. Critical cases or difficulties with coaching are discussed immediately with Prof. Dr. Baum.

#### Study intervention adherence

A large part of the intervention consists of weekly appointments with personal coaches. These also serve to document and check the adherence of the participants.

#### Study intervention discontinuation and participant discontinuation/withdrawal

The occurrence of diagnoses or examination results listed under “Exclusion criteria” or a positive answer in the weekly safety query described under “Safety assessments” will lead to an immediate discontinuation of the intervention.

When a subject discontinues from the PA program but not from the study, remaining study procedures will be completed as indicated by the study protocol. In individual cases, a decision may be made to continue the study without participation in the assessment of physical fitness.

In addition, all subjects are explicitly informed that if they have any doubts about PA and exercising, they should discontinue and contact the supervising or leading study center. Participants will be informed that if one of the following symptoms occurs, PA must be interrupted, or no further PA should be done until medical clarification is obtained:

- Fever or other infections accompanied by a feeling of illness
- Acute pulmonary exacerbation
- Severe joint, back, or head pain during or immediately after exercise
- Chest pain or left arm pain
- Dizziness/syncope
- Shortness of breath (beyond expected level) or hyperventilation
- Hypoglycemia
- Persistent palpitations or heart palpitations after exercise
- Pacemaker/ICD depending on type of PA

Participants are free to withdraw from participation in the study at any time upon request without giving reasons and without suffering any disadvantage, as stated to them prior to informed consent. Participants will be given the choice as to whether their withdrawal will only be from further contact from the trial team or withdrawal of all their unanalyzed data.

An investigator may discontinue a participant from the study for the following reasons:

- Lost-to-follow up; unable to contact subject (see below)
- Any event or medical condition or situation occurs such that continued collection of follow-up study data would not be in the best interest of the participant or might not be possible, e.g., longer inpatient stay

The reason for participant discontinuation or withdrawal from the study will be recorded on the study discontinuation form. Subjects who sign the informed consent form and are randomized but do not receive the study intervention may be replaced.

A participant will be considered lost to follow-up if he or she fails to return for one scheduled assessment and study staff are unable to contact the participant after five attempts.

The following actions must be taken if a participant does not return the required assessment:

- The site will attempt to contact the participant, within 1 week, counsel the participant on the importance of maintaining the assigned assessments and, if possible, will perform the assessment by telephone. Furthermore, the site will ascertain if the participant wishes to continue in the study.
- Before a participant is deemed lost to follow-up, the investigator or designee will make every effort to regain contact with the participant (where possible, 5 telephone calls, or 3 calls and two times a certified letter to the participant’s last known mailing address). These contact attempts will be documented in the participant’s medical record or study file.
- Should the participant continue to be unreachable, he or she will be considered to have withdrawn from the study with a primary reason of lost to follow-up.

If, for medical reasons (e.g., pulmonary exacerbation), one of the study visits cannot take place on the scheduled date, it can be made up for within a maximum of 4 weeks.

### Study assessments and procedures

#### Outcome and other non-safety assessments

**Fig. 1:**
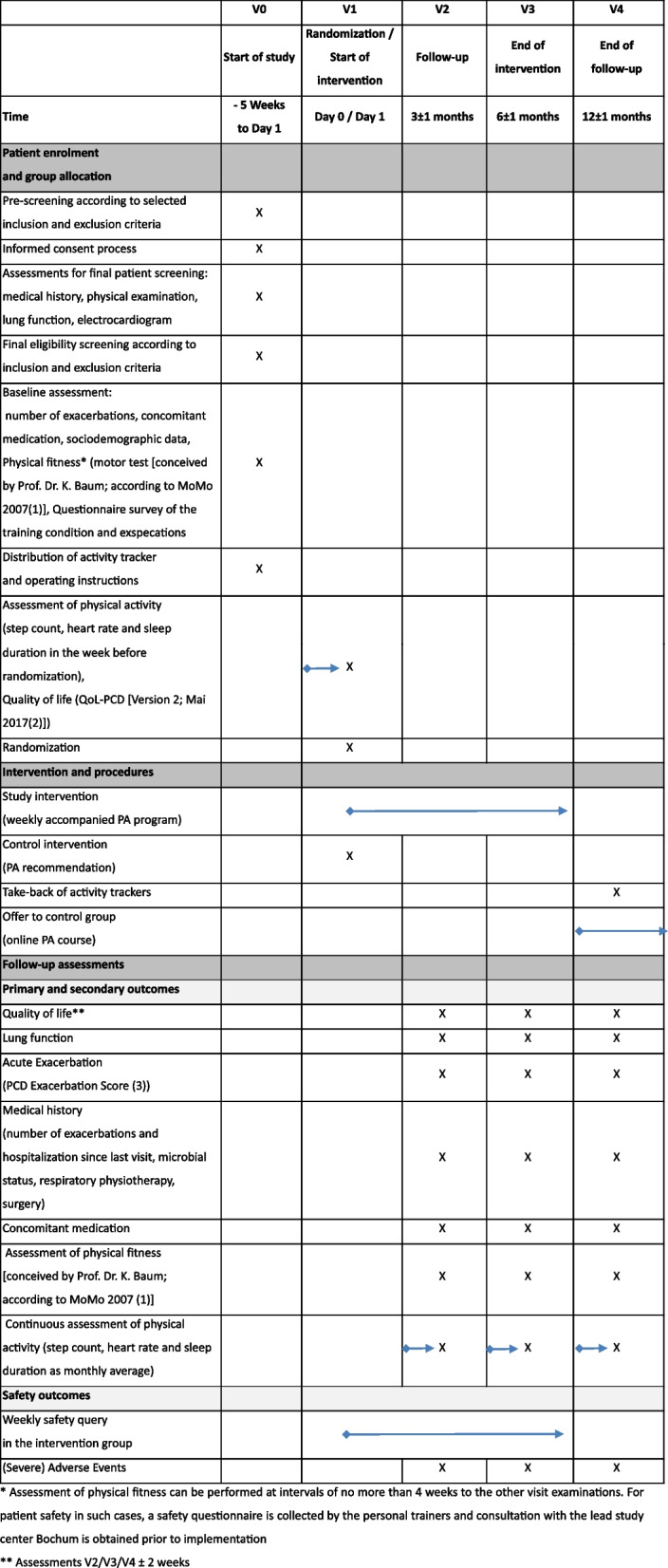
Schedule of activities {SPIRIT figure}. *Assessment of physical fitness can be performed at intervals of no more than 4 weeks to the other visit examinations. For patient safety in such cases, a safety questionnaire is collected by the personal trainers and consultation with the lead study center Bochum is obtained prior to implementation. **Assessments V2/V3/V4 ± 2 weeks

The data collected during the study visits (see Fig. 1), as described in detail below, and those of the PA are collected and managed using central databases. The Department of Medical Informatics, Biometry and Epidemiology carries out regular data monitoring. Prior to the start of recruitment, all centers and the training institute Prof. Dr. Baum receive an initiation including appropriate training measures for data collection, entry, and management using the central databases.

1. Medical history, exacerbation, and physical examination

The medical history will be asked by a questionnaire, including demographic data as well as current medication and therapy. In addition, the PCD exacerbation score [29] will be assessed. A physical examination including height, weight, and BMI is also carried out. The survey and the examination are repeated after 3, 6, and 12 months.

2. Spirometry

PCD is a disease that can affect the lower airways to varying degrees. Spirometry can be used to measure the current lung or respiratory volume. Usable results can be obtained from the age of 5 years. In PCD and other chronic lung diseases, such as bronchial asthma, regular lung function measurements are routine and guide treatment recommendations. Age-adapted standard values exist.

The following values are determined [32, 33]:

- VCmax (maximum vital capacity)
- FVC (forced vital capacity)
- FEV1 (forced expiratory volume in 1 s)
- PEF (maximum expiratory flow or peak flow)
- MEF 75/25 (maximum expiratory flow at 75/25% of FVC)
- sReff (specific airway resistance), if available
- TLC (total lung capacity), if available

The examination is repeated after 3, 6, and 12 months. If the lung function measurement coincides with a routine examination, the values from the body plethysmography carried out in this context can alternatively be taken over. At the beginning of the study, the patients are asked to sign a confidentiality agreement for this purpose, which allows the University Children’s Hospital Bochum to request the results of the lung function measurement from the treating physician.

Whenever technically possible, the Lung Clearance Index (LCI) is additionally recorded as part of the lung function examination. The method is established in diagnostics and therapy control in PCD [34–36].

3. Electrocardiogram (ECG)

To identify possible cardiac diseases that could hinder exercise, all test persons will receive a 12-lead ECG at the beginning. This is to detect cardiac arrhythmias, indications of serious conditions such as heart attacks, coronary heart disease, and signs of cardiac stress before the start of the intervention. The examination is only done once at baseline.

4. Assessment of physical fitness (motor test)

The motor test consists of 6 items: standing long jump, push-ups, standing on one leg, sit-ups, forward bend, 10-min run. Most of the components of the motor test have already been administered to children as part of the KIGGS study in the 2007 MoMo project and are thus readily applicable to the assessment of strength, endurance, and mobility in our study population aged 7 years and older [37]. The motor test will be done at the beginning and after 3, 6, and 12 months. The motor test and the other examinations within the framework of one study visit must not be more than 4 weeks apart. If the motor test does not take place on the same day as the other examinations of the study visit, safety questions will be asked before to ensure that the motor test can be carried out in a medically safe manner.

- Standing long jump

The test person stands with both feet on the starting line in a shoulder-width stance. Both feet are jumped off and landed at the same time; the distance to the heel of the rear leg is scored. Active arm swing is allowed. The best of three jumps is scored. Measurements are taken in centimeters.

- Push-up

The test person starts with the chest at the height of two Theraband stability trainers (ThS) the body is stretched, and the tips of the feet touch the floor shoulder-width apart. The arms are bent with the body stretched until the chest touches the thighs and then stretched again. Bending and stretching are done without rhythm. The number of valid attempts within 30 s is measured.

- Single-leg stand

The test person stands at 2 m facing the black sheet and fixes it with the eyes. The time is measured standing on one leg without the playing leg touching the ground or the standing leg moving. Otherwise, evasive movements are allowed. The start is made by lifting the playing leg, after 60 s it is stopped. Both legs are first tested with eyes open, in case of premature termination a second attempt is allowed for each leg. Subsequently, the same test procedure is carried out with blinding by means of a blindfold.

- Sit-up

The test person lies on the back with the knees bent parallel (90°), the head resting on the ThS, and the arms stretched out beside the body. The feet are fixed by the test leader. During the test, the upper body is raised until both hands touch the ankles and lowered again until the head touches the stability trainers. The number of completed exercises within 30 s is counted.

- Forward bend

The test person stands on a small gymnastic box with his knees stretched out and bends his upper body as far forward and downward as possible in this position. The measurement is taken at the level of the tip of the middle finger without bobbing.

- Ten-minute run

The test person runs indoor on a 65-m lap marked with 10 pylons for 10 min on a start signal. The goal is to cover the greatest possible distance. Alternating between running and walking is allowed. The test leader counts the laps and measures the incomplete remaining lap after completion. The distance covered is recorded (= number of laps × 65 m + remaining distance in m).

5. Physical activity

All participants will be given an activity tracker (Garmin Vivofit 4®, instruction manual included) for the study period right at the beginning. The handing over takes place in the context of the first motor test by the personal trainers of the training institute Prof. Dr. Baum. They check the correct wear of the activity tracker and assist with the setup. An activity tracker is given to both groups (interventional and control group) to avoid a bias by wearing an activity tracker. The activity tracker is to be worn day and night. The data is automatically synchronized with a central database and stored there in pseudonymized form for data evaluation.

In addition, current PA activity and training wishes are asked through questionnaires as part of the screening visit. The results, together with the results of the motor test, are used to develop the individualized training plan in the intervention group.

6. QoL questionnaire

The questionnaire is an established, validated instrument and used as an outcome parameter in several studies and in the international PCD registry [38, 39]. The QoL-PCD exists for all ages and contains a child version (37 items, 7 scales), parent proxy version (41 items, 9 scales), adolescent version (43 items, 7 scales), and adult version (49 items, 10 scales) [38–40]. The QoL-PCD scales include physical functioning (primary outcome); the other ones are secondary endpoints (e.g., emotional functioning, treatment burden, social functioning, role functioning, health perception, vitality). With the QoL-PCD, we choose an internationally validated PCD specific tool for different age groups [38, 39]. Filling in the questionnaire takes little time and will be tablet based or may be done at home via an individual link. One week before randomization, the participants and, in the case of minors, also the guardians receive a link with which they answer the questionnaire directly online in the central database. In addition, the questionnaires are recorded during the V2–V4 visits after 3, 6, and 12 months.

#### Safety assessments

Participant safety is taken very seriously in this study.

The individual risk in the study corresponds to the usual risk of PA which is recommended by guidelines as part of the standard therapy. This includes injuries, overuse injuries, cardiovascular events, and psychological risks such as sport addiction [22, 23, 41–44]. Other rare risks—seen sporadically in studies with PA in pwCF (or asthma bronchiale [24])—could be the occurrence of pneumothoraxes, when starting physical activity too early after infections, and other pulmonary complications like bronchial obstruction or hemoptysis [8, 24]. However, the increase in PA also offers many advantages for pwPCD.

Study participants are given the possibility to improve their health-enhancing PA by regular qualified support, and thus to improve their lung function, physical fitness, and participation in everyday life [6, 9, 43, 45–47]. In the middle term, we expect an improvement in treatment adherence and quality of life with a consequent slowing of the progression of the chronic disease PCD. The long-term benefit for all pwPCD would be the establishment of guided PA in the therapy and rehabilitation guidelines. In the risk–benefit analysis, the benefits for the participating patients clearly outweigh the risks.

To minimize the risks for participating patients, they are examined in detail for contraindications to participation in a PA program (see schedule of activities and “Exclusion criteria”) and receive comprehensive information when not to take part in PA. In addition, the focus of this study is on health-enhancing, individually adapted physical activity and not on physical exertion. The participants do the PA they prefer at their own pace under the close guidance of the qualified personal trainer. For this reason, the intervention group is questioned weekly by means of a standardized questionnaire about the occurrence of potentially critical complaints in the context of PA by the respective personal trainer. If one of the participants answers at least one of the questions in the lower third of the Numeric Analog Scale [1–4, 21, 29, 37, 40, 48, 49], which is handed out and explained at V0, PA will be discontinued immediately until medical clearance by the lead study center in Bochum is obtained. A standardized safety questionnaire must also be completed before the motor test that is part of the study visits. If there are indications of a medical concern, the lead study center Bochum will be contacted and if there is any doubt the test will not be performed.

An assessment of adverse events (AE) and serious adverse events (SAE) will be performed in the intervention and control group to evaluate the safety of the study intervention. For further details see below and under “Safety oversight.”

#### Adverse events and serious adverse events

In the following, we define the AEs and SAEs to which we are limited in this study due to the chosen intervention and the clinical characteristics of PCD. They comprehensively recognize both the expected risks and serious complications.

Adverse event (AE)

- Any unplanned doctor contact
- Pulmonary exacerbation or other pulmonary complications (obstruction, etc.)
- New antibiotics
- Accident during physical activity without hospital contact
- Medically justified discontinuation of physical activity (single occurrence)
- Discontinuation of intervention due to a medical cause

Serious adverse event (SAE)

- Death
- Any unplanned hospitalization or hospital presentation

For adverse events (AEs), the following guidelines will be used to describe severity.

- Mild—Events require minimal or no treatment and do not interfere with the participant’s daily activities.
- Moderate—Events result in a low level of inconvenience or concern with therapeutic measures. Moderate events may cause some interference with functioning.
- Severe—Events interrupt a participant’s usual daily activity and may require systemic drug therapy or other treatment. Severe events are usually potentially life-threatening or incapacitating. Of note, the term “severe” does not necessarily have to be identical with “serious.”

All adverse events (AEs) will have their relationship to study procedures, including the intervention, assessed by appropriately trained clinicians (pediatric and internal medicine) based on temporal relationship and his/her clinical judgment. The degree of certainty about causality will be graded using the categories below.

- Related—The AE is known to occur with the study procedures, there is a reasonable possibility that the study procedures caused the AE, or there is a temporal relationship between the study procedures and the event. Reasonable possibility means that there is evidence to suggest a causal relationship between the study procedures and the AE.
- Not related—There is not a reasonable possibility that the study procedures caused the event, there is no temporal relationship between the study procedures and event onset, or an alternate etiology has been established.

During each study visit (V2–V4), a standardized query (CRF) is made by means of questionnaires of the AEs and SAEs defined above. In addition, all participants are encouraged to report unplanned hospitalizations (SAEs) to their supervising study center within 48 h.

Information to be collected includes event description, time of onset, clinician’s assessment of severity, relationship to study procedures (assessed only by those with the training and authority to make a diagnosis), and time of resolution/stabilization of the event. All AEs occurring while on study will be documented appropriately regardless of relationship. All AEs will be followed to adequate resolution.

All (S)AEs are documented in the central study database by the responsible study center. If an AE or SAE is recorded in the central database, the study team of the lead study center Bochum is automatically informed. In addition, the SAE report can be forwarded to the lead study center via fax. The study team will then contact the registering study center and the subject to obtain further information. In the case of SAEs, the SAE board is immediately involved. The intervention will be interrupted until the final clarification of whether there is a risk for the participant by continuing the intervention. The lead study center in Bochum will inform the participants as soon as possible whether the intervention can be resumed.

The lead study center Bochum will record events with start dates occurring any time after informed consent is obtained until the last day of study participation. The documents are submitted to the DSMB once a year for further review.

The local study management at each site is responsible for truthfully documenting all AEs and SAEs during study visits and for notification also in between in the central database. The lead study center in Bochum and the PI receives an automated notification from the central database upon registration and must check it immediately. SAE reports are forwarded to the SAE board within 72 h. After the final assessment of the event, the documents are collected and forwarded bundled to the DSMB once a year by the PI or a named representative of the leading study center in Bochum. In the event of an accumulation of study-relevant SAEs, early convening of the DSMB and notification to the relevant ethics committee will be made by the PI. The assessment of the event is documented in the central database by the study team at the Bochum University Children’s Hospital.

As part of the underlying disease, pulmonary exacerbations and associated doctor contacts or unplanned hospital stays are common. If this constellation emerges during the examination by the central study center, it is not necessary to involve the SAE board for verification. The assessment and documentation are then carried out by the study team from Bochum directly in the central database. The DSMB is regularly informed by the annual report. If there is an increase in pulmonary exacerbations in a study center or the study, the PI or a named representative is obliged to inform the SAE board separately within 72 h. Depending on the assessment of the SAE board, an early notification to the DSMB as well as to the ethics committee responsible will also be made by the PI in this case.

After assessing the event, the study team from Bochum will contact the participant by telephone to discuss further procedures regarding the study. If pathological findings are detected during the study visits, the participant will be informed directly orally about the findings and a recommendation for further (subject-specific) clarification will be made. If requested, the participant may be provided with a copy of the examination results for the doctor providing further treatment.

#### Unanticipated problems

This protocol uses the definition of unanticipated problems as defined by the Office for Human Research Protections (OHRP). OHRP considers unanticipated problems involving risks to participants or others to include, in general, any incident, experience, or outcome that is unexpected in terms of nature, severity, or frequency. That furthermore is elated or possibly related to participation in the research and suggests that the research places participants or others at a greater risk of harm (including physical, psychological, economic, or social harm) than was previously known or recognized. All serious medical unanticipated problems are captured by the AE and SAE notifications and reported accordingly. All other unanticipated problems are documented. Participants will be informed about UPs on an individual or aggregate level as appropriate.

#### Participant timeline

See Fig. 2.

**Fig. 2:**
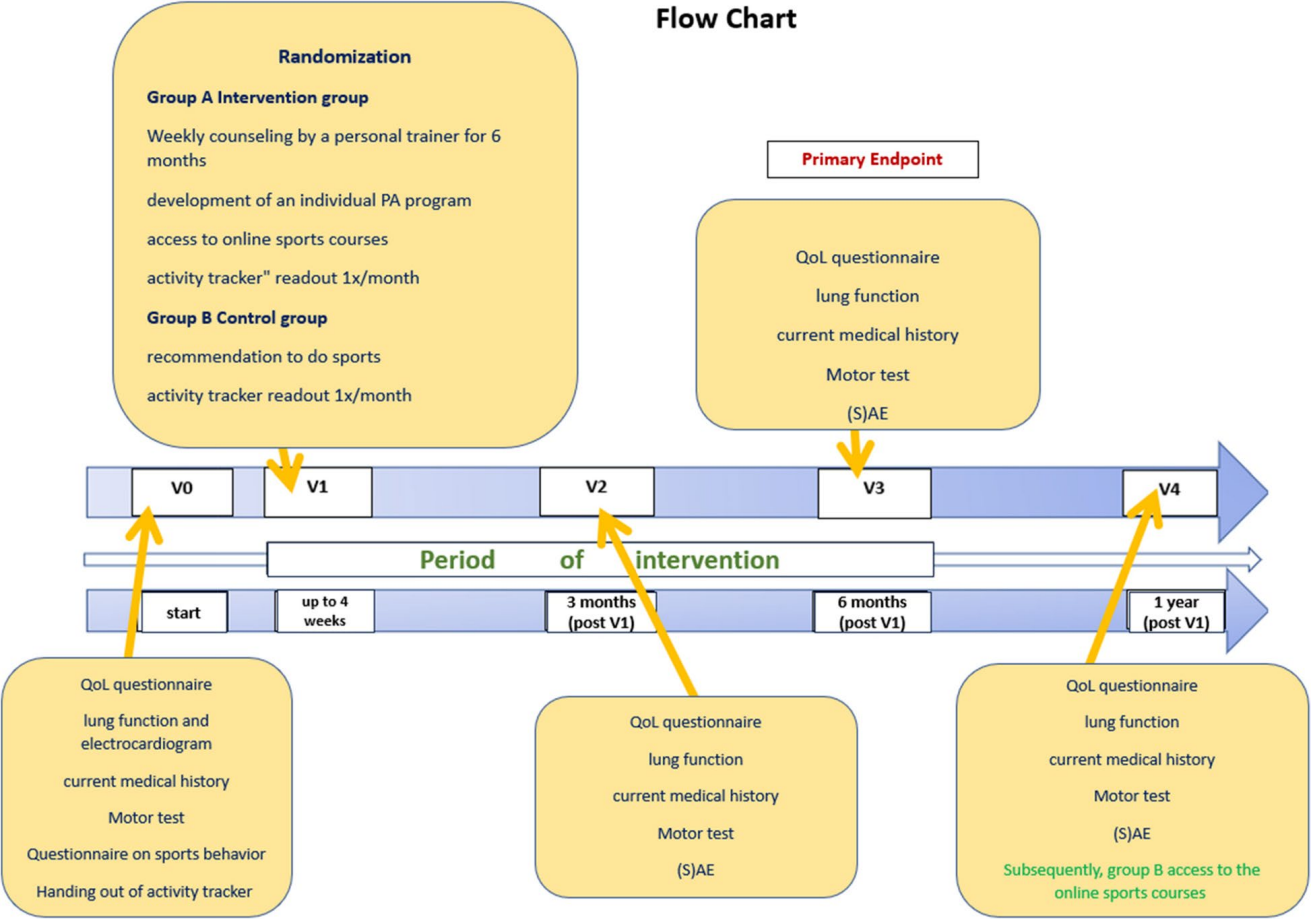
Timeline for participants

#### Sample size determination

For the physical domain of the QoL-PCD 15.2 points were proposed as minimum clinically significant change, the observed standard deviation (sd) in a similar patient group was 30.4 [38]. Sample size calculation for a two-sided two-sample *t*-test at a significance level (*α*) of 5% resulted in a required sample size of 64 per group to reach a power of approximately 80% to detect an effect size of 0.5 (15.2/30.4). Assuming a drop-out rate of 5%, 158 participants should be recruited.

PCD is a rare disease for which there are few specialists. Medical research in PCD and especially in therapy has made little progress to date. Many pwPCDs therefore have a great interest in clinical research and a close bond with their practitioners and travel long distances to do so. We can therefore expect a high level of compliance.

#### Measures to minimize bias: randomization and blinding

To guarantee adequate sequence generation and allocation concealment, centralized online randomization with 1:1 allocation to intervention or control group is done through REDCap. A person at the AMIB not further involved in the study creates randomization lists based on permuted blocks of random length and are stratified by age group (6–12, 13–17, ≥ 18 years) and FEV1 (≤ / > 60%) to guarantee nearly equal group sizes within the strata. Allocation concealment will be ensured, as the service will not release the randomization code until the patient has been entered into the trial, which takes place after all baseline measurements have been completed.

It is not feasible nor possible for the participating patients and most project staff to be blinded to treatment allocation. Primary outcome assessment will be based on patient reports. Staff who analyze the data will be blinded (arms will be designated randomly at that point as A and B). Data review will be performed blinded.

#### Populations for analyses

Three differing data sets are defined for the analyses:

- Full analysis set

The full analysis set (FAS, based on the intention-to-treat (ITT) strategy) is defined to be all randomized patients according to their allocated intervention group, which fulfill the inclusion criteria and do not fulfill any exclusion criteria.

b) Per protocol set

The per protocol set (PPS) is defined by all patients belonging to the FAS without major violations of the study protocol. The following protocol violations are classified as major:

- Violation of an eligibility criterion
- Discontinuation of intervention for non-medical reasons
- Non-compliance

This is not an exclusive list. The full list of all major protocol violations, which will lead to the exclusion from the PPS, will be defined as part of the statistical analysis plan in the light of protocol violations which occur during study conduct. The final list of protocol violation will be finished before database closure and the beginning of the statistical analysis.

c) Safety analysis dataset

The safety analysis set will include all randomized patients.

### Statistical analyses

Statistical analysis will be performed using the statistical software R (http://www.r-project.org) version 4.1.0 or higher. Data scientists will remain blinded using non-meaningful group labels, e.g., the groups will be called “group A” and “group B.” For descriptive statistics, continuous variables will be summarized using the arithmetic mean and standard deviation; in case of skewed distribution, median and interquartile range are used. Categorical variables will be summarized using total counts and percentages.

Unless otherwise stated, the estimate with corresponding two-sided 95% confidence interval and two-sided *p* value will be reported for all confirmatory and exploratory analyses. *p* values less than 5% will be considered statistically significant. Details of the statistical analysis of primary and secondary endpoints, imputation of missing data, descriptive and additional analyses will be later specified in the statistical analysis plan (SAP). The following paragraphs give a basic overview of statistical procedures.

#### Analysis of the primary endpoint

The main aim of the trial is to estimate the difference in the change of quality of life between the intervention and control after 6 months in the domain of physical function. The confirmatory analysis will be conducted in the FAS based on the ITT principle. The analysis of the primary endpoint is carried out using a linear model. In addition to the intervention group, the model also includes baseline FEV1, gender and the interaction between age group, and baseline quality of life as independent variables. Estimated difference and corresponding two-sided confidence interval will be reported. In case of missing primary outcome, multiple imputation will be done. Missing values will be imputed from baseline and all available data after baseline.

#### Analysis of the secondary endpoint(s)

Analysis of all secondary outcomes will be done as complete case analysis, meaning that only cases with available data for the analysis are included. Analysis of the secondary efficacy outcomes will be undertaken following the same framework as the primary outcome model using appropriate generalized linear models.

For each continuous outcome, e.g., FEV1 and QoL-PCD, a linear regression model will be fitted, the intervention group, gender, FEV1 and the age group and the baseline value of the outcome considered were included as independent variables. For the QoL-PCD, an interaction term with age group is also added to the model.

For counting variables like number of pulmonary exacerbations, a similar generalized linear model will be fitted using a negative binomial distribution with a log link instead. Treatment effects are reported as relative risk with 95% confidence intervals.

#### Safety analyses

Analysis of safety endpoints will be performed in the safety analysis set. Adverse events will be analyzed as number and percentage per group. Events leading to a discontinuation of the intervention or withdrawal from the study will be tabulated.

#### Baseline descriptive statistics

Demographic and baseline variables will be summarized in a table, stratified by treatment and control group. Continuous variables will be summarized using the arithmetic mean and standard deviation; in case of skewed distribution, median and interquartile range is used. Categorical variables will be summarized using total counts and percentages. No inferential statistics will be used to compare baseline characteristics.

#### Additional analyses

No tabulation of individual participant data will be done. No interim analysis will be performed. In addition, it is planned to conduct exploratory mediator analysis using the fitness tracker data to further investigate the mode of action of the intervention.

CCDC39/40 and CCNO gene variants in particular are associated with more rapid disease progression [50]. Participants with these mutations are therefore analyzed separately as a subgroup. Instead of further subgroup analyses, explorative moderator analyses are planned. Possible moderators for the intervention effect on change in quality of life are age group (children, adolescents, and adults), the motor test at baseline, genetic variant, and baseline FEV1. Moderator analyses will be performed using the model from the primary endpoint analysis with an additional inclusion of the treatment × moderator interaction.

### Data management and protection

In the study, personal data of the patients will only be processed in compliance with the relevant data protection regulations, i.e., only to the extent that this is necessary to conduct the study. The processing of patients’ personal data is only carried out after their verbal and written consent for data capture, transmission, and analysis. The patients are informed of this fact and agree to the procedure with the patient information/informed consent. Data captured by the investigators will be processed in such a way that it can be accurately reported, interpreted, and verified while the confidentiality of records and the personal data of the subjects remain protected. Data capture and processing will be in accordance with the applicable law on personal data protection and with the GDPR (EC) 2016/679 of the European parliament and of the council. Access to the data is strictly limited to authorized persons. Data are protected against unauthorized access. Study participant contact information will be securely stored at each clinical site for internal purposes and study evaluations, as well as for confidential use by the lead study center (e.g., contacting in the case of an SAE) or by the sports scientists (e.g., contacting for telephone interviews) during the study.

The investigators together with the trial sites are responsible for the implementation and data processing in accordance with Article 4(7) of the General Data Protection Regulation 2016/679 in this trial. The investigators are responsible for implementation of procedures for data collection, storage, protection, retention, and destruction. The database is hosted by the AMIB on servers, which are located at the RUB in access-protected server rooms.

Personal data collected for this study will be stored for 10 years from completion of the study. When the study is completed, access to study data will be provided upon request to the PI. All other data collected as part of this study will be analyzed and stored in a separate study database only with the pseudonymization number for at least 10 years after last publication. After the personal data has been deleted, the data is anonymized. Access to the final complete dataset is available to the study staff of the AMIB and the leading study center of the University Children’s Hospital Bochum as well as Prof. Klaus Baum. Study teams from other study centers will only have access to the complete dataset upon request and approval by the Publication Committee.

All data will be initially collected by investigators in the recruiting trial sites. Together with information on the trial, eligible patients will be informed about data capture, transmission, analysis processes, and their rights according to the General Data Protection Regulation (GDPR). Once a patient is eligible and has given his/her informed consent (concise version) to trial participation and data collection, the investigator will assign with the help of a separate “contact database” the patient a unique patient identification code which is generated by AMIB. The personal data and declarations of consent of the participants are stored in the separate contact database. These codes are part of the investigator site file. Re-identification of the patients is only possible with the help of the contact database. Access is only possible for authorized persons. The authorization levels depend on the function in the study. Study centers only see the subjects they have included. The leading study center and the training institute Prof. Dr. Baum (for the study period) will have access to all subjects to ensure scheduling, implementation of the intervention, and safety management.

All clinical data entered in the trial database by the investigators (or their designated staff) or patients into eCRFs will be recorded in a pseudonymized form exclusively using the patient’s identification code.

Patients may withdraw their informed consent. If patients withdraw their consent, no further data will be collected. However, the data processing carried out up to the date of withdrawal remains lawful. If the informed consent is withdrawn, the patient has the right of data deletion according to the GDPR. Information as to when and why a patient was randomized and when he withdrew consent must be retained in the documentation. According to GDPR, the institution responsible for conducting the study is generally obliged to delete its data from the study database after withdrawal of consent. According to the basic data protection regulation, the study participants have the following rights regarding their personal data:

1. The right to be informed about their personal data that are collected, processed or, if applicable, transferred to third parties in the course of the clinical trial (if necessary, handing out a copy free of charge).
2. Right to have incorrect personal data corrected. Right to have their personal data deleted, with the exception of the security data described above.
3. Right to limit processing (in exceptional cases). The right to limit processing must be requested from the investigator or the data protection officer of the trial site.
4. Right to data transfer of personal data collected about the study participant. This data shall be transmitted either to the trial participant himself or, if technically possible, to another body designated by the trial participant.
5. Right to refuse (conditionally) the use of the data (see also right to cancelation).

#### Data collection and management responsibilities

Data collection will be the responsibility of the clinical trial staff at the site under the supervision of the site investigator. The investigator will be responsible for ensuring the accuracy, completeness, legibility, and timeliness of the data reported.

All source documents will be completed in a neat, legible manner (digital whenever possible) to ensure accurate interpretation of data. Clinical data (including concomitant medications, therapies, or other health-related aspects) will be entered into REDCap® (trial database), an electronic data capture system provided by the Department of Medical Informatics, Biometry and Epidemiology. The data system includes password protection and internal quality checks, such as automatic range checks, to identify data that appear inconsistent, incomplete, or inaccurate.

#### Study records retention

Anonymization, in the sense of deletion of personal data, takes place 10 years after the end of the study. Study documents will be retained for at least 10 years after the last publication. No records will be destroyed without the written consent of the principal investigator, if applicable.

### Data monitoring

#### Safety oversight

This is a multi-center study. Security is monitored centrally. There is a Data and Safety Monitoring Board (DSMB) that meets once a year and evaluates all AE and SAE reports. If there is an accumulation of study-related SAEs, the DSMB is convened early. The DSMB is composed of individuals with the appropriate expertise, including a biostatistician, pediatric pneumologists, and patient representative. Members of the DSMB will be independent from the study conduct and free of conflict of interest. The DSMB will provide its input to the financial sponsor of the study.

In addition to the DSMB, there is a study-independent “SAE board.” The members are specialists in the pediatric and internal medicine fields. This board evaluates events that led to a discontinuation of the intervention in the short-term for the study context and decides whether the intervention can be resumed without hesitation.

#### Clinical monitoring

Clinical site monitoring will be conducted to ensure that the rights and well-being of trial participants are protected; that the reported trial data are accurate, complete, and verifiable; and that the conduct of the trial is in compliance with the currently approved protocol/amendment(s), with International Council on Harmonisation Good Clinical Practice (ICH GCP), and with applicable regulatory requirement(s).

#### Quality assurance and quality control

Each clinical site will perform internal quality management of study conduct, data collection, documentation, and completion. All sites will follow a common quality management plan.

Quality control procedures will include:

- Informed consent

Study staff will review both the documentation of the consenting process as well as a percentage of the completed consent documents. This review will evaluate compliance with GCP, accuracy, and completeness. Feedback will be provided to the study team responsible for ensuring proper consenting procedures are followed.

b) Intervention fidelity

Consistent delivery of the study interventions will be monitored throughout the intervention phase of the study. Procedures for ensuring fidelity of intervention delivery are described in “Fidelity—interventionist training and tracking.”

c) Protocol deviations

The study team will review protocol deviations on an ongoing basis and will implement corrective actions when the quantity or nature of deviations is deemed to be at a level of concern.

This protocol defines a protocol deviation as any non-compliance with the clinical trial protocol or ICH GCP requirements. The non-compliance may be due to either the participant, the investigator, or the study site staff. In the event of non-compliance, corrective actions are developed and promptly implemented by the trial site. These practices are in accordance with ICH GCP. Non-compliance is defined as follows:

- Particularly noteworthy are gross violations of safety by the study centers or the intervention-carrying employees of the training institute Prof. Dr. Baum, such as conducting study investigations without written consent for study participation.
- Refusal of the intervention is considered non-compliance for participants.

It will be the responsibility of the site investigator to use continuous vigilance to identify and report deviations within 7 working days of identification of the protocol deviation, or within 7 working days of the scheduled protocol-required activity. All deviations will be addressed in study source documents and reported to the Data Coordinating Center.

### Study discontinuation and closure

This study may be temporarily suspended or prematurely terminated if there is sufficient reasonable cause. Written notification, documenting the reason for study suspension or termination, will be provided by the suspending or terminating party to study participants, investigator, and funding agency. If the study is prematurely terminated or suspended, the principal investigator (PI) will promptly inform study participants, the EC, and sponsor/funding agency and will provide the reason(s) for the termination or suspension. Study participants will be contacted, as applicable, and be informed of changes to the study visit/assessment schedule.

Circumstances that may warrant termination or suspension include, but are not limited to:

- Determination of unexpected, significant, or unacceptable risk to participants
- Development of new evidence regarding the content of the study outside of the study during the study period
- Poor recruitment

The study may resume once concerns about safety, protocol compliance, and data quality are addressed and satisfy the funding agency, EC, and steering committee.

## Discussion

This multi-center study is the first to investigate the effects of supported physical activity in pwPCD on quality of life. The evaluation of individual changes in quality of life in both the intervention and control groups and the stratification in the context of central randomization counteract possible biases due to the large age spectrum, center effects, and the very different disease severity. In addition, the analysis of individual changes in quality of life in combination with activity tracking also offers the possibility of classifying a possible increase in PA in the control group and its effects in the evaluation. In order to counteract frustration and thus an increased drop-out rate in the control group, the control group receives monthly reminder and email of thanks in addition to the activity tracking, as well as access to the online videos after the end of the study.

## Trial status

Recruitment started on March 5, 2024, and is expected to end on March 31, 2025. The period from first patient in to last patient out is planned to be 24 months. The trial is based on the study protocol version 1.1 dated November 10, 2023. The protocol was updated in June 2024 (version 1.2).

### Supplementary Information

Supplementary Material 1.

Supplementary Material 2.

Supplementary Material 3.

Supplementary Material 4

Supplementary Material 5

Supplementary Material 6

Supplementary Material 7.

Supplementary Material 8

## Data Availability

The datasets analyzed during the current study and statistical code are available from the corresponding author on reasonable request, as is the full protocol. Data access request will be reviewed by the study team. Data will not be published on open data repository due to the inclusion of children and adolescents with a heterogeneous rare disease with a high risk of re-identification.

## Abbreviations

AE: Adverse event
CF: Cystic fibrosis
CONSORT: Consolidated Standards of Reporting Trials
COPD: Chronic obstructive pulmonary disease
CRF: Case report form
DCC: Data Coordinating Center
DSMB: Data Safety Monitoring Board
EC: Ethics committee
eCRF: Electronic case report form
ERS: European Respiratory Society
FAS: Full analysis plan
FEV1: Forced expiratory volume in 1 s
FVC: Forced vital capacity
GCP: Good Clinical Practice
ICD: Implantable cardioverter defibrillator
ICH: International Council on Harmonisation
ITT: Intention-to-treat
LCI: Lung Clearance Index
MEF 75/25: Maximum expiratory flow at 75/25% of FVC
NIV: Non-invasive ventilation
OHRH: Office for Human Research Protections
PA: Physical activity
PCD: Primary ciliary dyskinesia
PEF: Maximum expiratory flow or peak flow
PI: Principal investigator
PPS: Per protocol set
PTCA: Percutaneous transluminal coronary angioplasty
pwPCD: Persons with PCD
QoL: Quality of life
RCT: Randomized controlled trial
SAE: Serious adverse event
SAP: Statistical analysis plan
sReff: Specific airway resistance
TLC: Total lung capacity
VCmax: Maximum vital capacity
V0–4: Screening and study visits
