# Supplementary material for: Move-PCD—a multi-center longitudinal randomized controlled superiority trial on the effect of a 6-month individualized supported physical activity (PA) program on quality of life (QoL) in children, adolescents, and adults with primary ciliary dyskinesia
Source: Trials. 2024 Aug 15;25:539. doi: 10.1186/s13063-024-08379-0 (PMC11328395; doi:10.1186/s13063-024-08379-0)
Supplement: Supplementary file 1 — Supplementary Material 1. [file 13063_2024_8379_MOESM1_ESM.pdf]

DLR Projektträger, Heinrich-Konen-Straße 1, 53227 Bonn

Ruhr-Universität Bochum
z.H. Frau Prof. Folke Brinkmann
Universitätsstraße 150
44801 Bochum

DLR Projektträger
Bereich Gesundheit

Ihre Ansprechpartner:
Wissenschaftliche/r Mitarbeiter/in Dr. Katarzyna Saedler
Telefon +49 228 3821-1947
E-Mail Katarzyna.Saedler@dlr.de
Administrative/r Mitarbeiter/in Viktoria Quindt-Wotzke
Telefon +49 228 3821-3023
E-Mail Viktoria.Quindt-Wotzke@dlr.de
Telefax +49 228 3821-1257

Datum 19.03.2024

To whom it may concern

Dear Sir or Madam,

This is to confirm that the clinical study „Move-PCD - Eine multizentrische randomisierte, kontrollierte longitudinale Studie der Auswirkung eines sechsmonatigen individualisierten und überwachten Aktivitätsprogrammes auf die Lebensqualität bei Kindern, Jugendlichen und Erwachsenen mit primärer ziliärer Dyskinesie (PCD)“, Principal Investiator Prof. Folke Brinkmann is funded by the German Federal Ministry of Education and Research (BMBF).

Please do not hesitate to contact us in case of further questions.

Sincerely,

i.A. i.A.

Dr. Katarzyna Saedler Viktoria Quindt-Wotzke
