# Supplementary material for: Move-PCD—a multi-center longitudinal randomized controlled superiority trial on the effect of a 6-month individualized supported physical activity (PA) program on quality of life (QoL) in children, adolescents, and adults with primary ciliary dyskinesia
Source: Trials. 2024 Aug 15;25:539. doi: 10.1186/s13063-024-08379-0 (PMC11328395; doi:10.1186/s13063-024-08379-0)
Supplement: Supplementary file 2 — Supplementary Material 2. [file 13063_2024_8379_MOESM2_ESM.pdf]

Ethik-Kommission der Med. Fakultät der RUB
Gesundheitscampus 33, 44801 Bochum

RUHR UNIVERSITÄT BOCHUM RUB

**Ethik-Kommission
der Medizinischen Fakultät**

Telefon: 0234/7981 6555
Telefax: 0234/7981 6556
Gesundheitscampus 33, 44801 Bochum
Email: ethik@rub.de
www.ruhr-uni-bochum.de/ethik

St. Josef-Hospital
Universitätsklinik für Kinder- und Jugendmedizin
Prof. Dr. med. Thomas Lücke
Alexandrinenstr. 5
44791 Bochum

44801 Bochum, den 04.12.2023

Antrag vom 25.09.2023 **vollständig eingegangen** am 26.09.2023

Registrier - Nr.:	**23-7938-andere Forschung erstvotierend** (**Bei Schriftwechsel bitte immer angeben!)**
Thema:	Move-PCD Eine multizentrische randomisierte, kontrollierte longitudinale Studie der Auswirkung eines sechsmonatigen individualisierten und überwachten Aktivitätsprogrammes auf die Lebensqualität bei Kindern, Jugendlichen und Erwachsenen mit Primärer Ciliärer Dyskinesie (PCD)
Untersucher:	Dr. med. Anne Schlegtendal, Prof. Dr. med. T. Lücke
Abteilung:<br>Klinik:	Universitätsklinik für Kinder- und Jugendmedizin<br>St. Josef-Hospital<br>Alexandrinenstraße 5, 44791 Bochum

Sehr geehrter Herr Professor Lücke, sehr geehrte Frau Professor Brinkmann,

die Ethikkommission der Medizinischen Fakultät der Ruhr-Universität Bochum hat die o.g. Studie in ihrer 340. Sitzung am 10.10.2023 beraten. Die zusätzlich geforderten Informationen/Modifikationen sind am 13.11.2023 eingegangen, die Unterlagen wurden außerhalb eines Sitzungstermins erneut beraten.

Die Ethikkommission hat hinsichtlich des beantragten Untersuchungsvorhabens im Rahmen der Forschung am Menschen aufgrund der vorgelegten Unterlagen
keine Bedenken **[XX]**

Die Ethik-Kommission weist darauf hin, dass die ärztliche und juristische Verantwortung bei den jeweiligen Studienärzten verbleibt. Alle Änderungen im Prüfplan müssen der Ethik-Kommission vorgelegt werden und dürfen erst nach der zustimmenden Bewertung umgesetzt werden.

Sollte die Studie nicht innerhalb der im Antrag angegebenen Frist beginnen, bittet die Ethik-Kommission um Mitteilung.

Über alle schwerwiegenden oder unerwarteten, unerwünschten Ereignisse, die während der Studie auftreten und die Sicherheit der Studienteilnehmer oder die Durchführung der Studie beeinträchtigen könnten, muss die votierende Ethik-Kommission unverzüglich unterrichtet werden.

Die Ethik-Kommission weist ausdrücklich darauf hin, dass datenschutzrechtliche Aspekte von Forschungsvorhaben durch die Ethik-Kommission grundsätzlich nur kursorisch geprüft werden. Dieses

Votum / diese Bewertung ersetzt mithin nicht die Konsultation des zuständigen Datenschutzbeauftragten.

Die Ethik-Kommission der Medizinischen Fakultät der Ruhr-Universität Bochum arbeitet gemäß den nationalen gesetzlichen Bestimmungen und den ICH-GCP-Richtlinien.

Der Abschluss / Abbruch der Studie ist mitzuteilen, ein Abschlussbericht vorzulegen.
Die Kommission empfiehlt die Registrierung der Studie beim „Deutsches Register Klinischer Studien" (www.drks.de).

Bei späteren Publikationen muss die Reg.-Nr. sowie das Datum des Votums genannt werden.

Mit freundlichen Grüßen
Im Auftrag

Christine Uphues-Schnell
Geschäftsführende Ärztin und Mitglied der Ethik-Kommission
der Medizinischen Fakultät der **RU**Bochum

Folgende Unterlagen lagen der Beratung zugrunde:

1) AE Sheet_Bochum.pdf vom 25.09.2023
2) AE Sheet_clean.pdf vom 25.09.2023
3) Anamnesebogen_Bochum.pdf vom 25.09.2023
4) Anamnesebogen_clean.pdf vom 25.09.2023
5) Angebot Probandenversicherung 2023-09-08.pdf vom 25.09.2023
6) Angebot Versicherung Begelitpersonen.pdf vom 13.11.2023
7) Anschreiben Ethikantrag MOVE PCD.pdf vom 25.09.2023
8) Anschreiben Ethikkommission MOVE PCD_Nachforderung_signed.pdf vom 13.11.2023
9) Anschreiben MOVE PCD_26092023.pdf vom 26.09.2023
10) Aufklärung Eltern Move PCD_V1.1_Bochum.pdf vom 13.11.2023
11) Aufklärung Eltern Move PCD_V1.1_clean.pdf vom 13.11.2023
12) Aufklärung Eltern Move PCD_V1_Bochum.pdf vom 25.09.2023
13) Aufklärung Eltern Move PCD_V1_clean.pdf vom 25.09.2023
14) Aufklärung Erwachsene Move PCD_V1.1_Bochum.pdf vom 13.11.2023
15) Aufklärung Erwachsene Move PCD_V1.1_clean.pdf vom 13.11.2023
16) Aufklärung Erwachsene Move PCD_V1_Bochum.pdf vom 25.09.2023
17) Aufklärung Erwachsene Move PCD_V1_clean.pdf vom 25.09.2023
18) Aufklärung Jugendliche Move PCD_V1.1_Bochum.pdf vom 13.11.2023
19) Aufklärung Jugendliche Move PCD_V1.1_clean.pdf vom 13.11.2023
20) Aufklärung Jugendliche Move PCD_V1_Bochum.pdf vom 25.09.2023
21) Aufklärung Jugendliche Move PCD_V1_clean.pdf vom 25.09.2023
22) Aufklärung Kinder Move PCD_V1.1_Bochum.pdf vom 13.11.2023
23) Aufklärung Kinder Move PCD_V1.1_clean.pdf vom 13.11.2023
24) Aufklärung Kinder Move PCD_V1_Bochum.pdf vom 25.09.2023
25) Aufklärung Kinder Move PCD_V1_clean.pdf vom 25.09.2023
26) Begleitmedikation Move PCD.pdf vom 25.09.2023
27) Berichterstatter_HK_Nr 23-7938 .pdf vom 09.10.2023
28) Checkliste Telefontermine_Bochum.pdf vom 25.09.2023

29) Checkliste Telefontermine_clean.pdf vom 25.09.2023
30) CRF_Folgevisiten_Bochum.pdf vom 25.09.2023
31) CRF_Folgevisite_clean.pdf vom 25.09.2023
32) CRF_VO Screening_Bochum.pdf vom 25.09.2023
33) CRF_VO Screening_clean.pdf vom 25.09.2023
34) Datenschutzkonzept_10112023_Bochum.pdf vom 13.11.2023
35) Datenschutzkonzept_10112023_clean.pdf vom 13.11.2023
36) Datenschutzkonzept_25092023.pdf vom 25.09.2023
37) DATENSCHUTZRICHTLINIE FÜR GARMIN CONNECT.pdf vom 13.11.2023
38) DATENSCHUTZRICHTLINIE FÜR GARMIN JUNIOR.pdf vom 13.11.2023
39) DATENSCHUTZRICHTLINIE GARMIN.pdf vom 13.11.2023
40) Deaktivierung Google Analytics.pdf vom 13.11.2023
41) Ein und Ausschlusskriterien_ Bochum.pdf vom 25.09.2023
42) Ein und Ausschlusskriterien_clean.pdf vom 25.09.2023
43) Einverständnis Kontaktaufnahme für Homepage_Bochum.pdf vom 25.09.2023
44) Einwilligung Activitiy Tracker_Bochum.pdf vom 25.09.2023
45) Einwilligung Activitiy Tracker_Bochum_V1.1.pdf vom 13.11.2023
46) Einwilligung Activity Tracker_clean.pdf vom 25.09.2023
47) Einwilligung Activity Tracker_clean_V1.1.pdf vom 13.11.2023
48) Einwilligung Eltern Move PCD_V1.1_Bochum.pdf vom 13.11.2023
49) Einwilligung Eltern Move PCD_V1.1_clean.pdf vom 13.11.2023
50) Einwilligung Eltern Move PCD_V1_Bochum.pdf vom 25.09.2023
51) Einwilligung Eltern Move PCD_V1_clean.pdf vom 25.09.2023
52) Einwilligung Erwachsene Move PCD_V1.1_Bochum.pdf vom 13.11.2023
53) Einwilligung Erwachsene Move PCD_V1.1_clean.pdf vom 13.11.2023
54) Einwilligung Erwachsene Move PCD_V1_Bochum.pdf vom 25.09.2023
55) Einwilligung Erwachsene Move PCD_V1_clean.pdf vom 25.09.2023
56) Einwilligung Jugendliche MovePCD_Bochum.pdf vom 25.09.2023
57) Einwilligung Jugendliche MovePCD_clean.pdf vom 25.09.2023
58) Einwilligung Jugendliche MovePCD_V1.1_Bochum.pdf vom 13.11.2023
59) Einwilligung Jugendliche MovePCD_V1.1_clean.pdf vom 13.11.2023
60) Einwilligung Kinder Move PCD_V1_Bochum.pdf vom 25.09.2023
61) Einwilligung Kinder Move PCD_V1_clean.pdf vom 25.09.2023
62) Erklärung zur Dissertation.pdf vom 25.09.2023
63) Exacerbationsscore PCD_ Bochum.pdf vom 25.09.2023
64) Exacerbationsscore PCD_clean.pdf vom 25.09.2023
65) Fitrockr - Data Protection - for Projects.pdf vom 13.11.2023
66) Flyer MOVE PCD.pdf vom 25.09.2023
67) Fragebogen Aktivität und Trainingswünsche_Bochum.pdf vom 25.09.2023
68) Fragebogen Aktivität und Trainingswünsche_clean.pdf vom 25.09.2023
69) German Adolescent QOL-PCD V2_ offizielle Version.pdf vom 25.09.2023
70) German Adult QOL-PCD V2_offizielle Version.pdf vom 25.09.2023
71) IHRE DATENSCHUTZRECHTE GARMIN.pdf vom 13.11.2023
72) Information Kontrollgruppe_Bochum.pdf vom 25.09.2023
73) Information Kontrollgruppe_clean.pdf vom 25.09.2023
74) Kurzbeschreibung.pdf vom 25.09.2023
75) Motoriktest_Bochum.pdf vom 25.09.2023
76) Motoriktest_clean.pdf vom 25.09.2023
77) MovePCD - Antragsformular.pdf vom 25.09.2023
78) MovePCD - Antragsformular_26092023.pdf vom 26.09.2023
79) Move_PCD Protocol_final_signed.pdf vom 25.09.2023
80) Move_PCD Protocol_final_signed_V1.1.pdf vom 13.11.2023
81) Police Probandenversicherung.pdf vom 13.11.2023

82) Probandeninformation Aktivitätseinschränkung_Bochum.pdf vom 25.09.2023
83) Probandeninformation Aktivitätseinschränkung_clean.pdf vom 25.09.2023
84) QOL-PCD Child GERMAN_V2_offizielle Version.pdf vom 25.09.2023
85) QOL-PCD_Parent_Proxy_German V2_offizielle Version.pdf vom 25.09.2023
86) SAE Sheet_Bochum.pdf vom 25.09.2023
87) SAE Sheet_clean.pdf vom 25.09.2023
88) Schweigepflichtentbindung SAE_Bochum.pdf vom 25.09.2023
89) Schweigepflichtentbindung SAE_clean.pdf vom 25.09.2023
90) Schweigepflichtentbindung Visiten_Bochum.pdf vom 25.09.2023
91) Schweigepflichtentbindung Visiten_clean.pdf vom 25.09.2023
92) Sicherheitsfragebogen Motoriktest_Bochum.pdf vom 25.09.2023
93) Sicherheitsfragebogen Motoriktest_clean.pdf vom 25.09.2023
94) Strategie Risikominimierung SOP.pdf vom 25.09.2023
95) Unterschrift Ethikantrag MOVE PCD_26092023.pdf vom 26.09.2023
96) Unterschriftenseite Ethikantrag MOVE PCD.pdf vom 25.09.2023
97) vivofit_4_OM_DE-DE.pdf vom 25.09.2023
98) ZB_01KG2308_230627.pdf vom 25.09.2023
99) ZB_01KG2308_230627.pdf vom 26.09.2023
100) Zertifikat Probandenversicherung.pdf vom 13.11.2023

RUHR UNIVERSITÄT BOCHUM RUB

Ethics Committee of the RUB Faculty of Medicine
Gesundheitscampus 33, 44801 Bochum

St. Josef Hospital
University Clinic for Pediatrics and Adolescent
Medicine Prof. Dr. med. Thomas Lücke
Alexandrinenstr. 5
44791 Bochum

Ethics Committee
of the Faculty of Medicine

Phone: 0234/7981 6555

Fax: 0234/7981 6556

Health Campus 33, 44801 Bochum Email:

ethik@rub.de

www.ruhr-uni-bochum.de/ethik

44801 Bochum, 04.12.2023

Application dated 25.09.2023 **received in full** on 26.09.2023

Registration No.:	**23-7938-other research first (Please always specify in correspondence!)**
Topic:	Move-PCD A multicenter randomized controlled longitudinal study of the impact of a six-month individualized and supervised activity program on quality of life in children, adolescents and adults with primary ciliary dyskinesia (PCD)
Investigator:	Dr. med. Anne Schlegtendal, Prof. Dr. med. T. Lücke
Department:<br>Medicine Clinic:	University Clinic for Pediatrics and Adolescent<br>St. Josef-Hospital<br>Alexandrinenstraße 5, 44791 Bochum

Dear Professor Lücke, dear Professor Brinkmann,

The Ethics Committee of the Medical Faculty of the Ruhr University Bochum has approved the above-mentioned study in its
340th meeting on 10.10.2023. The additional information/modifications requested were received on 13.11.2023; the documents were discussed again outside of a meeting date.

On the basis of the documents submitted, the Ethics Committee has, with regard to the proposed research project in t h e context of human research
no concerns **[XX]**

The Ethics Committee points out that the medical and legal responsibility remains with the respective study physicians. All changes to the trial protocol must be submitted to the Ethics Committee and may only be implemented after the approval of the Ethics Committee.

If the study does not begin within the period specified in the application, the Ethics Committee will request notification.

All serious or unexpected adverse events that occur during the study and could affect the safety of the study participants or the conduct of the study must be reported immediately to the voting ethics committee.

The Ethics Committee expressly points out that data protection aspects of research projects are generally only examined cursorily by the Ethics Committee. This

Vote / this evaluation replaces therefore not the consultation of the responsible data protection officer.

The Ethics Committee of the Medical Faculty of the Ruhr University Bochum works in accordance with national legal regulations and the ICH-GCP guidelines.

The completion/termination of the study must be communicated and a final report submitted.
The Commission recommends registering the study with the "German Register of Clinical Trials" (www.drks.de).

For subsequent publications, the registration number and the date of the vote must be stated.

Yours sincerely, On behalf of

Christine Uphues-Schnell
Managing Physician and Member of the Ethics Committee of the Medical Faculty of the RUBochum

The consultation was based on the following documents:

1) AE Sheet_Bochum.pdf from 25.09.2023
2) AE Sheet_clean.pdf from 25.09.2023
3) Medical history form_Bochum.pdf from 25.09.2023
4) Medical history sheet_clean.pdf from 25.09.2023
5) Offer test person insurance 2023-09-08.pdf from 25.09.2023
6) Insurance offer for insured persons.pdf from 13.11.2023
7) Cover letter ethics application MOVE PCD.pdf from 25.09.2023
8) Cover letter to the ethics committee MOVE PCD_Nachforderung_signed.pdf from 13.11.2023
9) Cover letter MOVE PCD_26092023.pdf from 26.09.2023
10) Information for parents Move PCD_V1.1_Bochum.pdf from 13.11.2023
11) Clarification for parents Move PCD_V1.1_clean.pdf from 13.11.2023
12) Clarification for parents Move PCD_V1_Bochum.pdf from 25.09.2023
13) Information for parents Move PCD_V1_clean.pdf from 25.09.2023
14) Education adults Move PCD_V1.1_Bochum.pdf from 13.11.2023
15) Clarification Adults Move PCD_V1.1_clean.pdf from 13.11.2023
16) Education adults Move PCD_V1_Bochum.pdf from 25.09.2023
17) Education adults Move PCD_V1_clean.pdf from 25.09.2023
18) Education for young people Move PCD_V1.1_Bochum.pdf from 13.11.2023
19) Clarification for young people Move PCD_V1.1_clean.pdf from 13.11.2023
20) Education for young people Move PCD_V1_Bochum.pdf from 25.09.2023
21) Education for young people Move PCD_V1_clean.pdf from 25.09.2023
22) Education for children Move PCD_V1.1_Bochum.pdf from 13.11.2023
23) Clarification Children Move PCD_V1.1_clean.pdf from 13.11.2023
24) Clarification Children Move PCD_V1_Bochum.pdf from 25.09.2023
25) Clarification Children Move PCD_V1_clean.pdf from 25.09.2023
26) Concomitant medication Move PCD.pdf from 25.09.2023
27) Berichterstatter_HK_Nr 23-7938 .pdf from 09.10.2023
28) Checklist telephone appointments_Bochum.pdf from 25.09.2023

29) Checklist telephone appointments_clean.pdf from 25.09.2023
30) CRF_Follow-up_visits_Bochum.pdf from 25.09.2023
31) CRF_Folgevisite_clean.pdf from 25.09.2023
32) CRF_VO Screening_Bochum.pdf from 25.09.2023
33) CRF_VO Screening_clean.pdf from 25.09.2023
34) Data protection concept_10112023_Bochum.pdf from 13.11.2023
35) Data protection concept_10112023_clean.pdf from 13.11.2023
36) Data protection concept_25092023.pdf from 25.09.2023
37) PRIVACY POLICY FOR GARMIN CONNECT.pdf from 13.11.2023
38) PRIVACY POLICY FOR GARMIN JUNIOR.pdf from 13.11.2023
39) DATA PROTECTION POLICY GARMIN.pdf from 13.11.2023
40) Deactivation Google Analytics.pdf from 13.11.2023
41) Inclusion and exclusion criteria_ Bochum.pdf from 25.09.2023
42) Inclusion and exclusion criteria_clean.pdf from 25.09.2023
43) Consent to contact for Homepage_Bochum.pdf from 25.09.2023
44) Consent Activitiy Tracker_Bochum.pdf from 25.09.2023
45) Consent Activitiy Tracker_Bochum_V1.1.pdf from 13.11.2023
46) Consent Activity Tracker_clean.pdf from 25.09.2023
47) Consent Activity Tracker_clean_V1.1.pdf from 13.11.2023
48) Consent for parents Move PCD_V1.1_Bochum.pdf from 13.11.2023
49) Parental consent Move PCD_V1.1_clean.pdf dated 13.11.2023
50) Consent for parents Move PCD_V1_Bochum.pdf from 25.09.2023
51) Parental consent Move PCD_V1_clean.pdf dated 25.09.2023
52) Consent for adults Move PCD_V1.1_Bochum.pdf from 13.11.2023
53) Consent for adults Move PCD_V1.1_clean.pdf dated 13.11.2023
54) Consent for adults Move PCD_V1_Bochum.pdf from 25.09.2023
55) Consent for adults Move PCD_V1_clean.pdf dated 25.09.2023
56) Consent for young people MovePCD_Bochum.pdf from 25.09.2023
57) Consent for young people MovePCD_clean.pdf from 25.09.2023
58) Consent for young people MovePCD_V1.1_Bochum.pdf from 13.11.2023
59) Consent for young people MovePCD_V1.1_clean.pdf dated 13.11.2023
60) Consent for children Move PCD_V1_Bochum.pdf from 25.09.2023
61) Consent children Move PCD_V1_clean.pdf from 25.09.2023
62) Declaration on the dissertation.pdf from 25.09.2023
63) Exacerbation score PCD_ Bochum.pdf from 25.09.2023
64) Exacerbation score PCD_clean.pdf from 25.09.2023
65) Fitrockr - Data Protection - for Projects.pdf from 13.11.2023
66) Flyer MOVE PCD.pdf from 25.09.2023
67) Questionnaire activity and training wishes_Bochum.pdf from 25.09.2023
68) Questionnaire activity and training wishes_clean.pdf from 25.09.2023
69) German Adolescent QOL-PCD V2_ official version.pdf from 25.09.2023
70) German Adult QOL-PCD V2_official version.pdf from 25.09.2023
71) YOUR DATA PROTECTION RIGHTS GARMIN.pdf from 13.11.2023
72) Information Controlgroup_Bochum.pdf from 25.09.2023
73) Information control_group_clean.pdf from 25.09.2023
74) Brief description.pdf from 25.09.2023
75) Motoriktest_Bochum.pdf from 25.09.2023
76) Motorikest_clean.pdf from 25.09.2023
77) MovePCD - Application form.pdf from 25.09.2023
78) MovePCD - Application form_26092023.pdf from 26.09.2023
79) Move_PCD Protocol_final_signed.pdf from 25.09.2023
80) Move_PCD Protocol_final_signed_V1.1.pdf from 13.11.2023
81) Test person insurance policy.pdf from 13.11.2023

82) Test person information Activity restriction_Bochum.pdf from
25.09.2023
83) Subject information activity restriction_clean.pdf from 25.09.2023
84) QOL-PCD Child GERMAN_V2_official_version.pdf from 25.09.2023
85) QOL-PCD_Parent_Proxy_German V2_official_version.pdf from 25.09.2023
86) SAE Sheet_Bochum.pdf from 25.09.2023
87) SAE Sheet_clean.pdf from 25.09.2023
88) Release from confidentiality SAE_Bochum.pdf from 25.09.2023
89) Release from confidentiality SAE_clean.pdf from 25.09.2023
90) Release from confidentiality Visits_Bochum.pdf from 25.09.2023
91) Release from confidentiality Visits_clean.pdf from 25.09.2023
92) Safety questionnaire motor skills test_Bochum.pdf from 25.09.2023
93) Safety questionnaire Motorikest_clean.pdf from 25.09.2023
94) Risk minimization strategy SOP.pdf from 25.09.2023
95) Signature of ethics application MOVE PCD_26092023.pdf dated 26.09.2023
96) Signature page ethics application MOVE PCD.pdf from 25.09.2023
97) vivofit_4_OM_EN-DE.pdf from 25.09.2023
98) ZB_01KG2308_230627.pdf from 25.09.2023
99) ZB_01KG2308_230627.pdf from 26.09.2023
100) Certificate test person insurance.pdf from 13.11.2023
