# Supplementary material for: Move-PCD—a multi-center longitudinal randomized controlled superiority trial on the effect of a 6-month individualized supported physical activity (PA) program on quality of life (QoL) in children, adolescents, and adults with primary ciliary dyskinesia
Source: Trials. 2024 Aug 15;25:539. doi: 10.1186/s13063-024-08379-0 (PMC11328395; doi:10.1186/s13063-024-08379-0)
Supplement: Supplementary file 4 — Supplementary Material 4 [file 13063_2024_8379_MOESM4_ESM.pdf]

RUHR UNIVERSITÄT BOCHUM RUB

Here please find the logo of the insert own center

Study- ID_______________

**Move-PCD**

**A multicenter randomized controlled longitudinal study of the effect of a six-month individualized and supervised activity program on quality of life in children, Adolescents and adults with primary ciliary dyskinesia (PCD)**

# Information for adults

Version 1.1 09.11.2023

Dear study participant,

as a PCD patient, we have invited you to take part in a scientific study on the effect of physical activity on quality of life.

**Participation in the study is voluntary. Take your time to think about everything. Don't hesitate to ask us questions if anything is unclear. You are of course free to decline.**

**You can terminate your participation in the study at any time without giving reasons. There are no disadvantages for your treatment.**

The study is being conducted at 7 study centers in Germany (Bochum, Lübeck, Berlin, Hanover, Münster, Wesel, Koblenz). The "Kartagener Syndrom und Primäre Ciliäre Dyskinesie" e.V. supports the implementation of this study. Even if you are not being treated at any of the 7 study centers, participation is possible, for example, as part of the patient congress in Hamburg 2024.

The Department of Medical Informatics, Biometry and Epidemiology at Ruhr University Bochum (AMIB) is also involved in the study. This department is responsible for the administration, storage and evaluation of all data. This includes all test results that we collect in this study. For example, the AMIB creates a password-protected central database for this purpose.

The study is funded by DLR e.V., Health Division, on behalf of the Federal Ministry of Education and Research (BMBF).

In this study, we want to compare a six-month individual activity program with the physical activity recommended for PCD in therapy. For this purpose, all participants will be randomly divided into two groups. One group receives an individual activity program (intervention group). The other group receives the usual recommendation to exercise (control group).

Sports scientists and trainers from the Training Institute "Prof. Dr. Baum" in Cologne created this activity program. Individual physical performance, health and personal interests will be considered. The sports scientists

work closely with the doctors at the leading study center (University Clinic for Pediatrics and Adolescent Medicine Bochum) and your doctors at the *study center*(s).

**Aim of the study**

Physical activity and sport can help with chronic lung diseases. For example, the function of the lungs improves. In addition, the progression of the disease can also be slowed down. We know this from other diseases such as cystic fibrosis (CF), bronchial asthma or COPD.

Physical activity is also recommended for PCD. However, many people with PCD are less active than healthy people of the same age. One of the reasons for this is that many patients encounter obstacles to implementing an activity program in everyday life. We address this problem and work with you to develop an activity program.

In contrast to other chronic lung diseases, there has not yet been any scientific research into the impact of physical activity on PCD. This means that no one has yet tested whether a special program for PCD is good for lung function, improves quality of life and slows down the progression of the disease. We want to change that.

Additionally we want to examine whether regular contact and support from exercise specialists can help to overcome the obstacles.

**Who is not allowed to participate in this project?**

Unfortunately, you may not take part in the project if one of the following criteria applies. If you are unsure whether you suffer from one of the diseases, please contact your doctor or us.

| | |
|---|---|
| ○ Detected genetic mutation: CCDC39/40, CCNO | ○ Lung transplantation (also planned) |
| ○ decompensated heart failure | ○ Oxygen therapy |
| ○ Cor pulmonale | ○ Permanent respiratory support (non-invasive ventilation, NIV) |
| ○ Hypertrophic cardiomyopathy | ○ Cancer currently under therapy |
| ○ High blood pressure that is not normal under drug therapy | ○ Physical inability to participate in sport or orthopedic restrictions that preclude participation in the exercise program |
| ○ Myocarditis in the last 6 months | ○ Alcohol or drug abuse |
| ○ Unstable/progressive angina pectoris (e.g. new onset of angina, increase in symptoms, need for more medication) | ○ Pregnancy |
| ○ uncontrolled cardiac arrhythmias (e.g. recurrent ventricular tachycardia, tachyarrhythmias, atrial fibrillation) | ○ The following physical complaints under stress<br>▪ Shortness of breath beyond the expected level<br>▪ Fainting<br>▪ Chest pain/thoracic tightness |

GEFÖRDERT VOM
Bundesministerium für Bildung und Forschung
DLR
TRAININGSINSTITUT Prof. Dr. Baum
leben mit PCD Kartagener Syndrom und Primäre Ciliäre Dyskinesie e.V.

Here please find the logo of the insert own center

| | |
|---|---|
| | ▪ Tendency to fall (also musculoskeletal-related) |
| ○ moderate to high-grade heart defects, especially of the left heart (high-grade or symptomatic aortic valve stenosis, high-grade mitral valve insufficiency) | |
| ○ Heart surgery (including pacemaker surgery/ ICD surgery in the last 3 months) | |
| ○ Stroke in the last 5 years | |
| ○ Marfan/Ehlers-Danlos syndrome | |
| ○ uncontrolled bronchial asthma (i.e. medication on demand more than 2 days a week or nocturnal symptoms) | |

**Benefits from the study**

The study gives you the opportunity to improve your physical fitness through regular sporting activity. This can have a positive impact on your physical, mental and social health. We expect an improvement in your quality of life and an increase in your participation in daily life. In addition, we expect a slowing of the progression of your disease with an improvement in lung function. If the supervised activity program proves to be effective, it could be firmly anchored in the therapy and rehabilitation guidelines for PCD. This would make it possible to reimburse the fees for corresponding sports courses.

**Procedure of the study**

If you decide to take part in the project and have given your written consent, you will be examined (**screening visit**). This is to ensure that you can participate in the study and the activity program without any concerns.

The screening visit can take place at your supervising study center, at the leading study center (University Clinic for Pediatrics and Adolescent Medicine Bochum) or at the Patient Congress 2024 in Hamburg.

We, the study team of the *Name Study Center*, will take the current **medical history**. We will ask you about concomitant illnesses, current complaints and your current therapy. Please continue your standard therapy as usual during the study. Please contact us if there are any changes. You will also undergo a **physical examination**, a **lung function test** (if possible with measurement of the Lung Clearance Index (**LCI**)) and an **ECG examination**. Based on the results, we will then make a final decision as to whether you can take part in the study or not. In some cases, we may also ask you to have further tests carried out. We will then include you in the study at a later date (after a maximum of 4 weeks).

GEFÖRDERT VOM
Bundesministerium für Bildung und Forschung

If you are able to participate in the study, we will ask you to complete a questionnaire **on quality of life.** You will receive a personal link with which you can complete the questionnaire directly in a secure database. We will be happy to provide you with a tablet for this purpose. The questionnaire was developed specifically for people with PCD (QoL-PCD). The developers from Canada would like to analyze these questionnaires from all participants anonymously. In this way, they want to continuously improve the questionnaires. To this end, the results will be forwarded without your personal details (i.e. without your name, date of birth or contact details). It will then no longer be possible for anyone to link the completed questionnaire to you personally.

The employees of the training institute "Prof. Dr. Baum" will carry out a standardized motor skills test with you. This test consists of 6 different tests. Each test will be explained and shown to you again before you start. We will also ask you to complete a **questionnaire** on your physical activity and training requirements. The test and the questionnaires are used to determine your current fitness level. This allows us to better tailor the activity program to you. The motor skills test may take place on a separate date. In this case, we, the study team at the *Name Study Center*, will inform you ahead. The motor function test and the other examinations of the screening visit must take place within 4 weeks.

1) Standing long jump: You stand with both toes on a starting line at shoulder width. You jump and land with both feet at the same time. The distance (in cm) up to the toe of the back leg is counted. The best of 3 attempts counts.

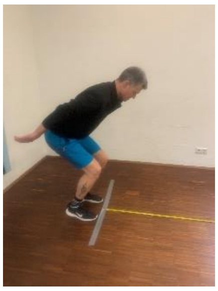

2) Push-up: Two Theraband stability trainers (ThS) are placed on top of each other (together 10 cm high). Start with your arms outstretched. Your body is stretched out and the tips of your feet are shoulder-width apart on the floor. With your body stretched out, bend your arms until your chest touches the ThS. Then straighten them again. Flexion and extension are performed without a rhythm. The number of valid attempts within 30 seconds is measured.

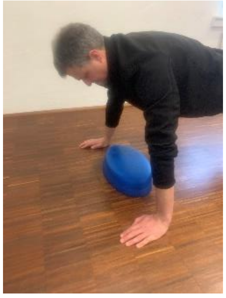

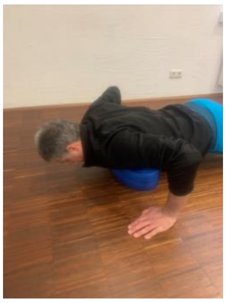

3) <u>One-legged stand:</u> Stand two meters away from a marker and focus on it with your eyes. The start is made by lifting the playing leg. The time in the one-legged stand is measured without the playing leg touching the ground or the standing leg being moved. The test is stopped after 60 seconds. Both legs are first tested with open eyes. In the event of premature termination, a second attempt is permitted for each leg. The same test procedure is then carried out with a blindfold.

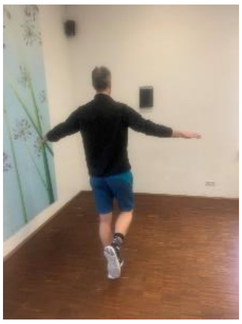

4) <u>Sit-up:</u> Lie on your back with your knees bent parallel. The head rests on two Theraband stability trainers (ThS). The feet are held in place by the person conducting the test. During the test, the upper body is raised until both hands touch the ankles. The upper body is then lowered again until the head touches the ThS. The number of complete executions within 30 seconds is counted.

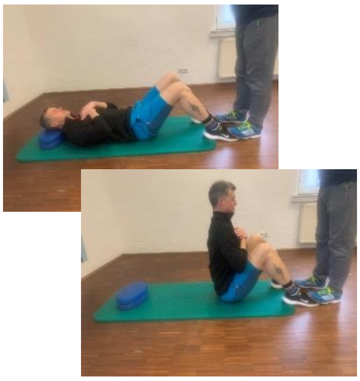

5) <u>Torso bend:</u> Stand on a box with your knees straight. Then bend your upper body forwards and downwards as far as possible. The distance (in cm) is measured at the height of the tip of the middle finger without rocking.

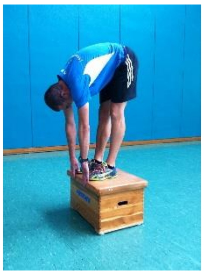

6) <u>10-minute run:</u> You run around a marked field for 10 minutes. You can set your own pace. The aim is to cover as much distance as possible. Alternating between running and walking is permitted. The distance covered is recorded (= number of laps x 65m + remaining distance in meters)

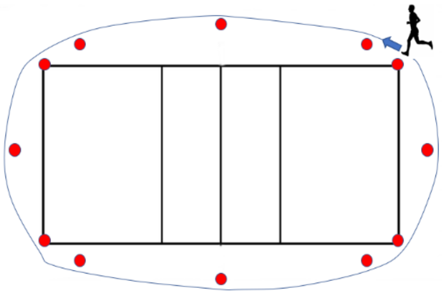

You will then receive a GARMIN **activity tracker** (Vivofit 4). The activity tracker is worn like a wristwatch. It can measure your heart rate and count your sleep time and steps. We would like to ask you to wear it day and night for the entire duration of the study - i.e. for 12 months. The data from the activity tracker can be read out with

the help of an app. We would like to ask you to install this app or to connect the activity tracker to your computer. Your data from the activity tracker and your login details will then be saved in the app. After the study or if you no longer wish to participate, you can delete the app. All data will then also be deleted from GARMIN. The data helps to better design the individual activity program. In addition, obstacles to physical activity, such as lack of sleep, are recorded. This allows us to objectively measure how active you are. We can compare the physical activity in the intervention group with the data of the control group and examine how this affects quality of life. The data is automatically transferred from the app to a central database and stored there. In addition to the written instructions, the staff of the Training Institute “Prof. Dr. Baum” will explain how to use the app and show you how to wear the activity tracker. We ask you to wear it as soon as you receive it.

Within four weeks of the screening visit, you will be randomly assigned to the control or intervention group by the AMIB. This is called randomization.

We will inform you by telephone which group you have been assigned to. If you are part of the **intervention group,** the Training Institute “Prof. Dr. Baum” will then contact you. You will receive a plan for an **individual activity program.** You will also receive appointments for **weekly meetings with** a trainer. These last 20 minutes and can take place by telephone or video call. These appointments are important. Here you can discuss any problems or obstacles with your activity program. You can adjust your activity program at any time with your trainer according to your wishes. You will also be asked about your health and well-being during the activity program. You may be asked to pause the activity program if you have health problems. The trainer will pass on any indications of health problems to the leading study center (University Clinic for Pediatrics and Adolescent Medicine Bochum). A doctor will check whether you can continue the activity program without any problems. You will be informed by telephone within a few days.

Once a week, you will receive an e-mail with a link to the online platform "taskcards". Here you will find **links to three videos per week**: the online courses "Enjoy the day", "Strengthen your body" and "Move the Rhythm". Each video lasts 45 minutes. The courses are built on each other. You can decide for yourself when and if you want to do the courses. The videos are created especially for this study. Please do not pass on the links. Especially not to participants from the control group. The control group will have access to all the videos after the end of the study and can then also benefit. If you take part in a course, please comment on it briefly under "taskcards" (e.g. "good", "too strenuous", etc.). This helps us to improve the courses and to get an overview of which courses are suitable for people with PCD.

During the study, it is important for us to know how your health is and how physical activity affects your quality of life. Of course, we also want to know whether and which problems can arise as a result of physical activity. We would therefore like to examine you at regular intervals. We would also like to use the visits to record your movement behavior in everyday life and compare it with the measurements taken at the beginning. We can of course send you the results after the evaluation.

We will therefore invite you to **study visits** 3, 6 and 12 months after you have been assigned to a group. You are welcome to combine these with your outpatient appointment with us at the *Name Study Center.*

A current **medical history and physical examination will be** carried out again during the study visits. We will check your **lung function** and repeat the **motor function test**. We will ask you to complete the **quality of life questionnaire** again**.** Also important to us will be any health problems, unplanned visits to the doctor and hospital stays, so-called adverse events.

Please bring the "activity tracker" with you to the visits. Please return it to us at the last visit.

Due to the high logistical effort involved, we can only offer motor skills tests on certain dates. It is therefore possible that you will receive a separate appointment for the motor skills test. The motor skills tests take place in gyms close to the study center. You will receive the exact address with the appointment.

If you have decided to participate during the patient congress or are not being cared for at a study center, the study visits can take place at the leading study center (University Clinic for Pediatrics and Adolescent Medicine Bochum). Alternatively, it is possible for us to conduct parts of the study visit by telephone. In this case, you must have a physical examination and lung function measurement carried out by your supervising PCD doctor. You should then send the examination results to the study team at the University Clinic for Pediatrics and Adolescent Medicine Bochum. In this case, we ask you to sign a corresponding confidentiality release. The motor function tests will be organized close to home. You will receive the exact address with the appointment.

There is a budget for travel expenses as part of the study. You can submit these to the Ruhr-Universität Bochum with the corresponding receipt. You will receive the form for submission from us during the visit.

**How much time does it take?**

The time frame for the activity program is not fixed. You can decide for yourself. Your personal activity program includes physical and sporting activities that suit your inclination. The online courses last 45 minutes each. It is possible to take part in three courses per week. During the program, you will be contacted once a week for 20 minutes by telephone or video call to discuss problems (motivation and barriers, changes to the exercise program) and questions (e.g. exercises, implementation, etc.).

The four study visits (screening visit and those after 3, 6 and 12 months) do not take significantly longer than a regular outpatient appointment and can be combined with it. Please allow approximately 60 minutes per visit. We would like to ask you to allow half a day for each of the motor function tests. The test itself only takes about

1.5 hours. However, due to the logistical effort involved, several participants will be tested on one day, which may result in waiting times.

**Table 1: Investigations within the scope of the study with time expenditure**.

Some of the examinations are part of the normal outpatient appointments and therefore do not require any additional time.

| | V0 | V1 | V2 | V3 | V4 |
|---|---|---|---|---|---|
| | Screening visit and basic examination | Randomization at the beginning of the intervention period | Follow-up examination | End of the intervention period | End of the monitoring phase |
| | - 5 weeks until day 1 | Day 1 | 3±1 months | 6±1 months | 12±1 months |
| Checking the inclusion and exclusion criteria (5-10 minutes) | X | | | | |
| Educational talk (15 minutes) | X | | | | |
| Medical history including number of exacerbations in the last year, current medication, concomitant illness, etc. (20 minutes) | X | | X | X | X |
| Physical examination (10 minutes) | x | | X | X | x |
| ECG (10 minutes) | x | | | | |
| Pulmonary function test (20 minutes) | x | | X | X | x |
| Motor skills test (45-60 minutes) | x | | X | X | x |
| Questionnaire on sporting activity and training preferences (5 minutes) | X | | | | |
| Handing out the activity tracker and introduction to its use (15 minutes) | X | | | | |
| Quality of life questionnaire (QoL) (15 minutes) | X | | X | X | X |

**Are there any risks?**

Possible risks may occur during the motor function test, physical activity, lung function and training. However, we try to keep the risks as low as possible.

During the stress test, headaches, dizziness, nausea and increased coughing may occur. If these symptoms occur, the stress test is stopped immediately.

Problems can occur when carrying out the activity program that are not necessarily related to PCD. Risks during sport can include muscle injuries, ligament injuries in the ankle joint, fractures, dislocation of joints (e.g. ankle joint).

Risks associated with exercise that are known to occur with other chronic lung diseases such as cystic fibrosis include: Shortness of breath, coughing on exertion, dizziness, drop in blood sugar, pain in the joints, fractures in the event of a fall.

To reduce these risks, we will talk to you about them before you start the exercise program and give you recommendations on how to avoid them.

Please do not exercise if you have the following symptoms:

- Fever or other infections that are accompanied by a feeling of illness
- Acute pulmonary exacerbation
- Severe joint, back or headaches during or immediately after sport
- Chest pain or pain in the left arm
- Dizziness/fainting
- Shortness of breath (beyond the expected level) or hyperventilation
- Hypoglycemia/ hypoglycemia
- Persistent palpitations or palpitations after exercise
- Pacemaker/ICD depending on the sport

**Why do we need a control group?**

The "Move PCD" project aims to investigate the effects of regular physical activity on physical performance, lung function and other parameters. These effects can only be determined by comparing participants in the activity program with those who do not take part in the activity program. In this way, we can determine whether the positive effects are actually present. For this reason, we need a control group.

**What else do I have to do as a participant in the control group?**
We would like to ask you to take part in the **study visits** and to wear the **activity tracker for the** entire duration of the study. This small device measures your movements in everyday life, your heart rate and the duration of your sleep. Being in the control group <u>**does not**</u> mean that you are no longer allowed to exercise. In fact, it is important that you **continue to be physically active on a regular basis** as before the study, as recommended by your doctor.

**Is there insurance?**

Special volunteer insurance was taken out for all participants. This covers all examinations at the *name study center* as well as physical activity during the study and exists for

Ecclesia Versicherungsdienst GmbH

Ecclesiastrasse 1 - 4, 32758 Detmold

Phone +49 5231 603-0, Fax +49 5231 603-197

info@ecclesia.de

Order no. NEV071767A

In addition, separate travel insurance was taken out for the journey to and from the examination location.

**SV SparkassenVersicherung Holding AG**
Löwentorstraße 65
70376 Stuttgart
Phone 0711 898-100
Fax 0711 898-109

Order no. 50 103 108/495

One accompanying person is also insured by Ecclesia Versicherungsdienst GmbH (insurance no. ***0032369003 - 2023/1***).

You will receive copies of the insurance policy at the beginning of the study. If you suspect that an insured event has occurred, please contact your supervising study center, the leading study center in Bochum or the insurance company immediately.

**How will the collected data in this study be used?**

All data collected is subject to medical confidentiality and are treated as such. A data protection concept is available. This can be viewed on request. All data collected is stored on paper or electronically. They are evaluated without mentioning names and other personal data such as date of birth or address (pseudonymized). Name and date of birth are replaced by a letter/number code (so-called subject identification number). This makes it impossible or difficult to draw conclusions about the identity (§ 3 BDSG). The pseudonymization takes place after your personal data has been entered into a separate and secure central database ("contact database") by the AMIB. Only selected groups of people will have access to this contact database containing the

contact details of all participants. The lead study center in Bochum and the training institute Prof. Dr. Baum will have access to the contact details of all participants for the duration of the study in order to coordinate appointments, to carry out the intervention and after examination to release the intervention in the event of health problems. The local study centers only know the subject identification number of their study participants in order to be able to enter the data of the participants from their center into the study database.

In the study database, all data collected as part of the study will only be stored with the pseudonymization code. Access to the study database is also released separately. The study team at the *Name Study Center* may only restore the link between personal data and subject identification in precisely defined cases, e.g. if a participant falls ill.

For the scientific evaluation, presentation and publication of the data and study results, the participants' data will only be used in pseudonymized form.

In all surveys and studies, the medical-scientific research data is never linked to identity data, but only to the subject identification. The identity data is stored for 10 years after the end of the study. By deleting the identity data, the data is anonymized, i.e. it can never again be assigned to a person. The anonymized data will be deleted at the earliest 10 years after the last scientific publication.

**Information on data protection and use of the GARMIN Connect app in connection with the "activity trackers" as part of the study**

The GARMIN Connect app must be installed to synchronize the activity tracker data. You must create an account to record the activity data. This requires an e-mail address and name (including nickname, for example). Please do not use 2-factor authentication, in which case no phone number will be collected. In addition, you must enter your date of birth, gender, height and weight as well as the times you get up and go to bed in your account so that your activity and energy consumption can be measured.

The data protection guidelines of the GARMIN Connect app apply. You will receive these and the GARMIN data protection guidelines from us. Please read them carefully.

You are using this app as part of a study, so please note the following points:

- GARMIN is an international company and processes data not only in Europe, but also in the USA and Australia.
- GARMIN also processes data in its own interest, e.g. to improve products or detect fraud. You generally consent to this by using the app. You will not be informed or asked for your consent each time GARMIN processes data.
- As part of this study, please withdraw your consent to marketing information, the use of insights and email notifications.

- Do not link any third-party apps, especially social networks such as Facebook, with the GARMIN Connect app and do not connect with other users of the GARMIN Connect app.
- Set your profile to anonymous.
- Do not take part in challenges during the study period and please do not use any location services (weather, etc.). Also, do not use real-time tracking (Assistance Plus services) with an emergency contact.
- The GARMIN Connect app uses Google Analytics. This is prohibited in the context of studies. You must therefore withdraw your consent to the use of data. You can do this by using the browser add-on to deactivate Google Analytics.
- The data will be stored by GARMIN for as long as your account is active. You can only delete your data from GARMIN yourself. You can do this in your account. At the end of the study, please delete your account completely.

We buy the "activity tracker" for you. The topic of payment processing and shipping in the data protection guidelines of GARMIN therefore has no relevance for you.

The data from the app is automatically transferred to a central database (Fitrockr Health Solutions) and stored there pseudonymized, i.e. only with your letter/number code. Fitrockr Health Solutions is a company based in Germany. Data management takes place exclusively in Germany. The data in the central database is managed by the leading study center of the University Children's Hospital Bochum and the AMIB and, like all other data, can be deleted at your request - if possible (see below).

**Your rights with regard to data protection**

Consent to the processing of your data is voluntary. You can revoke your consent at any time without giving reasons and without any disadvantages for you. No more data will then be collected. If you withdraw your participation in this study, your data will be deleted as soon as this is possible. If retention periods prevent deletion, your data will no longer be used and will be deleted immediately after expiry. This does not affect the lawfulness of the processing carried out on the basis of the consent until revocation. You have the right to receive information about the data, also in the form of a copy free of charge. In addition, you can request the rectification, blocking, restriction of processing or erasure and, if necessary, the transfer of the data. This means that you can have your data made available to you in a transferable form or have it sent to a location specified by you. This also applies if you wish the findings to be sent to your attending physician at a later date.

**The person responsible for data processing in the project is the project management**:

Prof. Dr. med. Folke Brinkmann
University Clinic for Pediatrics and Adolescent Medicine at St. Josef Hospital
Catholic Hospital Bochum
Department of Pediatric Pneumology
Alexandrinenstr. 5

Here please find the logo of the insert own center

44791 Bochum

**The person responsible for data processing at the Study Center is**:

Local study management

**The company data protection officer of the Ruhr-Universität Bochum is:**
Dr. Kai-Uwe Loser
Ruhr University Bochum
Data Protection Officer
Wasserstr. 221
44799 Bochum

**The data protection officer of the** hospital/study center **is**:

xxxx

**Responsible data protection supervisory authority**:

xxxx

**Data protection supervisory authority of the federal state of xxx**:

Xxxxx

**Who do I contact if I have any questions?**

The study physicians and investigators will be happy to answer any further questions you may have. General questions, including questions concerning your rights as a participant, will also be answered by the following persons:

Study physician:in:

Local

Study office of the hospital:

local

Here please find the logo of the insert own center

# Appendix

**Leading Study Center:**

University Clinic for Pediatrics and Adolescent Medicine at St. Josef Hospital

Catholic Hospital Bochum

Department of Pediatric Pneumology

Alexandrinenstr. 5

44791 Bochum

**Other participating study centers:**

University Medical Center Schleswig-Holstein, Lübeck Campus

Section for Pediatric Pneumology and Allergology

Ratzeburger Allee 160, House A

23538 Lübeck

Charité University Medicine Berlin

Clinic for Pediatrics with a focus on Pneumology, Immunology and Intensive Care Medicine

Campus Virchow Clinic

Augustenburger Platz 1

13353 Berlin

Hanover Medical School

Clinic for Pediatric Pneumology, Allergology and Neonatology

and

Clinic for Pneumology and Infectiology

Carl-Neuberg-Str.1

30625 Hanover

Clinic for Pediatrics and Adolescent Medicine at Münster University Hospital

Here please find the logo of the insert own center

Pediatric pneumology

Albert-Schweitzer-Campus 1, Building A1

48149 Münster

Marienhospital Wesel gGmbH

Pediatrics and adolescent medicine

Pastor-Janßen-Str. 8-38

46483 Wesel

Gemeinschaftsklinikum Mittelrhein

Clinic for Pediatrics and Adolescent Medicine Kemperhof

Koblenzer Strasse 115-155

56073 Koblenz

**Statistical management:**

Prof. Dr. rer. nat. Nina Timmesfeld

Ruhr University Bochum

Department of Medical Informatics, Biometry and Epidemiology (AMIB)

University Road 105

4789 Bochum

**Cooperation partner:**

Training Institute Prof. Dr. Baum

Wilhelm-Schlombs-Allee 1

50858 Cologne

Kartagener Syndrome and Primary Ciliary Dyskinesia e. V.

GEFÖRDERT VOM Bundesministerium für Bildung und Forschung

Here please find the logo of the insert own center

Königswarter Str. 5

65366 Geisenheim

**Sponsor:**

Ruhr University Bochum

University Road 150

44801 Bochum

**Funding by**: DLR Project Management Agency, Health Division

**Commissioned by**: Federal Ministry of Education and Research

Here please find the logo of the insert own center

Study-ID________________

**Move-PCD**
**A multicenter randomized controlled longitudinal study of the effect of a six-month individualized and supervised activity program on quality of life in children, adolescents and adults with primary ciliary dyskinesia (PCD)**

## Information for parents/guardians

Version 1.1 09.11.2023

Dear parents/guardians,

your child has PCD and is 7 years or older. We have therefore asked your child to take part in a scientific study on the effect of physical activity on quality of life.

**Participation in the study is voluntary. Take your time to think about everything together with your child. Don't hesitate to ask us questions if anything is unclear. You and your child can of course refuse.**

**Participation in the study can be terminated by you and your child at any time without giving reasons. There are no disadvantages for you or your child.**

The study is being conducted at 7 study centers in Germany (Bochum, Lübeck, Berlin, Hanover, Münster, Wesel, Koblenz). The "Kartagener Syndrom und Primäre Ciliäre Dyskinesie" e.V. supports the implementation of this study. Even if your child is not being treated at any of the 7 study centers, participation is still possible, for example, as part of the patient congress in Hamburg 2024.

The Department of Medical Informatics, Biometry and Epidemiology at Ruhr University Bochum (AMIB) is also involved in the study. This department is responsible for the administration, storage and evaluation of all data. This includes all test results, we will collect in this study. For example, the AMIB creates a password-protected central database for this purpose.

The study is funded by DLR e.V., Health Division, on behalf of the Federal Ministry of Education and Research (BMBF).

In this study, we want to compare a six-month individual activity program with the physical activity recommended for PCD in therapy. For this purpose, all participants will be randomly divided into two groups. One group receives an individual activity program (intervention group). The other group receives the usual recommendation to exercise (control group).

GEFÖRDERT VOM
Bundesministerium für Bildung und Forschung
DLR
TRAININGSINSTITUT Prof. Dr. Baum
leben mit PCD Kartogener Syndrom und Primäre Ciliäre Dyskinesie e.V.

Here please find the logo of the
insert own center

Sports scientists and trainers from the Training Institute “Prof. Dr. Baum” in Cologne created this activity program. Individual physical performance, health and personal interests will be considered. The sports scientists work closely with the doctors at the leading study center (University Clinic for Pediatrics and Adolescent Medicine Bochum) and your doctors at the *study center*(s).

**Aim of the study**

Physical activity and sport can help with chronic lung diseases. For example, the function of the lungs improves. In addition, the progression of the disease can also be slowed down. We know this from other diseases such as cystic fibrosis (CF), bronchial asthma or COPD.

Physical activity is also recommended for PCD. However, many people with PCD are less active than healthy people of the same age. One of the reasons for this is that many patients encounter obstacles to implementing an activity program in everyday life. We address this problem and work with you to develop an activity program.

In contrast to other chronic lung diseases, there has not yet been any scientific research into the impact of physical activity on PCD. This means that no one has yet tested whether a special program for PCD is good for lung function, improves quality of life and slows down the progression of the disease. We want to change that.

Additionally we want to examine whether regular contact and support from exercise specialists can help to overcome the obstacles.

**Who is not allowed to participate in this project?**

Unfortunately, your child may not take part in the project if one of the following criteria applies. If you are unsure whether your child suffers from one of the diseases, please contact the doctor treating your child or us.

| | |
|---|---|
| ○ Detected genetic mutation: CCDC39/40, CCNO | ○ Lung transplantation (also planned) |
| ○ decompensated heart failure | ○ Oxygen therapy |
| ○ Cor pulmonale | ○ Permanent respiratory support (non-invasive ventilation, NIV) |
| ○ Hypertrophic cardiomyopathy | ○ Cancer currently under therapy |
| ○ High blood pressure that is not normal under drug therapy | ○ Physical inability to participate in sport or orthopedic restrictions that preclude participation in the exercise program |
| ○ Myocarditis in the last 6 months | ○ Alcohol or drug abuse |
| ○ Unstable/progressive angina pectoris (e.g. new onset of angina, increase in symptoms, need for more medication) | ○ Pregnancy |
| ○ uncontrolled cardiac arrhythmias (e.g. recurrent ventricular tachycardia, tachyarrhythmias, atrial fibrillation) | ○ The following physical complaints under stress<br>▪ shortness of breath beyond the expected level |

GEFÖRDERT VOM
Bundesministerium für Bildung und Forschung

| | |
|---|---|
| | ▪ fainting<br>▪ chest pain/thoracic tightness<br>▪ tendency to fall (also musculoskeletal-related) |
| ○ moderate to high-grade heart defects, especially of the left heart (high-grade or symptomatic aortic valve stenosis, high-grade mitral valve insufficiency) | |
| ○ Heart surgery (including pacemaker surgery/ ICD surgery in the last 3 months) | |
| ○ Stroke in the last 5 years | |
| ○ Marfan/Ehlers-Danlos syndrome | |
| ○ uncontrolled bronchial asthma (i.e. medication on demand more than 2 days a week or nocturnal symptoms) | |

**Benefits from the study**

The study gives your child the opportunity to improve their physical fitness through regular sporting activity. This can have a positive impact on your child's physical, mental and social health. We expect an improvement in the quality of life and an increase in participation in daily life. In addition, we expect a slowing of the progression of the disease with an improvement in lung function. If the supervised activity program proves to be effective, it could be firmly anchored in the therapy and rehabilitation guidelines for PCD. This would make it possible to reimburse the fees for corresponding sports courses.

**Procedure of the study**

If you and your child decide to take part in the project and have given your written consent, examinations will be carried out (**screening visit**). This is to ensure that your child can participate in the study and the activity program without any concerns.

The screening visit can take place at the supervising study center, at the leading study center (University Clinic for Pediatrics and Adolescent Medicine Bochum) or at the Patient Congress 2024 in Hamburg.

We, the study team at the *Name Study Center*, will take the current **medical history**. We will ask you and your child about concomitant illnesses, current complaints and current therapy. Please continue your child's standard therapy as usual during the study. Please contact us if there are any changes. We will also carry out a **physical examination**, a **lung function test** (if possible with measurement of the Lung Clearance Index (**LCI**)) and an **ECG examination**. Based on the results, we will then make a final decision as to whether your child can take part in

the study or not. In some cases, we may also ask you and your child to have further tests carried out. We will then include your child in the study at a later date (max. after 4 weeks).

If your child is able to take part in the study, we will ask you to complete a questionnaire **on quality of life**. You will receive a personal link with which you can complete the questionnaire directly in a secure database. We will be happy to provide you with a tablet for this purpose. The questionnaire was specifically developed for people with PCD (QoL-PCD). The developers from Canada would like to analyze these questionnaires from all participants anonymously. In this way, they want to continuously improve the questionnaires. The results will be forwarded without the personal data of your child or yourself (i.e. without name, date of birth or contact details). It will then no longer be possible for anyone to assign the completed questionnaire to you or your child.

The staff at the Training Institute “Prof. Dr. Baum” will carry out a standardized motor skills test with your child. This test consists of 6 different tests. Each test will be explained and shown again before the start. We will also ask your child to complete a **questionnaire** about his/her physical activity and training requirements. The test and the questionnaires are used to determine the current fitness level. This allows us to better tailor the activity program to your child. The motor skills test may take place on a separate date. In this case, we, the study team at the *Name Study Center*, will inform you ahead. The motor skills test and the other examinations of the screening visit must take place within 4 weeks.

1) Standing long jump: Your child stands with both toes on a starting line at shoulder width. They jump and land with both feet simultaneously. The distance (in cm) up to the toe of the back foot is counted. The best out of 3 attempts counts.

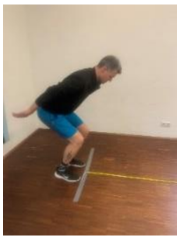

2) Push-up: Two Theraband stability trainers (ThS) are placed on top of each other (together 10 cm high). Your child starts with their arms outstretched. The body is stretched out and the tips of the feet are shoulder-width apart on the floor. With the body stretched out, the arms are bent until the chest touches the ThS then straighten again. Flexion and extension are performed without a rhythm. The number of valid attempts within 30 seconds is measured.

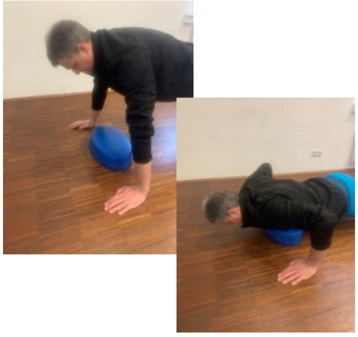

3) <u>One-legged stand:</u> Your child stands two meters away from a marker and focuses on it with their eyes. The start is made by lifting the playing leg. The time in the one-legged stand is measured without the playing leg touching the ground or the standing leg being moved. The test is stopped after 60 seconds. Both legs are first tested with open eyes. In the event of premature termination, a second attempt is permitted for each leg. The same test procedure is then carried out with a blindfold.

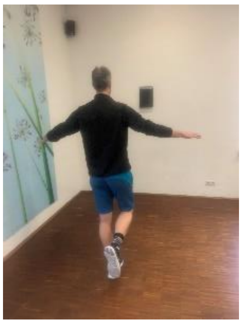

4) <u>Sit-up:</u> Your child lies on their back with their knees bent parallel. The head rests on two Theraband stability trainers (ThS). The feet are held in place by the person conducting the test. During the test, the upper body is raised until both hands touch the ankles. The upper body is then lowered again until the head touches the ThS. The number of complete executions within 30 seconds is counted.

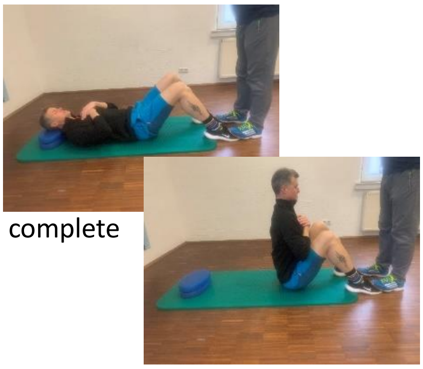

5) <u>Torso bend:</u> Your child stands on a jumping box with their knees stretched out then bends the upper body forwards and downwards as far as possible. The distance (in cm) is measured at the height of the tip of the middle finger without rocking.

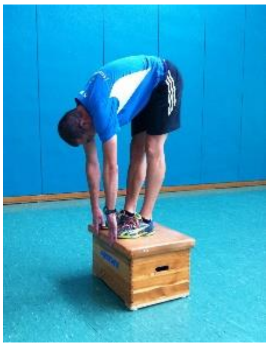

6) <u>10-minute run:</u> Your child runs around a marked field for 10 minutes. They can set their own pace. The aim is to cover as much distance as possible. Alternating between running and walking is allowed. The distance covered is recorded (= number of laps x 65m + remaining distance in meters)

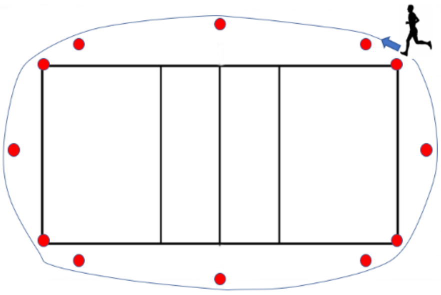

Your child will then receive a Garmin **activity tracker** (Vivofit 4). The activity tracker is worn like a wristwatch. It can measure the heart rate and count the duration of sleep and steps. We would like to ask your child to wear it day and night for the entire duration of the study - i.e. for 12 months. The data from the activity tracker can be read out with the help of an app. We would ask you to install this app or to connect the activity tracker to

your computer. Registration is only permitted from the age of 16, so we would like to ask you to create an account for your child. The app will then store the data from your child's activity tracker and your login details. After the study or if your child no longer wishes to participate, you can delete the app. All data will then also be deleted from GARMIN. The data helps to better design the individual activity program. In addition, obstacles to physical activity, such as lack of sleep, are recorded. This allows us to objectively measure how active your child is. We can compare the physical activity in the intervention group with the data of the control group and investigate how this affects quality of life. The data is automatically transferred from the app to a central database and stored there. In addition to the written instructions, the staff of the Training Institute "Prof. Dr. Baum" will explain how to use the app and show you how your child should wear the activity tracker. We ask your child to wear it immediately after receiving it.

Within four weeks of the screening visit, your child will be randomly assigned to the control or intervention group by the AMIB. This is called randomization.

We will inform you by telephone which group your child has been assigned to. If it is part of the **intervention group,** the Training Institute "Prof. Dr. Baum" will then contact you. You will receive a plan for an individual activity program. You and your child will also receive appointments for weekly meetings with a trainer. These last 20 minutes and can take place by telephone or video call. These appointments are important. Here you and your child can discuss any problems or obstacles with your activity program. The activity program can be adjusted at any time with the trainer according to your child's wishes. Your child will also be asked about their health and well-being during the activity. Your child may be asked to take a break from the activity program if they have health problems. The trainer will pass on any indications of health problems to the leading study center (University Clinic for Pediatrics and Adolescent Medicine Bochum). A doctor will check whether your child can continue the activity program without any problems. You will be informed of the decision by telephone within a few days.

Once a week, you will receive an email with a link to the online platform "taskcards". Here you and your child will find links to three videos per week: the online courses "Enjoy the day", "Strengthen your body" and "Move the Rhythm". Each video lasts 45 minutes. The courses are built on each other. Your child can decide for themselves when and whether to do the courses. The videos are created especially for this study. Please do not pass on the links to anyone else. Especially not to participants from the control group. The control group will have access to all the videos at the end of the study and can then also benefit. If your child takes part in a course, please comment on it briefly together on "taskcards" (e.g. "good", "too strenuous", etc.). This helps us to improve the courses and to get an overview of which courses are suitable for people with PCD.

During the study, it is important for us to know how your child is doing health-wise and how physical activity affects their quality of life. Of course, we also want to know whether and which problems can arise as a result of physical activity. We would therefore like to examine your child at regular intervals. We would also like to use the visits to record your child's movement behavior in everyday life and compare it with the measurements taken at the beginning. We can of course send you the results after the evaluation.

3, 6 and 12 months after your child has been assigned to a group, we will therefore invite them to **study visits.** You and your child are welcome to combine these with your outpatient appointment with us at the *Name Study Center.*

During the study visits, a **current medical history and physical examination** are carried out again. We check the **lung function** and repeat the **motor function test**. We will also ask you and your child to complete the **quality of life questionnaire** again. You and your child will also be asked about any health problems, unplanned visits to the doctor and hospital stays, so-called **adverse events.** Please bring the "**activity tracker**" with you to the visits. You and your child should return it to us at the last visit.

Due to the high logistical effort involved, we can only offer the motor skills tests on certain dates. It is therefore possible for you and your child to receive a separate appointment for the motor skills test. The motor skills tests take place in gyms close to the study center. You will receive the exact address with the appointment.

If you and your child have decided to participate during the patient congress or are not cared for at a study center, the study visits can take place at the leading study center (University Clinic for Pediatrics and Adolescent Medicine Bochum). Alternatively, it is possible for us to conduct parts of the study visit by telephone. In this case, you must have a physical examination and lung function measurement performed by your child's supervising PCD doctor. You should then send the examination results to the study team at the University Clinic for Pediatrics and Adolescent Medicine Bochum. In this case, we ask you to sign a corresponding confidentiality release. The motor function tests will be organized close to home. You will receive the exact address with the appointment.

There is a budget for travel expenses as part of the study. You can submit these to the Ruhr-Universität Bochum with the corresponding receipt. You will receive the form for submission from us during the visit.

**How much time does it take?**

The time frame for the activity program is not fixed. Your child can decide for themselves. The personal activity program includes physical and sporting activities that suit their inclinations. The online courses last 45 minutes each. It is possible to take part in three courses per week. During the program, your child will be contacted by (video) telephone once a week for 20 minutes (together with you, depending on age) to discuss problems (motivation and barriers, changes to the exercise program) and questions (e.g. exercises, implementation, etc.).

The four study visits (screening visit and those after 3, 6 and 12 months) do not take significantly longer than a regular outpatient appointment and can be combined with it. You and your child should allow approximately 60 minutes per visit. We would like to ask you to allow half a day for each of the motor skills tests. The test itself only takes about 1.5 hours. However, due to the logistical effort involved, several participants will be tested on one day, which may result in waiting times.

**Table 1: Investigations within the scope of the study with time expenditure**.

Some of the examinations are part of the normal outpatient appointments and therefore do not require any additional time.

| | V0 | V1 | V2 | V3 | V4 |
|---|---|---|---|---|---|
| | Screening visit and basic examination | Randomization at the beginning of the intervention period | Follow-up examination | End of the intervention period | End of the monitoring phase |
| | - 5 weeks until day 1 | Day 1 | 3±1 months | 6±1 months | 12±1 months |
| Checking the inclusion and exclusion criteria (5-10 minutes) | X | | | | |
| Educational talk (15 minutes) | X | | | | |
| Medical history including number of exacerbations in the last year, current medication, concomitant illness, etc. (20 minutes) | X | | X | X | X |
| Physical examination (10 minutes) | x | | X | X | x |
| ECG (10 minutes) | x | | | | |
| Pulmonary function test (20 minutes) | x | | X | X | x |
| Motor skills test (45-60 minutes) | x | | X | X | x |
| Questionnaire on sporting activity and training preferences (5 minutes) | X | | | | |
| Handing out the activity tracker and introduction to its use (15 minutes) | X | | | | |
| Quality of life questionnaire (QoL) (15 minutes) | X | | X | X | X |

**Are there any risks?**

Possible risks may occur during the motor function test, physical activity, lung function and training. However, we try to keep the risks as low as possible.

During the stress test, headaches, dizziness, nausea and increased coughing may occur. If these symptoms occur, the stress test is stopped immediately.

Problems can occur when carrying out the activity program that are not necessarily related to PCD. Risks during sport can include muscle injuries, ligament injuries in the ankle joint, fractures, dislocation of joints (e.g. ankle joint).

GEFÖRDERT VOM Bundesministerium für Bildung und Forschung

Risks associated with exercise that are known to occur with other chronic lung diseases such as cystic fibrosis include: Shortness of breath, coughing on exertion, dizziness, drop in blood sugar, pain in the joints, fractures in the event of a fall.

To reduce these risks, we will discuss them with you and your child before the start of the exercise program and make recommendations on how to avoid them.

Please do not allow your child to take part in any sporting activity if they have the following symptoms:

- Fever or other infections that are accompanied by a feeling of illness
- Acute pulmonary exacerbation
- Severe joint, back or headaches during or immediately after sport
- Chest pain or pain in the left arm
- Dizziness/fainting
- Shortness of breath (beyond the expected level) or hyperventilation
- Hypoglycemia/ hypoglycemia
- Persistent palpitations or palpitations after exercise
- Pacemaker/ICD depending on the sport

**Why do we need a control group?**

The "Move PCD" project aims to investigate the effects of regular physical activity on physical performance, lung function and other parameters. These effects can only be determined by comparing participants in the activity program with those who do not take part in the activity program. In this way, we can determine whether the positive effects are actually present. For this reason, we need a control group.

**What else do I have to do as a participant in the control group?**

We would like to ask your child to take part in the **study visits** and to wear the **"activity tracker**" during the entire study period. This small device measures daily movements, heart rate and sleep duration. Participation in the control group **does not** mean that your child is no longer allowed to do any sport. In fact, it is important that they **continue to** be **physically active on a regular basis** as before the study, as recommended by their doctor.

**Is there insurance?**

Special volunteer insurance was taken out for all participants. This covers all examinations at the *name study center* as well as physical activity during the study and exists for

Ecclesia Versicherungsdienst GmbH

Ecclesiastrasse 1 - 4, 32758 Detmold

Phone +49 5231 603-0, Fax +49 5231 603-197

info@ecclesia.de

Order no. NEV071767A

In addition, separate travel insurance was taken out for the journey to and from the examination location.

**SV SparkassenVersicherung Holding AG**
Löwentorstraße 65
70376 Stuttgart
Phone 0711 898-100
Fax 0711 898-109

Order no. 50 103 108/495

One accompanying person is also insured for the accompaniment via Ecclesia Versicherungsdienst GmbH (insurance no. ***0032369003 - 2023/1***).

You will receive copies of the insurance policy at the beginning of the study. If you suspect that an insured event has occurred, please contact your supervising study center, the leading study center in Bochum or the insurance company immediately.

**How will the collected data in this study be used?**

All data collected is subject to medical confidentiality and are treated as such. A data protection concept is available. This can be viewed on request. All data collected is stored on paper or electronically. They are evaluated without mentioning names and other personal data such as date of birth or address (pseudonymized). Name and date of birth are replaced by a letter/number code (so-called subject identification number). This makes it impossible or difficult to draw conclusions about the identity (§ 3 BDSG). The pseudonymization takes place after the personal data has been entered into a separate and secure central database ("contact database") by the AMIB. Only selected groups of people are given access to this contact database containing the contact

details of all participants. The lead study center in Bochum and the training institute "Prof. Dr. Baum" will have access to the contact details of all participants for the duration of the study in order to coordinate appointments, to carry out the intervention and after examination to release the intervention in the event of health problems. The local study centers only know the subject identification number of their study participants in order to be able to enter the data of the participants from their center into the study database.

In the study database, all data collected as part of the study will only be stored with the pseudonymization code. Access to the study database is also authorized separately. The study team at the *Name Study Center* may only restore the link between personal data and subject identification in precisely defined cases, e.g. if a participant falls ill.

For the scientific evaluation, presentation and publication of the data and study results, the participants' data will only be used in pseudonymized form.

In all surveys and studies, the medical-scientific research data is never linked to identity data, but only to the subject identification. The identity data is stored for 10 years after the end of the study. By deleting the identity data, the data is anonymized, i.e. it can never again be assigned to a person. The anonymized data will be deleted at the earliest 10 years after the last scientific publication.

**Information on data protection and use of the GARMIN Connect app and the GARMIN Junior app in connection with the activity trackers as part of the study**

The GARMIN Connect app must be installed in order to synchronize your child's activity tracker data. To record your child's activity data, you as a parent/ the person with custody must create an account. This requires an e-mail address and name (including nickname, for example). Please do not use 2-factor authentication, in which case no telephone number will be collected. It is not permitted to enter data of children under the age of 16 in the GARMIN Connect app. If your child is under 16 years old, you must also install the GARMIN Junior app. Here you log in with your GARMIN Connect name and assign a family name (can also be fictitious). You can then create a (fictitious) name for your child in the Junior app and link it to the "activity tracker". You also need to enter your child's date of birth, gender, wake-up and bedtime and, if possible, height and weight so that your child's activity and energy consumption can be measured.

The data protection guidelines of the GARMIN Connect app and the GARMIN Junior app apply. You will receive these and the GARMIN data protection guidelines from us. Please read them carefully with your child.

You are using the apps as part of a study, so please note the following points:

- GARMIN is an international company and processes data not only in Europe, but also in the USA and Australia.
- GARMIN also processes data in its own interest, e.g. to improve products or detect fraud. You generally consent to this by using the app. You will not be informed or asked for your consent each time GARMIN processes data.

- As part of this study, please withdraw your consent for your child to receive marketing information, insights and email notifications.
- Do not link any third-party apps, especially social networks such as Facebook, with the GARMIN Connect app and do not connect with other users of the GARMIN Connect app.
- Set your profile and that of your child to anonymous (Connect and Junior App).
- During the study period, your child should not take part in challenges (including "Toe-to-Toe") and should not use location services (weather, etc.). Nor should they use real-time tracking (Assistance Plus services) with an emergency contact.
- The GARMIN Connect app uses Google Analytics. This is prohibited in the context of studies. You must therefore withdraw your consent to the use of data. You can do this by using the browser add-on to deactivate Google Analytics.
- The data will be stored by GARMIN for as long as the account is active. You can only delete your child's data from GARMIN yourself. You can do this in the GARMIN Junior app account. At the end of the study, please delete the GARMIN Junior and Connect app accounts completely.

We buy the "activity tracker" for your child. The topic of payment processing and shipping in GARMIN's privacy policy therefore has no relevance for you and your child.

The data from the app is automatically transferred to a central database (Fitrockr Health Solutions) and stored there pseudonymized, i.e. only with the letter/number code of your child. Fitrockr Health Solutions is a company based in Germany. Data management takes place exclusively in Germany. The data in the central database are managed by the leading study center of the University Children's Hospital Bochum and the AMIB and, like all other data, can be deleted at your request - if possible (see below).

**Your rights with regard to data protection**

Consent to the processing of your child's data is voluntary. You can withdraw your consent at any time without giving reasons and without any disadvantages for your child. After that, no more data will be collected. If you withdraw your child's participation in this study, the data will be deleted as soon as this is possible. If retention periods prevent deletion, the data will no longer be used and will be deleted immediately after expiry. This does not affect the lawfulness of the processing carried out on the basis of the consent until revocation. You have the right to receive information about the data, also in the form of a copy free of charge. In addition, you can request the rectification, blocking, restriction of processing or erasure of the data and, if necessary, the transfer of the data. This means that you can have the data made available to you in a transferable form or have it sent to a location specified by you. This also applies if you wish the findings to be sent to the attending physician at a later date.

**The person responsible for data processing in the project is the project management**:

Prof. Dr. med. Folke Brinkmann
University Clinic for Pediatrics and Adolescent Medicine at St. Josef Hospital
Catholic Hospital Bochum
Department of Pediatric Pneumology

Here please find the logo of the insert own center

Alexandrinenstr. 5
44791 Bochum

**The person responsible for data processing at the Study Center is**:

Local study management

**The data protection officer of the** hospital/study center **is**:

xxxx

**Responsible data protection supervisory authority**:

xxxx

**Data protection supervisory authority of the federal state of xxx**:

Xxxxx

**Who do I contact if I have any questions?**

The study physicians and investigators will be happy to answer any further questions you may have. General questions, including questions concerning your rights as a participant, will also be answered by the following persons:

Study physician:in:

Local

Study office of the hospital:

local

GEFÖRDERT VOM
Bundesministerium für Bildung und Forschung

Here please find the logo
of the
insert own center

# Appendix

**Leading Study Center:**

University Clinic for Pediatrics and Adolescent Medicine at St. Josef Hospital

Catholic Hospital Bochum

Department of Pediatric Pneumology

Alexandrinenstr. 5

44791 Bochum

**Other participating study centers:**

University Medical Center Schleswig-Holstein, Lübeck Campus

Section for Pediatric Pneumology and Allergology

Ratzeburger Allee 160, House A

23538 Lübeck

Charité University Medicine Berlin

Clinic for Pediatrics with a focus on Pneumology, Immunology and Intensive Care Medicine

Campus Virchow Clinic

Augustenburger Platz 1

13353 Berlin

Hanover Medical School

Clinic for Pediatric Pneumology, Allergology and Neonatology

and

Clinic for Pneumology and Infectiology

Carl-Neuberg-Str.1

30625 Hanover

GEFÖRDERT VOM
Bundesministerium für Bildung und Forschung

Here please find the logo of the
insert own center


Clinic for Pediatrics and Adolescent Medicine at Münster University Hospital

Pediatric pneumology

Albert-Schweitzer-Campus 1, Building A1

48149 Münster

Marienhospital Wesel gGmbH

Pediatrics and adolescent medicine

Pastor-Janßen-Str. 8-38

46483 Wesel

Gemeinschaftsklinikum Mittelrhein

Clinic for Pediatrics and Adolescent Medicine Kemperhof

Koblenzer Strasse 115-155

56073 Koblenz


**Statistical management:**


Prof. Dr. rer. nat. Nina Timmesfeld

Ruhr University Bochum

Department of Medical Informatics, Biometry and Epidemiology (AMIB)

University Road 105

4789 Bochum


**Cooperation partner:**


Training Institute Prof. Dr. Baum

Wilhelm-Schlombs-Allee 1

50858 Cologne



GEFÖRDERT VOM Bundesministerium für Bildung und Forschung

Here please find the logo of the insert own center

Kartagener Syndrome and Primary Ciliary Dyskinesia e. V.

Königswarter Str. 5

65366 Geisenheim

**Sponsor:**

Ruhr University Bochum

University Road 150

44801 Bochum

**Funded by**: DLR Project Management Agency, Health Division

**Commissioned by**:  Federal Ministry of Education and Research

Here please find the logo of the insert own center

Study-ID____________________

**Move-PCD**
**A multicenter randomized controlled longitudinal study of the effect of a six-month individualized and supervised activity program on quality of life in children, Adolescents and adults with primary ciliary dyskinesia (PCD)**

## Information for adolescents (13-17 years)

Version 1.1 09.11.2023

Dear ________________________________,

you have a PCD and are 13 years or older. We invite you to take part in an investigation (study). We are investigating whether exercise helps you to feel better with your illness.

**Your participation in this study is voluntary. Take your time to think about it together with your parents/guardians. Don't' hesitate to ask us questions if you do not understand anything. Of course, you do not have to take part. But we would be very pleased if you would participate.**

**At any time you can tell us, that you no longer want to take part in the study. You do not need to have a reason for this. There won't be any disadvantages for your treatment.**

PCD is a rare disease. That is why we are conducting the study at seven locations in Germany. These are Bochum, Lübeck, Berlin, Hanover, Münster, Wesel and Koblenz. The "Kartagener Syndrom und Primäre Ciliäre Dyskinesie" e.V. is helping us with the study. You can also register at the congress for patients in Hamburg 2024.

The study is funded by the German government. More specifically, the Ministry of Education and Research (BMBF).

All participants are divided into two groups. This is done by drawing lots. In one group, everyone receives their own activity program. This group is called the intervention group. The other group is called the control group. Everyone in the control group is told to exercise. Just like your doctor says to you. Then we compare the two groups.

The sports program is created by scientists and trainers from the Prof. Dr. Baum Training Institute in Cologne. Each participant is given their own activity program. We will consider what suits you and your interests. The scientists work together with the doctors of the leading study center (University Clinic for Pediatrics and Adolescent Medicine Bochum) and the doctors of the *study center*(s).

GEFÖRDERT VOM Bundesministerium für Bildung und Forschung

**Aim of the study**

Exercise and sport help to improve physical, mental and social health for everyone. In the case of chronic lung diseases, they can help to improve lung function and slow down the progression of the disease. We know this from other diseases such as cystic fibrosis (CF) and bronchial asthma.

Sport and exercise are currently an integral part of the treatment of PCD. That's why your doctor recommends that you exercise. However, physical exertion in everyday life is also important for your fitness. Cycling, climbing stairs, playing soccer on the football pitch or riding a waveboard can help.

Many teenagers with PCD are less active than healthy teenagers of the same age. We look at whether regular contact and help from a trainer can help you. The aim is to clear obstacles and problems out of the way. So that these don't stop you from taking part in sport.

To date, there has been no scientific research into the effects of exercise on PCD. This means that no one has yet tested whether a special exercise program for PCD is good for lung function, improves well-being and slows down the progression of the disease. We would like to change this.

**Who is not allowed to participate in this project?**

Unfortunately, there are illnesses that you may have in addition to PCD. With some illnesses you will unfortunately not be able to take part in our project. Your parents/guardians have received a list. Discuss with them whether anything on the list applies to you. If you are unsure, talk to your doctor or us.

**Benefits from the study**

You can improve your physical fitness and lung function with the study. You may also become fitter for things like school or meeting friends. It is also possible that your quality of life and well-being will improve. A good quality of life roughly means that you are doing well in many areas of life. For example, you have great friends and feel happy and healthy. If the activity program proves to help people with PCD, we hope that the health and care insurance companies will provide more money. This would allow more people with PCD to do sport as therapy.

**Procedure of the study**

If you would like to take part in the study, please discuss this with your parents/guardians. We need a signature from them and from you stating that you want to take part and are allowed to do so.

Then you will be examined by us. We will check whether you have any health problems. These could make it too dangerous for you to take part in the study. This is called a screening visit. This can take place either at your supervising study center, at the leading study center (University Clinic for Pediatrics and Adolescent Medicine Bochum) or at the Congress for Patients 2024 in Hamburg.

We, the study team at the Name Study Center, will ask you and your parents/guardians about other illnesses and current medical conditions. We also want to know what medication you are taking. Please continue your

normal therapy during the study as before. If anything changes, please let us know. You will also be examined by a doctor. We will measure your **lung function** (if possible with measurement of the Lung Clearance Index (**LCI**)) and examine your heart **(ECG)**. We will then make a final decision as to whether you can take part or not. It may also be that further examinations have to be carried out first. You may then be able to take part in the study at a later date.

You and your parents/guardians will receive a **questionnaire on quality of life**. It's easy to do online. You will receive a personal link. You are also welcome to use one of our tablets for this. The questionnaire was specially developed for young people with PCD (QoL-PCD). The developers from Canada would like to analyze the results of these questionnaires from all participants anonymously. They would like to further improve and adapt the questionnaire. For this purpose, the results will be forwarded without your personal data (i.e. without your name, date of birth or contact details). They therefore do not know that these are your answers.

Employees of the training institute "Prof. Dr. Baum" from Cologne will do a **motor skills test** with you. This is how we test your current fitness. This test consists of 6 different tests. Each test will be explained and shown to you again before you start. You will also receive two **questionnaires.** We want to find out how much you exercise and what you would like to do. We use the test and the questionnaires to create your personal activity program. The motor skills test may take place on a different date. We, the study team at the *Name Study Center*, will inform you and your family in good time if this is the case.

1) Standing long jump: You stand with both toes on a starting line. Then jump off with both feet at the same time. We measure the distance (in cm) from the starting line to the toe of the back leg. The best of 3 attempts counts.

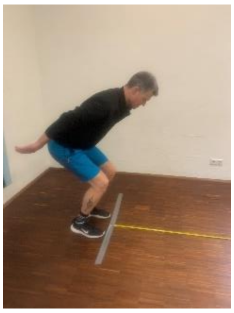

2) Push-ups: Two small cushions (Theraband stability trainer (ThS)) are placed on top of each other (together 10 cm high). Start with your arms outstretched. Your body is stretched out and the tips of your feet are shoulder-width apart on the floor. With your body straight, bend your arms until your chest touches the ThS. Then straighten them again. You can decide how fast you do this. The number of valid attempts within 30 seconds is measured.

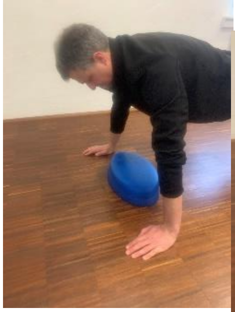

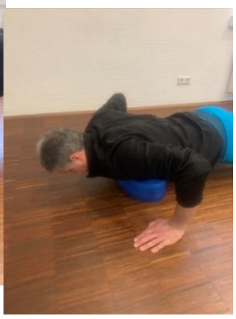

GEFÖRDERT VOM
Bundesministerium für Bildung und Forschung
DLR
TRAININGSINSTITUT Prof. Dr. Baum
Leben mit PCD Kartagener Syndrom und Primäre Ciliäre Dyskinesie e.V.

3) <u>One-legged stand:</u> Stand two meters away from a marker and focus on it with your eyes. Then lift one leg up. The time in the one-leg stand is measured. Stop after 60 seconds at the latest. Both legs are first tested with eyes open. The test is then repeated with the eyes closed by a blindfold.

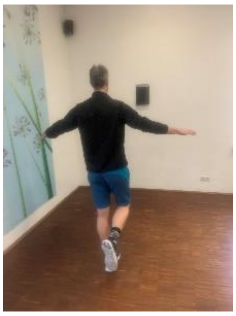

4) <u>Sit-up:</u> You lie on your back with your knees bent and your head resting on two cushions (Theraband stability trainers (ThS)). Your feet are held in place by the person conducting the test. During the test, you raise your upper body until both hands touch your ankles. Then the upper body is lowered again until your head touches the ThS. The number of times within 30 seconds is counted.

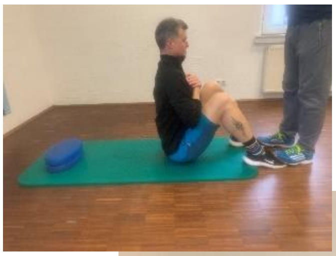

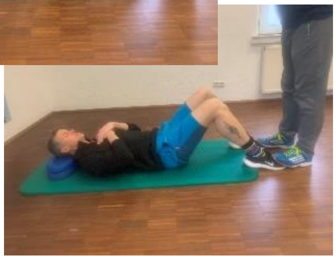

5) <u>Torso squat:</u> Stand on a box with your knees straight. Then bend your upper body forward and down as far as possible. The distance (in cm) is measured at the height of the tip of the middle finger, without rocking.

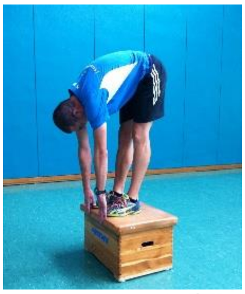

6) <u>10-minute run:</u> You run around a marked field for 10 minutes. You can set your own pace. The aim is to cover as much distance as possible. You can walk and run. You can also alternate. The distance is measured in 10 minutes.

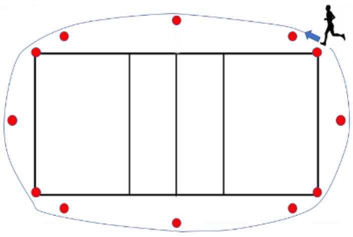

At the first appointment, you will receive a GARMIN **activity tracker** (Vivofit 4). The activity tracker is worn like a wristwatch. It can measure your heart rate, how long you sleep and count your steps. It would be great if you put the activity tracker on straight away. Ideally, you should wear it day and night during the study - i.e. for 12 months. We would also like to ask you and your family to install the app or connect the activity tracker to a computer. Your data from the "activity tracker" and the login data of your parents/guardians will then be saved

in the app. After the study or if you no longer wish to take part, you can delete the app. All your data will then be deleted from GARMIN. The data from the app is automatically sent to a central database. We can see it there. This helps us to better design your individual activity program. Of course, you will also receive an instruction manual. The staff at the training institute "Prof. Dr. Baum" will explain to you how to wear the activity tracker and how it works.

Four weeks later, we will randomly assign the groups. We will call you and your family to inform you.

If you are part of the **intervention group,** the Training Institute Prof. Dr. Baum will contact you afterwards. You will receive your **personal training plan**. You will also be given an appointment for a **meeting with** your personal trainer. The meeting lasts 20 minutes and takes place **once a week**. Sometimes it may be necessary for your parents/guardians to attend the meeting. These appointments are important. You can talk about problems with your activity program here. Your activity program can be adjusted by your trainer at any time according to your wishes. The coach will also ask you (and your parents/guardians if applicable) about your well-being during the sport. You may be asked to take a break if you have health problems. The coach will pass this information on to the University Clinic for Pediatric and Adolescent Medicine in Bochum. Here, doctors will then decide whether you can continue the activity program without any problems. They will call you and your family to then inform you within a few days.

Once a week, you (or your parents/guardians) will receive an email with a link to the online platform "taskcards". Here you will find **links to three videos per week**: the online courses "Enjoy the day", "Strengthen your body" and "Move the Rhythm". Each video lasts 45 minutes. The courses are built on each other. You can decide for yourself when and whether you do the courses. The videos are created especially for this study. Please do not pass on the links. Especially not to participants from the control group. The control group will also receive all the links themselves at the end of the study. It would be great if you could comment briefly on the courses you take part in under "taskcards" (e.g. "good", "too strenuous", etc.). This will help us to improve the courses and to get an overview of which courses are suitable for young people with PCD.

During the study, it is important for us to know how you are doing and how exercise affects your quality of life and well-being. Of course, we also want to know whether you have any problems and, if so, what they are. We would therefore like to examine you at regular intervals. You are welcome to receive the results from us afterwards.

3, 6 and 12 months after you have been assigned to a group, we will invite you and your parents/guardians to check-up appointments. These are known as study **visits**. These can be combined with your outpatient appointments with us at the Name Study Center.

We will then **question and examine** you again. We will check your **lung function** and repeat the **motor function test**. We will also ask you and your parents/guardians to complete **the quality of life questionnaire** each time. Please bring the "activity tracker" with you to every appointment. Please return it to us at the last visit.

It is possible that the motor skills test will take place on an extra appointment. We will then inform you and your family ahead.

If you have decided to participate in the Patient Congress, we will find a solution with your parents/guardians as to how and where the study visits can take place.

**How much time does it take?**

You can decide for yourself how much you want to exercise. Your personal activity program is tailored to your wishes. The online courses last 45 minutes each. You can take part in three courses per week. During the program, you talk to your trainer once a week for 20 minutes by phone or video about problems and any questions you may have.

The four study visits (screening visit, as well as those after 3, 6 and 12 months) do not take significantly longer than a regular outpatient appointment. You can also combine them. Allow 60 minutes per visit. You need to allow half a day for each of the motor function tests. The test itself only takes about 1.5 hours. However, several participants are tested on one day. This can result in waiting times.

**Table 1: Examinations and duration of the study.**

Some of the examinations are part of your normal outpatient appointments.

| | V0 | V1 | V2 | V3 | V4 |
|---|---|---|---|---|---|
| | Examination before the start of the study | Division into the groups | Follow-up examination | End of the supervised training period | Last examination at the end of the study |
| | - 5 weeks until day 1 | Day 1 | 3±1 months | 6±1 months | 12±1 months |
| Check whether you can take part (5-10 minutes) | X | | | | |
| Educational talk (15 minutes) | X | | | | |
| Medical history (questions about how you are currently feeling, what medication you are taking and more) (20 minutes) | X | | X | X | X |
| Physical examination (10 minutes) | x | | X | X | x |
| ECG (10 minutes) | x | | | | |
| Pulmonary function test (20 minutes) | x | | X | X | x |
| Motor skills test (45-60 minutes) | x | | X | X | x |

| | | | | | |
|---|---|---|---|---|---|
| Questionnaire about what sports you already do and what you would like to do during the study (5 minutes) | X | | | | |
| Handing out and explanation of the activity tracker (15 minutes) | X | | | | |
| Quality of life questionnaire (QoL) (15 minutes) | X | | X | X | X |

**Are there any risks?**

Possible risks may occur during the motor function test, the activity program and the lung function. However, we try to keep the risks as low as possible. For example, headaches, dizziness and nausea may occur. Physical exertion can also cause increased coughing. In the event of a strong cough, the exercise is stopped immediately.

Risks to health can occur during training that are not necessarily related to PCD. Risks during sport can include muscle injuries, ligament injuries in the ankle joint, ankle and lower leg fractures and dislocation of the shoulder joint.

Risks associated with chronic lung diseases during sport can include: Shortness of breath, coughing on exertion, dizziness, drop in blood sugar, pain in the joints, fractures in the event of a fall.

We will talk to you about this before training and give you recommendations on how you can reduce these risks. Your parents/guardians will also receive a list of when you are not allowed to do sports. Read it through together.

**Why do we need a control group?**

The "Move-PCD" project aims to investigate the effects of regular physical activity on physical performance. At the same time, we are also investigating the effects on well-being, lung function and other parameters. These effects can only be determined by comparing participants in the sports program with those who do not take part in the activity program. In this way, we can determine whether the positive effects are actually present. That is why we need a control group.

**What else do I have to do as a participant in the control group?**

We would like to ask you to take part in the **study visits**. In addition, you should wear the **"activity tracker" during the** entire study period. This small device measures your movements in everyday life, your heartbeat and the duration of your sleep. As a participant in the control group, you should still **continue to exercise**. Be just as active as you were before the study and as recommended by your doctor.

**Is there insurance?**

Special volunteer insurance has been taken out for all participants. You are covered for all examinations at the *Name Study Center* and for physical activity during the study.

Ecclesia Versicherungsdienst GmbH

Ecclesiastrasse 1 - 4, 32758 Detmold

Phone +49 5231 603-0, Fax +49 5231 603-197

info@ecclesia.de

Order no. NEV071767A

You and your companion are additionally covered by a separate travel insurance for the journey to and from the examination location. The insurance was taken out with

**SV SparkassenVersicherung Holding AG**
Löwentorstraße 65
70376 Stuttgart
Phone 0711 898-100
Fax 0711 898-109

No. 50 103 108/495 was concluded.

**How will the collected data in this study be used?**

All people involved must adhere to medical confidentiality and data secrecy. All your data and measurement results will be pseudonymized. This means that your name will be replaced by an individual sequence of numbers and letters. No one who is not part of the study team will know that you are taking part in the study and what we have measured in you. The number-letter code is assigned via a central database ("contact database"). Only your contact details and the code are stored here. Only the study team of the study center has access to this data. This enables the study team to find out which number belongs to you. In addition, the training institute “Prof. Dr. Baum” and the leading study center in Bochum have access to your contact details during the study. This is important so that appointments can be made with you and your family for the motor skills tests and the weekly interviews. The study team in Bochum must also be able to contact you and your family if you have any health problems during the study. This is the only way we can ensure that the activity program is not dangerous for you at any time.

An external person cannot know from the number that it is you. The pseudonymized data is stored securely for 10 years. After that, all information that could be used to identify you (e.g. your name and telephone number) will be deleted. The study data is then anonymized, i.e. it can never again be assigned to a person. The anonymized data will be deleted at the earliest 10 years after the last scientific publication.

**What do you need to know about using the GARMIN Connect app and the GARMIN Junior app when using the "activity tracker"?**

In order for us to analyze your data from the "activity tracker", your parents/guardians must install the GARMIN Connect app and create an account with their name and email address. Your parents then enter the following data about you in the app Date of birth, height, weight, gender, wake-up and bedtime. This data may only be entered if you are over 16 years old. If you are under 16, you also need the GARMIN Junior app. Your parents can then create a profile for you and enter the data there. We need the data to understand how active you are and how many calories you consume per day.

The data you enter and the data measured by the "activity tracker" are subject to the data protection guidelines of the GARMIN Connect app and the GARMIN Junior app. You and your parents/guardians will receive a copy of this. Please read these together at your leisure.

As you are using the apps as part of a study, you must observe a number of rules. All rules only apply if your parents/guardians agree and install the app(s). Please do not install any of the apps on your own and do not enter any data without your parents/guardians!

- GARMIN is a company that operates worldwide. Data is therefore processed in Europe as well as in the USA and Australia.
- GARMIN also processes data in its own interest, e.g. to improve products or detect fraud. If you use the app, you consent to this. You will not be informed or asked for your consent each time GARMIN processes data.
- We do not want you to receive marketing information, insights or email notifications. You must withdraw your consent for this.
- Please do not link any third-party apps, especially social networks such as Facebook, with the GARMIN Connect app and do not connect with other users of the GARMIN Connect app.
- Set up your profile anonymously together with your parents/guardians (Connect and Junior App).
- Do not take part in challenges during the study (not even "Toe-to-Toe") and do not use any services that want to know your location. These are, for example, the weather or real-time tracking (Assistance Plus services) with an emergency contact.
- The GARMIN Connect app uses Google Analytics. This is prohibited in the context of studies. You must therefore withdraw your consent to the use of data. You can do this by using the browser add-on to deactivate Google Analytics.
- The data is stored by GARMIN for as long as the account is active. Only your parents/guardians can delete your data from your GARMIN account. At the end of the study, your parents/guardians must delete the GARMIN Junior and Connect app accounts completely.

GEFÖRDERT VOM
Bundesministerium für Bildung und Forschung

We buy the "activity tracker" for you. You are therefore not affected by the issue of payment processing and shipping in GARMIN's privacy policy.

The data from the app is automatically sent to a central database (Fitrockr Health Solutions). It is only stored there with your number-letter code. Fitrockr Health Solutions is a company based in Germany. The data in the central database is used by you in the same way as all other data (see above).

**Your rights regarding data protection**

You have the right to information about your personal data. The right to have the data deleted if you wish. If data has already been published, it can no longer be completely deleted. You have the right to request that we only process the data to a limited extent. The right to object to data processing. The right to be informed about everything that happens to your data. This complies with the EU General Data Protection Regulation. If you wish to exercise this right, please contact the Student Office.

**Possibility for further questions**

If you have any questions, you can contact the study team at any time:

Study physician:in:

Local

Study office of the hospital:

local

Here please find the logo of the insert own center

## Appendix

**Leading study center:**

University Clinic for Pediatrics and Adolescent Medicine at St. Josef Hospital

Catholic Hospital Bochum

Department of Pediatric Pneumology

Alexandrinenstr. 5

44791 Bochum

**Other participating study centers:**

University Medical Center Schleswig-Holstein, Lübeck Campus

Section for Pediatric Pneumology and Allergology

Ratzeburger Allee 160, House A

23538 Lübeck

Charité University Medicine Berlin

Clinic for Pediatrics with a focus on Pneumology, Immunology and Intensive Care Medicine

Campus Virchow Clinic

Augustenburger Platz 1

13353 Berlin

Hanover Medical School

Clinic for Pediatric Pneumology, Allergology and Neonatology

and

Clinic for Pneumology and Infectiology

Carl-Neuberg-Str.1

30625 Hanover

Here please find the logo of the insert own center

Clinic for Pediatrics and Adolescent Medicine at Münster University Hospital

Pediatric pneumology

Albert-Schweitzer-Campus 1, Building A1

48149 Münster

Marienhospital Wesel gGmbH

Pediatrics and adolescent medicine

Pastor-Janßen-Str. 8-38

46483 Wesel

Gemeinschaftsklinikum Mittelrhein

Clinic for Pediatrics and Adolescent Medicine Kemperhof

Koblenzer Strasse 115-155

56073 Koblenz

**Statistical management:**

Prof. Dr. rer. nat. Nina Timmesfeld

Ruhr University Bochum

Department of Medical Informatics, Biometry and Epidemiology (AMIB)

University Road 105

4789 Bochum

**Cooperation partner:**

Training Institute Prof. Dr. Baum

Wilhelm-Schlombs-Allee 1

50858 Cologne

Here please find the logo of the insert own center

Kartagener Syndrome and Primary Ciliary Dyskinesia e. V.

Königswarter Str. 5

65366 Geisenheim

**Sponsor:**

Ruhr University Bochum

University Road 150

44801 Bochum

**Funded by**: DLR Project Management Agency, Health Division

**Commissioned by**: Federal Ministry of Education and Research

Here please find the logo of the insert own center

Study- ID___________

**Move-PCD**
**A multicenter randomized controlled longitudinal study of the effect of a six-month individualized and supervised activity program on quality of life in children, Adolescents and adults with primary ciliary dyskinesia (PCD)**

**Information for children (7-12 years)**
Version 1.1 09.11.2023

Dear ________________________________,

You have a PCD and you are 7 years or older. We invite you to take part in an examination (study). A study involves a lot of tests. This helps us to understand things better. We learn new things about PCD every day and you can help us to understand this illness better.

**What do we want to investigate?**

Sport and exercise can help with lung diseases. You feel healthier and your lungs work better. We would like to see whether this is also the case for PCD patients. This is only possible if patients with PCD take part in the study.

We randomly divide everyone into two groups. The first group is given its own sports program. This is the intervention group. The second group is the control group.

**What happens in the intervention group?**

You get your own **sports plan**. It's like a timetable for your sport. A personal trainer will plan this especially for you. You can decide which sport you will do. You will talk **to your trainer on the phone once a week**. You can ask this trainer anything. You can also do **sports courses online** every week. You can do this at home in front of the TV or computer. You get an "**activity tracker**". This is like a watch. You can wear it on your wrist. It measures your movement, your heartbeat and your sleep. Please wear it all day and night.

**What happens in the control group?**

The control group is **not** given **its own sports plan**. You should still exercise. Just like your PCD doctor always tells you. You will also be given an **activity tracker**. This allows us to measure how much exercise you have done. This is the only way we can compare the two groups with each other.

GEFÖRDERT VOM
Bundesministerium für Bildung und Forschung

Here please find the logo of the insert own center

### Who can take part?

All patients with PCD between 7 and 55 years of age.

### Where can I take part?

You can take part in 7 PCD outpatient clinics in Germany. Your parents have a list. You can also call or write to us. You can do the sport at home or in your sports club as usual.

### How often do I need to be examined?

At the beginning of the study, we will examine you every 3 months. No matter which group you are in. These **examinations** take place in the PCD outpatient clinic. You already know most of it. We will examine you. We measure your height and weight. We ask you and your parents how you are doing. We ask what medication you are taking. You blow a **lung function test**. We examine your **heart**. Then we do a **sports test**. We measure how fit you are. You will do six exercises

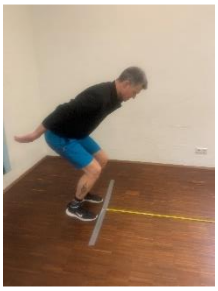

**1**

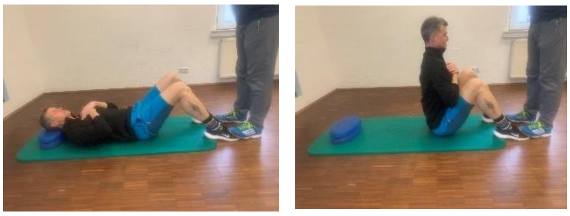

**2**

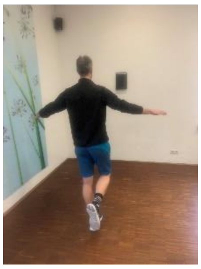

**3**

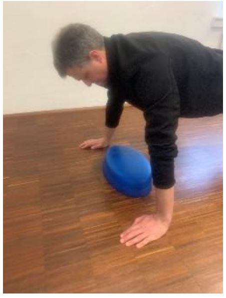

**4**

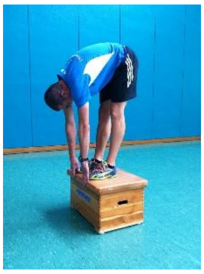

**5**

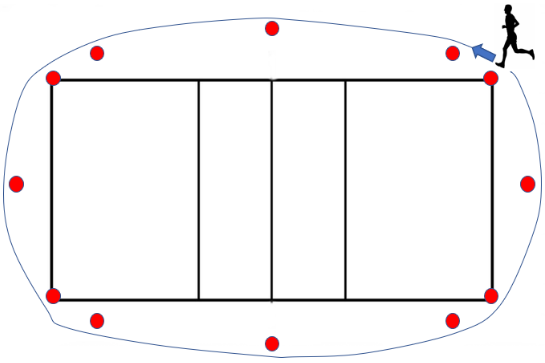

**6**

GEFÖRDERT VOM
Bundesministerium für Bildung und Forschung
DLR
TRAININGSINSTITUT Prof. Dr. Baum
leben mit PCD Kartogener Syndrom und Primäre Ciliäre Dyskinesie e.V.

Here please find the logo of the insert own center

**Table 1: Examinations and duration of the study.**

Some of the examinations are part of your normal outpatient appointments.

| | **V0** | **V1** | **V2** | **V3** | **V4** |
|---|---|---|---|---|---|
| | **Examination before the start of the study** | **Division into the groups** | **Follow-up examination** | **End of the supervised training period** | **Last examination at the end of the study** |
| | **- 5 weeks until day 1** | **Day 1** | **3±1 months** | **6±1 months** | **12±1 months** |
| Check whether you can take part (5-10 minutes) | X | | | | |
| Educational talk (15 minutes) | X | | | | |
| Medical history (questions about how you are currently feeling, what medication you are taking and more) (20 minutes) | X | | X | X | X |
| Physical examination (10 minutes) | x | | X | X | x |
| ECG (10 minutes) | x | | | | |
| Pulmonary function test (20 minutes) | x | | X | X | x |
| Motor skills test (45-60 minutes) | x | | X | X | x |
| Questionnaire about what sports you already do and what you would like to do during the study (5 minutes) | X | | | | |
| Handing out and explanation of the activity tracker (15 minutes) | X | | | | |
| Quality of life questionnaire (QoL) (15 minutes) | X | | X | X | X |

**How long does the study take?**

You decide for yourself how much sport you do. The whole study lasts one year. In the first six months you do the sports program. In the second six months you do sport on your own again. All participants wear the activity tracker for one year.

**Is the examination dangerous?**

The examinations at your doctors are the same as always. So they are not dangerous. You can injure yourself during sport. Please be careful! You may also have a cough or find it difficult to breathe. In this case, tell your parents or your coach immediately.

Here please find the logo of the insert own center

### What happens if I don't want to take part?

The study is voluntary. Take your time to talk to your parents about the pros and cons. You can ask us anything about this study. You do not have to take part. You can always say that you no longer want to take part. You don't have to say why. You will then be treated as usual in your PCD outpatient clinic.

### Who knows that I'm taking part?

No one will find out from us that you are taking part in the study. We will keep your name, date of birth and address secret.

When you use the activity tracker, data is also generated. This data is collected in an app on your parents' cell phone or computer. The app has its own rules. Your parents will be informed about this. You can ask them or us if you want to know more.

All your results from the tests are given a secret code. That way, only we know that these are your values. At the end, we want to publish the results of all participants together in a magazine.

### Why should I take part?

We cannot carry out this examination without PCD patients. Only you can help us. With your help, we can find out whether sport is good for you. We will learn more about PCD. We want to improve your treatment. Maybe we can get your sports courses covered by your health insurance.

### Who can I ask?

If you have any questions, you can always ask our study team:

Study physician:in:

Local

Study office of the hospital:

local

GEFÖRDERT VOM
Bundesministerium für Bildung und Forschung

Here please find the logo of the insert own center

## Proband - Information on the restriction of physical activity

**Dear study participant,**

**if you have the following complaints, we ask you not to do any sport or exert yourself physically:**

- Fever or other infections accompanied by a feeling of illness
- Acute pulmonary exacerbation
- Severe joint, back or headache during or immediately after exercise
- Chest pain or pain in the left arm
- Dizziness/fainting
- Shortness of breath (beyond the expected level) or hyperventilation
- Hypoglycemia/ low blood sugar levels
- Persistent palpitations or palpitations after exercise
- Pacemaker/ICD (implanted defibrillator) depending on the type of sport

**Please discuss this with your attending physician, if and when sport/physical activity will be possible again or contact us directly!**

**Please also contact us within 48 hours if you have to go to the hospital unplanned!**

Contact address Study Center:

Local Study Center

GEFÖRDERT VOM
Bundesministerium für Bildung und Forschung
