# Supplementary material for: Move-PCD—a multi-center longitudinal randomized controlled superiority trial on the effect of a 6-month individualized supported physical activity (PA) program on quality of life (QoL) in children, adolescents, and adults with primary ciliary dyskinesia
Source: Trials. 2024 Aug 15;25:539. doi: 10.1186/s13063-024-08379-0 (PMC11328395; doi:10.1186/s13063-024-08379-0)
Supplement: Supplementary file 5 — Supplementary Material 5 [file 13063_2024_8379_MOESM5_ESM.pdf]

Fitrockr Health Solutions
# – Data Protection Information for Projects

The following data protection information provides information about the use of the Fitrockr Health Solutions in research, data analysis, healthcare or clinical trial projects.

Service Provider

- Fitrockr Health Solutions software, services and products are provided by the registered company "Digital Rebels GmbH" (Commercial Register Germany: HRB 192795 B / Tax-ID DE316295291).
- The company headquarter is located at Friedrichstr. 114 A, 10117 Berlin, Germany.
- The company was founded in 2018.

Software

- The Fitrockr software is operated by the service provider as a specific project instance, which will be completely deleted at the end of the project, including all data.
- The Fitrockr software consists of the following tech stack:
  - Web App: Angular
  - Mobile Apps: Native Android App and Native iOS App
  - Backend: Dockerized Spring Application (Java-based), Rest API, NoSql-Database (MongoDB)
- Fitrockr Hub synchronization uses websockets and requires port 443.
- Fitrockr Live streaming requires port 443 and ack on ports 32768-65535.

Hosting

- Fitrockr cloud servers are self-managed (no Amazon, no Google) by the service provider and operated in Germany under EU data protection law.

- The servers are hosted in a professional ISO-27001 certified hosting center that complies with the highest security and data protection standards. The hosting center is provided by Hetzner Online GmbH, Industriestr. 25, 91710 Gunzenhausen, Germany.
- On request, Fitrockr can also be installed and hosted on any other on-premise or local cloud server.

Backups

- A data backup is automatically performed every 12 hours.

Access Restriction

- The Fitrockr platform distinguishes different roles such as user, administrator, coach, reporter, etc. with different access rights.
- Access is granted via login and password. The password must have a minimum length of 8 characters. User accounts are locked after 5 failed login attempts.
- 2-factor-authentication is available and can be enabled on request.

Data Flows

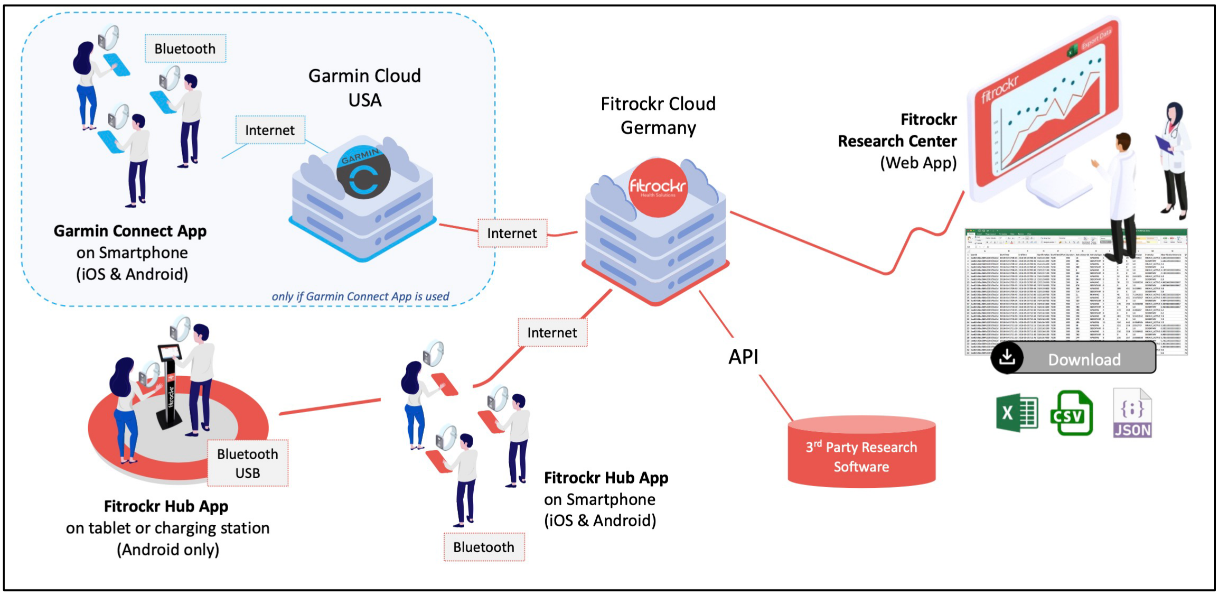

- Participants synchronize the Garmin device either (exclusive or) with the “Garmin Connect” app or with the “Fitrockr Hub” app.

- Synchronization between device and app is performed via Bluetooth or USB cable. Synchronized data is immediately forwarded by the app to the cloud via Internet.
    - If "Garmin Connect" app is used, data is sent to Garmin Cloud USA first and then automatically forwarded to the Fitrockr Cloud Germany.
    - If "Fitrockr Hub" app is used, data is sent directly to the Fitrockr Cloud. This option allows to completely bypass the Garmin cloud and allows exclusive data hosting and processing in a specific country of choice (by default: Germany).
- Data transfers between systems are encrypted and secured by API keys.
- Researchers can access synchronized data via the Fitrockr Research Center web application. Depending how user profiles have been set up by the researchers, data can be accessed personalized, pseudonomized or anonymized.
- Projects can also connect to the Fitrockr REST-API to automatically transfer data from the Fitrockr Cloud to their own database.

Data Protection Standards

- Fitrockr Health Solutions has been HIPAA certified.
- Fitrockr Health Solutions Germany Cloud has been ISO27001 certified.
- Fitrockr Health Solutions commits to follow Good Clinical Practice and GDPR.

Data Privacy

- Digital Rebels GmbH (Fitrockr) is a sole software service provider that performs data processing exclusively for the purpose of the project. Any further processing or use of the data is excluded. Upon request, a data protection agreement can be provided for the project.
- Digital Rebels GmbH (Fitrockr) has no interest or ownership in the data collected by the project. All data is exclusively owned by the project.
- User profiles can be set up and maintained anonymized, pseudonomized or personalized based on project need.
- Personal data collected is minimised and limited to what is lawful and strictly necessary.

- Personal and sensible information is only displayed and accessible to authorized administrators via the Fitrockr Research Center.
- Participants are informed about data protection when signing up for the data collection project via the Fitrockr Hub app.
- Participants have the right to request information, update or deletion of their data at any time.

- End of document -

PRIVACY POLICY

Last Updated: July 6, 2023

Your privacy is important to Garmin. We developed this Privacy Policy to provide you with information on how we process your personal data when you create a Garmin account, interact with our customer support specialists, make a purchase in one of our stores, visit Garmin.com or other Garmin websites, or use Garmin apps that include a link to this Privacy Policy.

Other Garmin websites, apps, and products that include a link to a different Garmin privacy policy are governed by that privacy policy. For example, we have a separate privacy policy that applies to Garmin Connect and its compatible wearables and other devices. Click here to review our other privacy policies.

"Personal data" is information relating to an identified or identifiable natural person. Additional information specific to your jurisdiction may be provided in a separate document. Please see our supplemental policy, "Your Data Protection Rights," for additional information that may apply to you. The legal grounds for processing personal data specified in this Privacy Policy are based on the EU General Data Protection Regulation ("GDPR") and laws in other jurisdictions that have similar grounds. If you are a California resident, please see our CCPA Privacy Notice.

To jump to a specific section of this Privacy Policy, please click on a link below:

- Categories of Personal Data Processed by Garmin
- Categories of Recipients of Personal Data
- International Transfers of Personal Data

- Cookies and Similar Technologies
- Monitoring and Analytics
- Children
- Privacy Policy Updates
- Retention of Personal Data
- Automated Decision-Making, Including Profiling
- Data Controller and Data Protection Officer
- Manage Your Data
- Recent Policy Versions

Categories of Personal Data Processed by Garmin

Personal data that is processed when you create a Garmin account:

When you create a Garmin account, we ask you to provide your email address, name, and password. You can choose to provide only your first name or a nickname instead of your full name if you wish. We may also ask you to provide your mobile phone number.

PURPOSES AND LEGAL GROUNDS:

(a) We process your email address and password because you use your email address and password to sign in to your account. The legal ground for processing your email address and password for this purpose is our legitimate interest in protecting the security of your account. If you enable two-factor authentication, we process your mobile phone number or email address to send the security code via SMS or email. The legal ground for processing this information for this purpose is our legitimate interest in protecting the security of your account. If you reside in mainland China, we process your mobile phone number for purposes of real name authentication.

(b) We also process your email address for the purpose of sending you important information about your Garmin products, services, apps, or account, such as important

safety information or material changes to this Privacy Policy. The name you provide is associated with your account profile and is displayed when you submit comments or other material on our websites or apps or engage with other users. The legal ground for processing your email address and name for these purposes is our legitimate interest in providing you important safety or other information about your Garmin products, services, apps, or account or material changes to this Privacy Policy and in providing you an opportunity to engage with other users.

(c) If you provide your opt-in consent to receiving marketing information from us, we will also process your email address for the purpose of sending you marketing information about our products, services, and apps, as well as newsletters. The legal ground for processing your email address for this purpose is your consent. You may withdraw your consent at any time by changing your preferences in your account or through the unsubscribe link at the bottom of our marketing emails. The marketing emails you receive from us are based on the preferences you provide in your account, the locale indicated by your Internet Protocol (IP) address, the types of Garmin devices you have added to your account, and any subscriptions included in your account. The legal ground for processing this data for this purpose is our legitimate interest in reducing the number of marketing emails sent to each particular customer by selecting which customers receive a particular marketing email rather than sending every marketing email to every customer who has consented to receiving marketing emails. If you reside in mainland China, we may use your mobile phone number to send you marketing communications via SMS.

(d) We also process your email address to associate it with your account when you interact with our customer support representatives. The legal ground for this processing is our legitimate interest in providing quality customer support.

(e) We also process your email address to notify customers when they have violated our terms. The legal ground for this processing is our legitimate interest in ensuring a quality experience for all customers and ensuring adherence to our terms.

Personal data that is processed when you sync your Garmin device:

When you sync your device through a compatible Garmin app, we log data about the transmission, such as the IP address used when syncing, the sync time and date, crash/diagnostic logs, geographic location of the device, information about your device, information about the network used to sync (e.g., Wi-Fi or cellular), and device battery level.

PURPOSES AND LEGAL GROUNDS:

We process this information to help identify and resolve errors or syncing issues. The legal ground for processing this information for this purpose is our legitimate interest in resolving errors or syncing issues and providing quality product support. We also process this information to analyze usage and trends and develop or improve features and services. The legal ground for this processing is our legitimate interest in providing relevant and quality features and services.

Personal data that is processed when you communicate with Garmin:

When you interact with our customer support representatives by email or telephone, online, or in person, we collect personal data, such as your name, mailing address, phone number,

email address, and contact preferences, and information about the products you own, such as their serial numbers and dates of purchase, and subscriptions you've purchased. We also may create event logs that are useful in diagnosing product or app performance-related issues and capture information relating to the support or service issue. To improve customer service, subject to applicable laws, we may also record and review conversations with customer support representatives and analyze any feedback provided to us through voluntary customer surveys. With your consent, our customer support representatives may sign in to your account, if appropriate, to help troubleshoot and resolve your issue.

PURPOSES AND LEGAL GROUNDS:

We process this information to provide you with customer and product support, to monitor the quality and types of customer and product support we provide to our customers, and to facilitate repairs, returns, or exchanges. The legal ground for processing this information for these purposes is our legitimate interest in providing quality product support. The legal ground for signing in to your account, if appropriate, to help troubleshoot and resolve your issue is your consent, which you may withdraw.

Personal data that is processed when you purchase a product, service, or subscription:

If you purchase a product, service, or subscription on a Garmin website or app or through customer support, then we will collect your name, address, email address, and telephone number and other information needed for billing in your jurisdiction. We do not view or store your payment card information, which is handled by third-party service providers.

PURPOSES AND LEGAL GROUNDS:

We collect your name, mailing address, and telephone number so we can process your order and fulfill your purchase. The legal ground for processing your name, mailing address, and telephone number for these purposes is performance of a contract. We also process your personal data as part of our fraud detection processes, which may include the use of personal data related to the purchase, delivery, or use of products, services, or subscriptions as needed to investigate requests or claims. The legal ground for processing your personal data for that purpose is our legitimate interest in protecting us and our customers from attempts to engage in fraudulent transactions. If you reside in mainland China and provide your opt-in consent to receiving marketing information from us, we will also process your telephone number to send you marketing communications.

Personal data that is processed when you purchase a product in a Garmin store:

When you purchase a product in a Garmin store, we may collect your name, address, telephone number, other contact information, or purchase details, such as invoice number or products purchased.

PURPOSES AND LEGAL GROUNDS:

We process this information to generate an invoice in our systems for tax reporting purposes or upon your request. The legal ground for processing your information for generation of an invoice for tax reporting purposes is fulfillment of a legal obligation. The legal ground for processing your information for generation of an invoice at your request

is performance of a contract.

Personal data that is processed when you use our live location-based services:

If you choose to use location-based services, such as weather, on your device or app, then the physical location of your device will be collected in order for us or our providers to provide you with such location-based services.

PURPOSE AND LEGAL GROUND:

The purpose of processing the location of your device is to provide you the location-based services you wish to use. The legal ground for processing this data for this purpose is legitimate interest.

Personal data that is processed when you use your Garmin auto navigation device or app:

If you use a Garmin auto navigation device or app and provide your consent, then we will collect and upload from your device data such as location, speed, direction, and time and date of recording. If you provide your consent when asked, then we may also share this aggregated data with third parties to enhance the quality of the traffic, parking, and other features enabled by content providers.

PURPOSE AND LEGAL GROUND:

This data is aggregated with data from other consenting users and is used to enhance the quality of our products, services, and apps and the traffic, parking, and other features enabled by us or third parties. The legal ground for processing this data for this purpose is your consent, which you may withdraw at any time within the settings of your Garmin device or app.

Personal data that is processed to address fraud and abuse:

We take steps to detect and mitigate fraud and abuse on our services, websites, and apps. If we suspect a fraudulent request or claim has been made relating to your account or device, we may process data relating to the device(s) associated with your account, basic account information such as email address, name, phone number(s), and mailing address(es), or our communication history with you in order to adequately investigate the request or claim. We may process content that you have contributed to your account, such as photos, comments, and reviews, to enforce our terms of use and to identify and remove inappropriate, harmful, or abusive content.

PURPOSE AND LEGAL GROUND:

We perform these processing activities to protect the interests of Garmin, its customers, and other stakeholders against fraud, breach of contract, and other harmful or unlawful actions. The legal ground for this processing is our legitimate interest in reducing fraud and abuse perpetuated through the use of, or in connection with, our products and services.

Categories of Recipients of Personal Data

SERVICE PROVIDERS:

We use cloud services from third parties such as Adobe and SendGrid, a Twilio service, to assist in sending emails. Those services track the activities associated with these emails, such as whether they were opened, whether links in the emails were clicked on, and whether purchases were made following clicks on those links. We use this data to analyze the level of engagement with our emails.

We use services from third parties such as Vonage and Twilio to send SMS messages to our users or their contacts in support of features described in this Privacy Policy. We may track the delivery of these messages based on the phone number of the recipient and the selected content. We use other SMS vendors in some regions, such as Alibaba Cloud (mainland China only), every8d (Taiwan only), aligo sms (South Korea only), fpt / chunghwa (Vietnam only) and true corp (Thailand only).

We use Adyen as a third-party payment processor. More information about Adyen can be found here. Depending on your region and product purchased, additional third-party payment processors or payment methods are available for you to select, such as PayPal, Alipay, WeChat Pay powered by tenpay, and UnionPay. We also use Cybersource, a solution provided by Visa, in our fraud detection processes, address verification services provided by third parties such as loqate, Google, or CDYNE, and subscription billing and payment services from Avalara and LogiSense.

We use third-party service providers to ship purchased products to customers. In some regions, we use a third party, Erply, to provide point-of-sale services for in-store purchases. In some regions, we use a third party, Narvar, to allow you to view shipment information and track items purchased from our website or purchased, repaired, or exchanged through customer support. In some regions, we use a third party, Global Blue, to provide tax rebate paperwork for customers upon request. We also use a third-party service provider, Medallia, to provide callback request functionality for customer support purposes. In some countries, third-party providers may be used to generate the reporting required for the appropriate tax regulatory agency.

We use third-party service providers to help us better understand the usage and performance of our products, services, websites, and apps. See the "Monitoring and Analytics" section for more information.

OTHER DISCLOSURES:

We may process and disclose personal data about you to others: (a) if we have your valid consent to do so; (b) to comply with legal and regulatory obligations, such as a valid subpoena, court or judicial order, other valid legal process, or record keeping to support

applicable reporting and auditing requirements; (c) to investigate potential fraud and enforce any of our terms and conditions or policies; (d) as necessary to pursue available legal remedies or defend legal claims.

We may also transfer your personal data to an affiliate, a subsidiary, or a third party in the event of any reorganization, merger, sale, joint venture, assignment, transfer, or other disposition of all or any portion of our business, assets, or stock, including, without limitation, in connection with any bankruptcy or similar proceeding, provided that any such entity that we transfer personal data will not be permitted to process your personal data other than as described in this Privacy Policy without providing you notice and, if required by applicable laws, obtaining your consent.

International Transfers of Personal Data

Garmin is a global business. To offer our products, apps, and services, we may need to transfer your personal data to Garmin-owned companies in other countries. View Garmin-owned companies.

When you create a Garmin account, add personal data in your account profile, or upload data to your Garmin account, your personal data will be collected and stored on servers in the U.S., U.K., and/or Australia.

For customers in mainland China, when you create a Garmin account, add personal data in your account profile, or upload data to your Garmin account, your personal data will be collected and stored on Garmin China Shanghai RHQ Co., Ltd.'s servers in mainland China.

Personal data regarding individuals who reside in a country in the European Economic Area ("EEA"), United Kingdom ("U.K."), or Switzerland is controlled by Garmin Würzburg GmbH and processed on its behalf by other Garmin-owned companies to provide support, infrastructure, security, and other key functions. Garmin Würzburg GmbH has entered into approved model contractual clauses where these companies are not located in the EEA or a jurisdiction deemed adequate under applicable data protection law. Garmin's U.S.-based affiliates are certified under the EU-U.S. and Swiss-U.S. Data Privacy Frameworks. View our Data Privacy Framework Notice.

All Garmin-owned companies are required to follow the privacy practices set forth in this Privacy Policy.

Cookies and Similar Technologies

Information about Garmin's use of cookies and similar technologies on our websites can be found in our Cookie Policy.

Monitoring and Analytics

We collect data from users about their usage of our products, services, websites, and apps. The types of analytical information that are collected include the date and time of access to our servers, software or firmware version, the location of the device, language setting, what information and files have been downloaded, user behavior (e.g., features used, frequency of use), device state information, device model, hardware and operating system information, and information relating to how the product, service, website, or app functions. Garmin uses this data to improve the quality, security, and functionality of our products, services, websites, or apps; to develop and market products and features that best serve you and other users; and to help identify and fix stability issues and other usability problems as quickly as possible.

The legal ground for processing this analytical information is our legitimate interest in understanding how our customers interact with our products, services, websites, and apps so we can enhance user experience and functionality.

Here are examples of third-party providers we currently use:

- **Google:** Google Analytics is used to track statistics and user demographics, interests, and behavior. Find out more information about how this analytics information may be used, how to control the use of your information, and how to opt out of having your data used by Google Analytics.
- **Azure Application Insights:** Azure Application Insights, a Microsoft service, is used to help us better understand usage to improve user experience.
- **Microsoft App Center; Firebase (Crashlytics):** Microsoft App Center, formerly known as HockeyApp, and Firebase (Crashlytics), a Google service, are used to help us better

understand usage to improve user experience and to identify and resolve the root causes of crashes or errors.

- **Flurry; Amplitude:** Flurry and Amplitude are used to help us better understand usage to improve user experience.

See the "Cookies and Similar Technologies" section for information on Monitoring and Analytics vendors that rely on cookies.

SOCIAL NETWORKS:

Third-party social networks that provide social networking features (e.g., to allow you to connect to social media, such as Facebook, Google, QQ, or Sina Weibo, to find friends to add as connections or to "Like" a page) in Garmin products, services, websites, or apps may gather information regarding your use of our products, services, websites, or apps. The use of such information by a third party depends on the privacy policy available on that social network's website, which we encourage you to carefully review. Such third parties may use these methods and this information for their own purposes by relating information about your use of our products, services, websites, or apps with any of your personal information that they may have. We may also obtain analytics information from social networks that help us measure the effectiveness of our content and advertisements on social networks (e.g., impressions and clicks).

Children

We request individuals under the age of 13 in the U.S. and under the age of 16 in the rest of the world not provide personal data to Garmin. If we learn that we have collected personal data from a child under the age of 13 in the U.S. or under 16 in the rest of the world, we will take steps to delete the information as soon as possible.

Privacy Policy Updates

We may update this Privacy Policy from time to time as we add new products, services, and apps, as we improve our current offerings, and as technologies and laws change. You can determine when this Privacy Policy was last revised by referring to the “Last Updated” legend at the top of this page. Any changes will become effective upon our posting of the revised Privacy Policy.

We will provide notice to you if these changes are material and, where required by applicable law, we will obtain your consent. This notice will be provided by email or by posting notice of the changes on the Garmin websites and apps that link to this Privacy Policy.

Retention of Personal Data

We will retain your personal data as long as your Garmin account is considered to be active or in accordance with applicable law and regulatory obligations. In addition, see below under “Manage Your Data” for information on how to delete your data or account.

Automated Decision-Making, Including Profiling

We do not make any decisions based on algorithms or other automated processing that significantly affect you.

Data Controller and Data Protection Officer

If you reside in a country in the EEA, U.K., or Switzerland, then your personal data collected by Garmin is controlled by Garmin Würzburg GmbH, Beethovenstr. 1a, 97080 Würzburg, Germany. The company's EU Data Protection Officer can be reached by email at euprivacy@garmin.com.

If you reside in mainland China, then your personal data collected by Garmin is controlled by Garmin China Shanghai RHQ Co., Ltd., 37F, Tower A, New CHJ International Business Center, No 391, Guiping Road, Xuhui District, Shanghai 200233, China. The company's Data Protection Officer for mainland China can be reached at the same address and by email at cnprivacy@garmin.com.

If you reside outside of the EEA, U.K., Switzerland, and mainland China, then your personal data collected by Garmin is controlled by Garmin International, Inc., 1200 E. 151st St., Olathe, Kansas 66062, USA, which you can contact by email at privacy@garmin.com. The company's Brazilian Data Protection Officer is located at the same address and can be reached by email at privacy@garmin.com. The company's Turkish Representative can be reached by email at trprivacy@garmin.com.

Manage Your Data

We provide a self-service portal, the Account Management Center, to allow you to access, export, correct, or delete your data at any time. The Account Management Center requires you to sign in with your Garmin credentials to ensure that only you can manage your data and exercise your rights.

If you do not have an account or otherwise want to learn more about your rights based on where you reside, please visit Your Data Protection Rights.

All trademarks are the property of their respective owners.

Recent Policy Versions

- May 10, 2022
- April 29, 2021
- June 5, 2020

CUSTOMER SERVICE

COMPANY

PLATFORMS

FOR BUSINESS

United States

Site Map Terms of Use Privacy Security Web Accessibility

© Garmin Ltd. or its subsidiaries. All rights reserved.

Cookie-Präferenzen

PRIVACY POLICY FOR GARMIN CONNECT AND COMPATIBLE GARMIN DEVICES

Last Updated: September 21, 2023

Your privacy is important to Garmin. We developed this Privacy Policy to provide you with information on how we process your personal data when you use the Garmin Connect website and mobile app and Garmin wearable devices or other Garmin devices that are compatible with Garmin Connect.

Other Garmin websites, services, apps, and products that include a link to a different Garmin privacy policy are governed by that privacy policy. Click here to review our other privacy policies.

"Personal data" is information relating to an identified or identifiable natural person. Additional information specific to your jurisdiction may be provided in a separate document. Please see our supplemental policy, "Your Data Protection Rights," for additional information that may apply to you. The legal grounds for processing personal data specified in this Privacy Policy are based on the EU General Data Protection Regulation ("GDPR") and laws in other jurisdictions that have similar grounds. If you are a California resident, please see our CCPA Privacy Notice.

To jump to a specific section of this Privacy Policy, please click on a link below:

- Categories of Personal Data Processed by Garmin
- Categories of Recipients of Personal Data

- International Transfers of Personal Data
- Cookies and Similar Technologies
- Monitoring and Analytics
- Children
- Privacy Policy Updates
- Retention of Personal Data
- Automated Decision-Making, Including Profiling
- Data Controller and Data Protection Officer
- Manage Your Data
- Recent Policy Versions

Categories of Personal Data Processed by Garmin

Personal data that is processed when you create a Garmin account:

When you create an account, we ask you to provide your email address, name, and password. You can choose to provide only your first name or a nickname instead of your full name if you wish. We may also ask you to provide your mobile phone number.

PURPOSES AND LEGAL GROUNDS:

(a) We process your email address and password because you use your email address and password to sign in to your account. The legal ground for processing your email address and password for this purpose is our legitimate interest in protecting the security of your account. If you enable two-factor authentication, we process your mobile phone number or email address to send the security code via SMS or email. The legal ground for processing this information for this purpose is our legitimate interest in protecting the security of your account. If you reside in mainland China, we process your mobile phone

number for purposes of real name authentication.

(b) We also process your email address for the purpose of sending you important information about your Garmin products, services, apps, or account, such as important safety information or material changes to this Privacy Policy. The name you provide is associated with your account profile and is displayed when you submit comments or other material on our websites or apps or engage with other users. The legal ground for processing your email address and name for these purposes is our legitimate interest in providing you important safety or other information about your Garmin products, services, apps, or account or material changes to this Privacy Policy and in providing you an opportunity to engage with other users.

(c) If you provide your opt-in consent to receiving marketing information from us, we will also process your email address for the purpose of sending you marketing information about our products, services, and apps, as well as newsletters. The legal ground for processing your email address for this purpose is your consent. You may withdraw your consent at any time by changing your preferences in your account or through the unsubscribe link at the bottom of our marketing emails. The marketing emails you receive from us are based on the preferences you provide in your account, the locale indicated by your Internet Protocol (IP) address, the types of devices you have added to your account, and any subscriptions included in your account. The legal ground for processing this data for this purpose is our legitimate interest in reducing the number of marketing emails sent to each particular customer by selecting which customers receive a particular marketing email rather than sending every marketing email to every customer who has consented to receiving marketing emails. If you reside in mainland China, we may use your mobile phone number to send you marketing communications via SMS.

(d) We also process your email address to associate it with your account when you interact with our customer support representatives. The legal ground for this processing is our legitimate interest in providing quality customer support.

(e) We also process your email address to notify customers when they have violated our terms. The legal ground for this processing is our legitimate interest in ensuring a quality experience for all customers and ensuring adherence to our terms.

Personal data that is processed when you sign in to your Garmin account with social media credentials if you choose (mainland China only):

If you live in mainland China, you may choose to sign in to your account using your social media sign-in credentials (e.g., your WeChat, QQ, or Apple sign-in credentials). If you choose this method, the first time you sign in, you will be asked whether you agree to the social media provider delivering certain information to us, such as your identifier, nickname, email address, profile photo, and other information associated with your social media account.

All of this information is made available to us by the social media provider due to the way the social sign-on configuration works. However, the only information we retain and process is

your email address. If you do not want your information to be shared with us by the social media provider, then you can simply sign in to your account using your account credentials instead of your social media account credentials.

PURPOSE AND LEGAL GROUND:

We associate the email address provided by the social media provider with your account so you can use your email address to sign in to your account in the future if you no longer wish to sign in using your social media account credentials. The legal ground for processing this information for this purpose is our legitimate interest in providing you an alternative sign-in method and in securing your account.

Additional personal data that is processed if you choose to provide it when you create a profile:

You can add additional information to your profile, such as your location, types of activities you participate in (e.g., running, hiking, cycling, golf, etc.), and a photo, and your gender, birthdate, height, and weight.

PURPOSE AND LEGAL GROUND:

This information is used to calculate the calories you burn during an activity and to enable you to show your connections the types of activities you participate in if you wish. The legal ground for processing this information for these purposes is your consent. You can withdraw your consent by deleting this information from your account profile.

Personal data that is processed when you choose to upload or add your data to your Garmin account:

You can choose to upload from your device or, in some cases, manually add activities (e.g., runs, walks, bike rides, swims, hikes, gym activities, etc.) and activity data (e.g., steps, distance, pace, activity time, calories burned, heart rate, sleep, location, golf stats, menstrual cycle information, hydration, music played, etc.) to your account. You can use your device without providing your consent to upload your activities to your account. If you choose to add your activities to your account, you control whether others can see your activity data by managing the privacy settings in your account. Your activity data is set to "Private" by default.

PURPOSES AND LEGAL GROUNDS:

(a) We process this data, if you choose to upload or add it to your account, to enable you to analyze this data, identify any devices used to collect data during the activity, see your location on your activity course and segment maps, see your heart rate related metrics such as stress score, Body Battery energy monitoring, or pulse oximetry, track your fitness goals, and, if you wish, share this data with others. If you reside in the EEA, U.K., or Switzerland, the legal ground for this processing is your explicit consent, which you can withdraw at any time within your account.

(b) If you choose to upload or add this data to your account and you choose to

participate in insights, then you will be presented with an insights section in your account in which you will be provided with recommendations and motivational messages, information, and links to articles that may be of interest to you based upon this data, and a comparison of this data with aggregated activity data of others in the Garmin Connect community. If you reside in the EEA, U.K., or Switzerland, the legal ground for processing this data for this purpose is your explicit consent, which you can withdraw at any time within your account.

(c) We also process this data, if you choose to upload or add it to your account, in an aggregated manner to analyze usage and trends and develop or improve features and services. The legal ground for this processing is our legitimate interest in providing relevant and quality features and services.

(d) If you choose to upload or add this data to your account and you are opted in to product improvement, we will process this data for research and development purposes internally to help us build better and more relevant products and services. If you reside in the EEA, U.K., or Switzerland, the legal ground for processing this data for this purpose is your explicit consent, which you can withdraw at any time within your account.

Personal data that is processed when you add a Garmin device to your Garmin account:

When you add certain devices to your account, we collect device information such as device identifiers. We also ask you to provide additional information, such as your gender, height, weight, birthdate, activity level (low, medium, or high), and normal bed and wake times.

PURPOSE AND LEGAL GROUND:

We process your device information to associate your device to your account. We process the additional information you provide to calculate the calories you burn during an activity, provide you insights about your activities and sleep (if you give your explicit consent to be presented with insights), and to set your wearable device to “do not disturb” mode during your normal sleep hours. The legal ground for processing this information for these purposes is your consent. You can withdraw your consent at any time by removing your device from your account and deleting this information from your profile.

Personal data that is processed when you enable email notifications:

If you enable email notifications, we process your email address in accordance with your notification settings.

PURPOSE AND LEGAL GROUND:

If you provide your opt-in consent, we will process your email address for the purpose of sending you notifications based on your selections (e.g., when you join a challenge, when a challenge is over, when comments to a challenge are submitted, etc.). The legal ground for processing your email address for this purpose is your consent. You can withdraw this consent at any time by

changing your email settings to opt out of receiving such notifications or by disabling email notifications.

Personal data that is processed when you sync your Garmin device:

When you sync your device through the Garmin Connect website or mobile app or Garmin Express, we log data about the transmission, such as the IP address used when syncing, the sync time and date, crash/diagnostic logs, geographic location of the device, information about your device, information about the network used to sync (e.g., Wi-Fi or cellular), and device battery level. For compatible products, we will also gather analytics on how you configure and use certain features (e.g., Garmin Pay, Connect IQ, music features, etc.).

PURPOSES AND LEGAL GROUNDS:

We process this information to help identify and resolve errors or syncing issues. The legal ground for processing this information for this purpose is our legitimate interest in resolving errors or syncing issues and providing quality product support. We also process this information to analyze usage and trends and develop or improve features and services. The legal ground for this processing is our legitimate interest in providing relevant and quality features and services.

Personal data that is processed when you connect your Garmin account to a third-party app if you choose:

If you choose to enable your account to access accounts you have with other app providers, such as your MyFitnessPal, Strava, or TrainingPeaks account, we will obtain information about you from such account, such as the number of calories consumed in a particular day based on information from your MyFitnessPal account or courses and segments from your Strava account.

PURPOSE AND LEGAL GROUND:

This information is used to supplement your activity information in Garmin Connect with information you have provided to such third-party apps. The legal ground for this processing is your consent. You can withdraw your consent at any time by disconnecting your account from the third-party app within your account.

Personal data that is processed when you communicate with Garmin:

When you interact with our customer support representatives by email or telephone, online, or in person, we collect personal data, such as your name, mailing address, phone number, email address, and contact preferences, and information about the products you own, such as their serial numbers and dates of purchase, and subscriptions you've purchased. We also may create event logs that are useful in diagnosing product or app performance-related issues and capture information relating to the support or service issue. To improve customer service, subject to applicable laws, we may also record and review conversations with customer support representatives and analyze any feedback provided to us through voluntary customer surveys. With your consent, our customer support representatives may sign in to your account, if appropriate, to help troubleshoot and resolve your issue.

PURPOSES AND LEGAL GROUNDS:

We use this information to provide you with customer and product support, to monitor the quality and types of customer and product support we provide to our customers, and to facilitate repairs, returns, or exchanges. The legal ground for processing this information for these purposes is our legitimate interest in providing quality product support. The legal ground for signing in to your account, if appropriate, to help troubleshoot and resolve your issue is your consent, which you may withdraw.

Personal data that is processed when you use location-based services:

If you choose to use location-based services, such as weather, on your device or app, then the physical location of your device will be collected in order for us or our providers to provide you with such location-based services.

PURPOSE AND LEGAL GROUND:

The purpose of processing the location of your device is to provide you the location-based services you wish to use. The legal ground for processing this data for this purpose is performance of a contract.

Personal data that is processed when you are a real-time tracking (e.g., LiveTrack) invitee or emergency contact on someone's Garmin account:

If a Garmin user sends you an invitation to track their location or activity in real time (e.g., via the LiveTrack feature) or adds you as an emergency contact to their Garmin account, they provide us your phone number or email address.

PURPOSE AND LEGAL GROUND:

We use this information to send you the real-time tracking information or to notify you of an incident involving the Garmin user. The legal ground for processing this information for these purposes is our legitimate interest in enabling our users to send real-time tracking information to their friends and family, and in enabling our users to notify their emergency contacts if they are involved in an incident and need assistance.

Personal data that is processed when you use the Assistance Plus service:

When you use the Assistance Plus service, we process your email address, mailing address, telephone number, and contact information for your chosen emergency contacts. We also process text messages sent to and from your device and the location of your device when your device communicates with the Garmin Response team.

PURPOSES AND LEGAL GROUNDS:

(a) We process text messages sent to and from your device and the location of your device when your device communicates with Garmin Response in order for Garmin and its service providers to perform the Assistance Plus service. The legal ground for this processing is performance of a contract.

(b) We process your mailing address and telephone number to determine if Garmin Response has been activated inadvertently by a subscriber at their residence. The legal ground for this processing is our legitimate interest in minimizing the number of false alarms.

Personal data that is processed to address fraud and abuse:

We take steps to detect and mitigate fraud and abuse on our services, websites, and apps. If we suspect a fraudulent request or claim has been made relating to your account or device, we may process data relating to the device(s) associated with your account, basic account information such as email address, name, phone number(s), and mailing address(es), or our communication history with you in order to adequately investigate the request or claim. We may process content that you have contributed to your account, such as photos, comments, and reviews, to enforce our terms of use and to identify and remove inappropriate, harmful, or abusive content.

PURPOSE AND LEGAL GROUND:

We perform these processing activities to protect the interests of Garmin, its customers, and other stakeholders against fraud, breach of contract, and other harmful or unlawful actions. The legal ground for this processing is our legitimate interest in reducing fraud and abuse perpetuated through the use of, or in connection with, our products and services.

Personal data that is processed when you use the Garmin Pay™ contactless payment solution:

When providing the Garmin Pay service, we process personal data in accordance with this privacy policy. For specific information on our processing of personal data for Garmin Pay, including the categories of personal data we process, the purposes and legal grounds of processing, and the categories of recipients of personal data, please click here .

Categories of Recipients of Personal Data

OTHER GARMIN CONNECT USERS:

Your activities and activity data associated with your Garmin account are set to "Private" by default. You may decide to allow others to view your activities and activity data by changing the privacy settings in your Garmin account. You can join groups and make connections with other Garmin Connect users. You may also choose to join other Garmin Connect users in

challenges and leaderboards. When you interact with others in these ways, you will be displaying your data relating to the challenge or leaderboard (e.g., aggregate number of steps during the duration of a steps leaderboard or challenge) even if your privacy settings in your Garmin account are set to "Private."

FAMILY MEMBERS OF GARMIN JR. CHALLENGE PARTICIPANTS:

When you choose to participate in challenges that include participants who are part of a Garmin Jr. family account, challenge participants and anyone in their family account may view your data relating to the challenge and its leaderboard, even if they are not participating in the challenge and even if your privacy settings in your Garmin account are set to "Private."

YOUR REAL-TIME TRACKING INVITEES:

Some Garmin devices include features, such as LiveTrack, that enable you to send a link to people of your choice that allows them to see the real-time location of your device. Because anyone with access to the link will be able to see the real-time location of your Garmin device, you should use caution in determining to whom you want to send the link and be sure that you trust them to not send the link to others whom you do not want to be able to view the location of your Garmin device.

YOUR EMERGENCY CONTACTS:

If you provide emergency contacts to us to be used in the event that an incident is detected or reported and you are involved in an incident that is detected by your Garmin device, we will send a notification with information about the incident, such as location, to your chosen emergency contacts via SMS or email.

GARMIN RESPONSE, SEARCH AND RESCUE AUTHORITIES, AND EMERGENCY RESPONDERS:

In connection with your use of the Assistance Plus service, Garmin, through Garmin Response , may provide information, such as your name, email address, mailing address, telephone number, the message content, time sent, and the identity and location of the message sender and recipient, to competent search and rescue authorities and emergency responders.

THIRD-PARTY APP, PLATFORM, OR SERVICE PROVIDERS WITH WHOM YOU ASK GARMIN TO SHARE YOUR DATA:

If you choose to authorize us to permit a third party, such as your wellness program provider, or an app, such as MyFitnessPal, Strava, or TrainingPeaks, to access your activity data in your Garmin account, then we will share such data with the third party. We will not do this without your explicit consent. Once you direct us to share data with a third party, the third party's handling of your personal data is the responsibility of that third party, and you should carefully review the third party's privacy policy. You can choose to stop sharing data with the third-party app, platform, or service provider at any time within your Garmin account.

OTHER SERVICE PROVIDERS:

We use cloud services from third parties such as Adobe and SendGrid, a Twilio service, to assist in sending emails. Those services track the activities associated with these emails, such as whether they were opened, whether links in the emails were clicked on, and whether purchases were made following clicks on those links. We use this data to analyze the level of engagement with our emails.

We use services from third parties such as Vonage and Twilio to send SMS messages to our users or their contacts in support of features described in this Privacy Policy. We may track the delivery of these messages based on the phone number of the recipient and the selected content. We use other SMS vendors in some regions, such as Alibaba Cloud (mainland China only).

We use third-party service providers to help us better understand the usage and performance of our products, services, websites, and apps. See the "Monitoring and Analytics" section for more information.

We use Amazon Web Services (AWS) to host photos.

We also use services from third parties such as Alibaba Cloud (mainland China only) to help identify inappropriate or harmful content uploaded by users. We provide personal data to such services only if included in the user's uploaded content.

OTHER DISCLOSURES:

We may process and disclose personal data about you to others: (a) if we have your valid consent to do so; (b) to comply with legal and regulatory obligations, such as a valid subpoena, court or judicial order, other valid legal process, or record keeping to support applicable reporting and auditing requirements; (c) to investigate potential fraud and enforce any of our terms and conditions or policies; (d) as necessary to pursue available legal remedies or defend legal claims; or (e) as we deem necessary or appropriate for purposes of attempting to get you help in the event you are involved in an emergency situation.

We may also transfer your personal data to an affiliate, a subsidiary, or a third party in the event of any reorganization, merger, sale, joint venture, assignment, transfer, or other disposition of all or any portion of our business, assets, or stock, including, without limitation, in connection with any bankruptcy or similar proceeding, provided that any such entity that we transfer personal data to will not be permitted to process your personal data other than as described in this Privacy Policy without providing you notice and, if required by applicable laws, obtaining your consent.

International Transfers of Personal Data

Garmin is a global business. To offer our products, apps, and services, we may need to transfer your personal data to Garmin-owned companies in other countries. View Garmin-owned companies.

When you create a Garmin account, add personal data in your account profile, or upload data to your Garmin account, your personal data will be collected and stored on servers in the U.S., U.K., and/or Australia.

For customers in mainland China, when you create a Garmin account, add personal data in your account profile, or upload data to your Garmin account, your personal data will be collected and stored on Garmin China Shanghai RHQ Co., Ltd.'s servers in mainland China.

Personal data regarding individuals who reside in a country in the European Economic Area ("EEA"), United Kingdom ("U.K."), or Switzerland is controlled by Garmin Würzburg GmbH and processed on its behalf by other Garmin-owned companies to provide support, infrastructure, security, and other key functions. Garmin Würzburg GmbH has entered into approved model contractual clauses where these companies are not located in the EEA or a jurisdiction deemed adequate under applicable data protection law. Garmin's U.S.-based affiliates are certified under the EU-U.S. and Swiss-U.S. Data Privacy Frameworks. View our Data Privacy Framework Notice.

All Garmin-owned companies are required to follow the privacy practices set forth in this Privacy Policy.

Cookies and Similar Technologies

Information about Garmin's use of cookies and similar technologies on our websites can be found in our Cookie Policy.

Monitoring and Analytics

We collect data from users about their usage of our products, services, websites, and apps. The types of analytical information that are collected include the date and time of access to our servers, software or firmware version, the location of the device, language setting, what information and files have been downloaded, user behavior (e.g., features used, frequency of use), device state information, device model, hardware and operating system information, and information relating to how the product, service, website, or app functions. Garmin uses this data to improve the quality, security, and functionality of our products, services, websites, or apps; to develop and market products and features that best serve you and other users; and to help identify and fix stability issues and other usability problems as quickly as possible.

The legal ground for processing this analytical information is our legitimate interest in understanding how our customers interact with our products, services, websites, and apps so we can enhance user experience and functionality.

Here are examples of third-party providers we currently use:

- **Google:** Google Analytics is used to track statistics and user demographics, interests, and behavior. Find out more information about how this analytics information may be used, how to control the use of your information, and how to opt out of having your data used by Google Analytics.
- **Azure Application Insights:** Azure Application Insights, a Microsoft service, is used to help us better understand usage to improve user experience.
- **Microsoft App Center; Firebase (Crashlytics):** Microsoft App Center, formerly known as HockeyApp, and Firebase (Crashlytics), a Google service, are used to help us better understand usage to improve user experience and to identify and resolve the root causes of crashes or errors.

See the "Cookies and Similar Technologies" section for information on Monitoring and Analytics vendors that rely on cookies.

Children

We request individuals under the age of 13 in the U.S. and under the age of 16 in the rest of the world not provide personal data to Garmin. If we learn that we have collected personal data from a child under the age of 13 in the U.S. or under 16 in the rest of the world, we will take steps to delete the information as soon as possible.

Privacy Policy Updates

We may update this Privacy Policy from time to time as we add new products, services, and apps, as we improve our current offerings, and as technologies and laws change. You can determine when this Privacy Policy was last revised by referring to the “Last Updated” legend at the top of this page. Any changes will become effective upon our posting of the revised Privacy Policy.

We will provide notice to you if these changes are material and, where required by applicable law, we will obtain your consent. This notice will be provided by email or by posting notice of the changes on the Garmin websites and apps that link to this Privacy Policy.

Retention of Personal Data

We will retain your personal data as long as your Garmin account is considered to be active or in accordance with applicable law and regulatory obligations. In addition, see below under “Manage Your Data” for information on how to delete your data or account.

Automated Decision-Making, Including Profiling

We do not make any decisions based on algorithms or other automated processing that

significantly affect you.

Data Controller and Data Protection Officer

If you reside in a country in the EEA, U.K., or Switzerland, then your personal data collected by Garmin is controlled by Garmin Würzburg GmbH, Beethovenstr. 1a, 97080 Würzburg, Germany. The company's EU Data Protection Officer can be reached by email at euprivacy@garmin.com.

If you reside in mainland China, then your personal data collected by Garmin is controlled by Garmin China Shanghai RHQ Co., Ltd., 37F, Tower A, New CHJ International Business Center, No 391, Guiping Road, Xuhui District, Shanghai 200233, China. The company's Data Protection Officer for mainland China can be reached at the same address and by email at cnprivacy@garmin.com.

If you reside outside of the EEA, U.K., Switzerland, and mainland China, then your personal data collected by Garmin is controlled by Garmin International, Inc., 1200 E. 151st St., Olathe, Kansas 66062, USA, which you can contact by email at privacy@garmin.com. The company's Brazilian Data Protection Officer is located at the same address and can be reached by email at privacy@garmin.com. The company's Turkish Representative can be reached by email at trprivacy@garmin.com.

Manage Your Data

We provide a self-service portal, the Account Management Center, to allow you to access, export, correct, or delete your data at any time. The Account Management Center requires you to sign in with your Garmin credentials to ensure that only you can manage your data and exercise your rights.

If you do not have an account or otherwise want to learn more about your rights based on

where you reside, please visit Your Data Protection Rights.

All trademarks are the property of their respective owners.

Recent Policy Versions

- August 31, 2022
- April 29, 2021
- June 5, 2020

CUSTOMER SERVICE

COMPANY

PLATFORMS

FOR BUSINESS

United States

Site Map Terms of Use Privacy Security Web Accessibility

© Garmin Ltd. or its subsidiaries. All rights reserved.

Cookie-Präferenzen

GARMIN

0

FREE GROUND SHIPPING ON ORDERS $25 AND UP

PRIVACY POLICY FOR GARMIN JR. APP

Last Updated: August 22, 2023

Your privacy is important to Garmin. We developed this Privacy Policy to provide you with information on how we collect, use, process, and disclose personal data when you and your children use the Garmin Jr. application ("Garmin Jr. app"), formerly known as the vívofit jr. app.

Other Garmin websites, services, apps, and products that include a link to a different Garmin privacy policy are governed by that privacy policy. Click here to review our other privacy policies.

"Personal data" is information relating to an identified or identifiable natural person. It includes "personal information" as that term is defined under the U.S. Children's Online Privacy Protection Act (COPPA).

Additional information specific to your jurisdiction may be provided separately, such as additional documents linked to this Privacy Policy or notices provided in the Garmin Jr. app when obtaining parental consent. Please scroll to the "Manage Your Family Data" section to view our supplemental policies that may apply to you. The legal grounds for processing personal data specified in this Privacy Policy are based on the EU General Data Protection Regulation ("GDPR") and laws in other jurisdictions that have similar grounds. If you are a California resident, please see our CCPA Privacy Notice.

To jump to a specific section of this Privacy Policy, please click on a link below:

- Categories of Personal Data Collected and Processed by Garmin

- Categories of Persons to Whom Personal Data Is Disclosed
- International Transfers of Personal Data
- Monitoring and Analytics
- Privacy Policy Updates
- Retention of Personal Data
- Automated Decision-Making, Including Profiling
- Data Controller and Data Protection Officer
- Manage Your Family Data
- Recent Policy Versions

Categories of Personal Data Collected and Processed by Garmin

Personal data that is collected and processed when you, as the parent, create a Garmin account:

When you, as the parent, create a Garmin account, we ask you to provide your email address, name, and password. You can choose to provide only your first name or a nickname instead of your full name if you wish. We may also ask you to provide your mobile phone number.

PURPOSES AND LEGAL GROUNDS:

(a) Garmin collects and processes your email address and password because you use your email address and password to sign in to your account. The legal ground for processing your email address and password for this purpose is Garmin's legitimate interest in protecting the security of your account. If you enable two-factor authentication, Garmin processes your mobile phone number or email address to send the security code via SMS or email. The legal ground for processing this information for this purpose is Garmin's legitimate interest in

protecting the security of your account. If you reside in mainland China, Garmin processes your mobile phone number for purposes of real name authentication.

(b) Garmin also collects and processes your email address for the purpose of sending you important information about your Garmin products, services, apps, or account, such as important safety information or material changes to this Privacy Policy. The name you provide is associated with your account profile and is displayed when you submit comments or other material on a Garmin website or app or engage with other Garmin users. The legal ground for processing your email address and name for these purposes is Garmin's legitimate interest in providing you important safety or other information about your Garmin products, service, apps, or account or material changes to this Privacy Policy and in providing you an opportunity to engage with other Garmin users.

(c) If you provide your opt-in consent to receiving marketing information from Garmin, Garmin will also collect and process your email address for the purpose of sending you marketing information about Garmin products, services, and apps, as well as newsletters. The legal ground for processing your email address for this purpose is your consent. You may withdraw your consent at any time by changing your preferences in your Garmin account or through the unsubscribe link at the bottom of our marketing emails. The marketing emails you receive from Garmin are based on the preferences you provide in your Garmin account, the locale indicated by your Internet Protocol (IP) address, the types of Garmin devices you have added to your Garmin account, and any subscriptions included in your Garmin account. The legal ground for processing this data for this purpose is Garmin's legitimate interest in reducing the number of marketing emails sent to each particular customer by selecting which customers receive a particular marketing email rather than sending every marketing email to every customer who has consented to receiving marketing emails. If you reside in mainland China, Garmin may use your mobile phone number to send you marketing communications via SMS.

(d) Garmin also collects and processes your email address to associate it with your Garmin account when you interact with our customer support representatives. The legal ground for this processing is our legitimate interest in providing quality customer support.

(e) Garmin also collects and processes your email address to notify customers when they have violated our terms. The legal ground for this processing is our legitimate interest in ensuring a quality experience for all customers and ensuring adherence to our terms.

Additional personal data that is collected and processed when you download the Garmin Jr. app and create your family profile:

When you create your family profile in the Garmin Jr. app, your Garmin Connect name will be used as your admin name in the Garmin Jr. app. You also will be asked to provide a name for your family and a name for each of your children whose compatible devices you add to your profile. You do not need to give your family's actual name or your children's full names or even your children's actual names in the "name" fields. There will be a default wake and bed

time for each of your children whose compatible devices you add to your profile. You can, but are not required to, change the default wake and bed times. In addition, you can, but are not required to, type in a location for your family (which can be anything you choose, such as a country, state, city, etc.) and your children's birthdays and genders. You can leave those fields blank if you wish.

You will be asked to provide a display text for each of your children whose compatible devices you add to your profile. The display text you choose will be shown on your child's device and may be displayed to others when your child engages in Toe-to-Toe challenges, depending on your settings, as described below in more detail under *"Personal data that may be collected, processed, and disclosed when your child uses the Toe-to-Toe challenge feature."*

If you choose to invite another parent or guardian in your family to use the Garmin Jr. app, we will collect the name and email address of that person.

Finally, during setup and use, Garmin collects the device identifier (e.g., model name and unit ID) and device settings from each compatible device you activate.

PURPOSES AND LEGAL GROUNDS:

(a) We collect and process your Garmin Connect name and use it as your Admin name, and we collect and process the name you use for your family name in the Garmin Jr. app so your friends and family members can search for you and add you or your family as Connections. This enables you and your family members to participate in challenges with other families if you wish. The legal ground for this processing is our legitimate interest in enabling you and other users to find Connections and engage in challenges.

(b) We collect and process the names you use for your children in your family profile so you can use the Garmin Jr. app's features, such as reviewing and managing each child's activities, chores, and rewards. The legal ground for this processing is your consent. You can withdraw your consent by using text other than your child's name in the name field.

(c) We collect and process the name or other text you input into the display text field so you can choose the name or other text that is shown on your child's device and when your child participates in a Toe-to-Toe challenge, depending on your settings. The legal ground for processing your child's name for this purpose, if you choose to use your child's name in the display text field, is your consent. You can withdraw your consent at any time by changing the display text to some text other than your child's name.

(d) We collect and process your child's photo so you can personalize your child's experience in the Garmin Jr. app. The legal ground for processing your child's photo is your consent. You can withdraw your consent to the processing of your child's photo at any time by removing the photo from your child's profile.

(e) We collect and process your child's birthday so we can improve the Garmin Jr. app and shape future products. The legal ground for processing your child's birthday is your consent. You can withdraw your consent to process your child's

birthday at any time by removing this information from your child's profile.

(f) We collect and process your child's gender so we can improve the Garmin Jr. app and shape future products. The legal ground for processing your child's gender is your consent. You can withdraw your consent to process your child's gender at any time by removing this information from your child's profile.

(g) We collect and process information you give us for a second parent or guardian for the purpose of sending the second parent or guardian an invitation to download the Garmin Jr. app and "join your family." The legal ground for processing this information for these purposes is our legitimate interest in enabling users to add a second parent or guardian to the Garmin Jr. app.

(h) We collect and process information about your child's device (e.g., model name and unit ID) and device settings so that we can associate the device to your profile where you can review and update the device settings. The legal ground for processing this information is our legitimate interest in enabling users to manage device settings.

Personal data that is collected and processed when you upload data from your child's device to Garmin:

When you sync your child's device with the Garmin Jr. app, you will upload to Garmin information about your child's device (e.g., model name and unit ID) and device settings and your child's activity (such as chores, rewards, steps, distance, activity time, and sleep).

PURPOSE AND LEGAL GROUNDS:

Garmin collects and processes your child's activity data to enable you to see and analyze your child's activity data. The legal ground for processing this information is performance of a contract. Information about your child's device and device settings that is uploaded when you sync your child's device with the Garmin Jr. app is collected and processed to help us identify and resolve errors or syncing issues. The legal ground for processing this information for this purpose is Garmin's legitimate interest in providing quality product support.

Personal data that may be collected, processed, and disclosed when your child uses the Toe-to-Toe challenge feature:

The display text you choose to input in the display text field in your Garmin Jr. app, together with the total number of steps taken by your child during the duration of the challenge, will be shown on the devices of your child's friends or family members if your child engages in a Toe-to-Toe challenge with them, and it will be shown in the Garmin Jr. apps of the parents of such friends and family members and in the Garmin Connect account of friends or family members who use other Garmin devices to engage in a Toe-to-Toe challenge with your child. If you do not want your child's actual name to be shown on the devices of other users or the Garmin Jr. apps of their parents or Garmin Connect accounts of others when your child participates in a Toe-to-Toe challenge, then you can use any text other than your child's name that you wish in the display text field.

If you do not wish for the display text to be shown on the devices of other users or the Garmin Jr. apps of their parents or Garmin Connect accounts of others when your child

participates in a Toe-to-Toe challenge even if the display text is not your child's name, then in your Garmin Jr. app, you can change the settings for this to anonymous, in which case the display text shown on the devices of other users or the Garmin Jr. apps of their parents or Garmin Connect accounts of others when your child participates in a Toe-to-Toe challenge will be changed to a generic "JR2" or similar generic and anonymous display text.

PURPOSE AND LEGAL GROUNDS:

If you provide your child's name in the display text field, we collect and process it for purposes of displaying it on your child's device. The legal ground for this processing is your consent, which you can withdraw at any time by changing the display text to something other than your child's name. If you provide your child's name in the display text field, we also collect and process it for purposes of displaying it on the devices and apps of others when your child engages in a Toe-to-Toe challenge. The legal ground for this processing is your consent, which you can withdraw at any time by changing the display text to something other than your child's name or by changing the setting to anonymous.

Personal data that is collected and processed when you use location tracking and communication services:

When you, as the parent, have subscribed to location tracking and communication services (including voice and text messaging or Assistance) for your Family, we collect and process device location and text and voice messages sent through the service. We also process geofences and contact information configured in your Family's account.

PURPOSES AND LEGAL GROUNDS:

(a) We process device location to allow you to see your child's location, determine whether the child has entered or exited a configured geofence, and track your child when a request for help has been received. The legal ground for processing this information for these purposes is performance of a contract.

(b) We process text and voice messages and contact information to facilitate the communications services. We also process your contact information to send you notifications, such as when a request for help has been received. The legal ground for processing this information for these purposes is performance of a contract.

Personal data that is collected and processed when you communicate with Garmin:

When you interact with our customer support representatives by email or telephone, online, or in person, we collect personal data, such as your name, mailing address, phone number, email address, and contact preferences, and information about the Garmin products you own, such as their serial numbers and dates of purchase, and subscriptions you've purchased. We also may create event logs that are useful in diagnosing product or app performance-related issues and capture information relating to the support or service issue. To improve customer service, subject to applicable laws, we may also record and review conversations with customer support representatives and analyze any feedback provided to us through voluntary customer surveys. With your consent, our customer support

representatives may sign in to your Garmin account, if appropriate, to help troubleshoot and resolve your issue.

PURPOSE AND LEGAL GROUNDS:

We use this information to provide you with customer and product support, to monitor the quality and types of customer and product support we provide to our customers, and to facilitate repairs, returns, or exchanges. The legal ground for processing this information for these purposes is Garmin's legitimate interest in providing quality product support. The legal ground for signing in to your Garmin account, if appropriate, to help troubleshoot and resolve your issue is your consent, which you may withdraw.

Categories of Persons to Whom Personal Data Is Disclosed

OTHER GARMIN USERS:

Other Garmin Jr. app users will be able to see your profile. Users who are not your Connections will be able to see the family name you choose in your profile, your admin name, the name of any additional parent or guardian you have added to your profile, and the number of children you have added to your profile. Users who are your Connections will be able to see the same information plus the names you have provided for your children in your profile. You can challenge another family to a steps challenge, and you can set up an intrafamily steps challenge for your family. When you engage in a steps challenge with another family, the total steps taken during the duration of the challenge by each participating member of your family, the names of the participating members of your family, and comments submitted through your Garmin Jr. app by participating members of your family will be shown in the Garmin Jr. app of the other family's admin.

SERVICE PROVIDERS:

We use cloud services from third parties such as Adobe and SendGrid, a Twilio service, to assist in sending emails. Those services track the activities associated with these emails, such as whether they were opened, whether links in the emails were clicked on, and whether purchases were made following clicks on those links. We use this data to analyze the level of engagement with our emails.

We use services from third parties such as Vonage and Twilio to send SMS messages to our users or their contacts in support of features described in this Privacy Policy. We may track

the delivery of these messages based on the phone number of the recipient and the selected content. We use other SMS vendors in some regions, such as Alibaba Cloud (mainland China only), every8d (Taiwan only), aligo sms (South Korea only), fpt / chunghwa (Vietnam only) and true corp (Thailand only).

If you have subscribed to location tracking services for your family, we use a third party, Twilio, to facilitate communication-related features. We also use Google Maps to facilitate location tracking features.

We also use services from third parties such as Alibaba Cloud (mainland China only) to help identify inappropriate or harmful content uploaded by users. We provide personal data to such services only if included in the user's uploaded content.

We use third-party service providers to help us better understand the usage and performance of our products, services, websites, and apps. See the "Monitoring and Analytics" section for more information.

OTHER DISCLOSURES:

We may process and disclose personal data about you and your children to others: (a) if we have your valid consent to do so; (b) to comply with legal and regulatory obligations, such as a valid subpoena, court or judicial order, other valid legal process, or record keeping to support applicable reporting and auditing requirements; (c) to investigate potential fraud and enforce any of our terms and conditions or policies; or (d) as necessary to pursue available legal remedies or defend legal claims.

We may also transfer your and your children's personal data to an affiliate, a subsidiary, or a third party in the event of any reorganization, merger, sale, joint venture, assignment, transfer, or other disposition of all or any portion of our business, assets, or stock, including, without limitation, in connection with any bankruptcy or similar proceeding, provided that any such entity that we transfer personal data to will not be permitted to process your personal data other than as described in this Privacy Policy without providing you notice and, if required by applicable laws, obtaining your consent.

International Transfers of Personal Data

Garmin is a global business. To offer our products, apps, and services, we may need to transfer your personal data to Garmin-owned companies in other countries. View Garmin-owned companies.

When you create a Garmin account, add personal data in your account profile, or upload

data to your Garmin account, your personal data will be collected and stored on servers in the U.S., U.K., and/or Australia.

For customers in mainland China, when you create a Garmin account, add personal data in your account profile, or upload data to your Garmin account, your personal data will be collected and stored on Garmin China Shanghai RHQ Co., Ltd.'s servers in mainland China.

Personal data regarding individuals who reside in a country in the European Economic Area ("EEA"), United Kingdom ("U.K."), or Switzerland is controlled by Garmin Würzburg GmbH and processed on its behalf by other Garmin-owned companies to provide support, infrastructure, security, and other key functions. Garmin Würzburg GmbH has entered into approved model contractual clauses where these companies are not located in the EEA or a jurisdiction deemed adequate under applicable data protection law. Garmin's U.S.-based affiliates are certified under the EU-U.S. and Swiss-U.S. Data Privacy Frameworks. View our Data Privacy Framework Notice.

All Garmin-owned companies are required to follow the privacy practices set forth in this Privacy Policy.

Monitoring and Analytics

We collect data from users about their usage of our products, services, websites, and apps. The types of analytical information that are collected include the date and time of access to our servers, software or firmware version, the location of the device, language setting, what information and files have been downloaded, user behavior (e.g., features used, frequency of use), device state information, device model, hardware and operating system information, and information relating to how the product, service, website, or app functions. Garmin uses this data to improve the quality, security, and functionality of our products, services, websites, or apps; to develop and market products and features that best serve you and other users; and to help identify and fix stability issues and other usability problems as quickly as possible.

The legal ground for processing this analytical information is our legitimate interest in understanding how our customers interact with our products, services, websites, and apps so we can enhance user experience and functionality.

Here are examples of third-party providers we currently use:

- **Firebase (Crashlytics):** Firebase (Crashlytics), a Google service, is used to help us better understand usage to improve user experience and to identify and resolve the root causes of crashes or errors.

Privacy Policy Updates

We may update this Privacy Policy from time to time as we add new products, services, and apps, as we improve our current offerings, and as technologies and laws change. You can determine when this Privacy Policy was last revised by referring to the “Last Updated” legend at the top of this page. Any changes will become effective upon our posting of the revised Privacy Policy.

We will provide notice to you if these changes are material and, where required by applicable law, we will obtain your consent. This notice will be provided by email or by posting notice of the changes on the Garmin websites and apps that link to this Privacy Policy.

Retention of Personal Data

We will retain your personal data as long as your Garmin account is considered to be active or in accordance with applicable law and regulatory obligations. In addition, see below under "Manage Your Family Data" for information on how to delete your family data or account.

Automated Decision-Making, Including Profiling

We do not make any decisions based on algorithms or other automated processing that significantly affect you.

Data Controller and Data Protection Officer

If you reside in a country in the EEA, U.K., or Switzerland, then your personal data collected by Garmin is controlled by Garmin Würzburg GmbH, Beethovenstr. 1a, 97080 Würzburg, Germany. The company's EU Data Protection Officer can be reached by email at euprivacy@garmin.com.

If you reside in mainland China, then your personal data collected by Garmin is controlled by Garmin China Shanghai RHQ Co., Ltd., 37F, Tower A, New CHJ International Business Center, No 391, Guiping Road, Xuhui District, Shanghai 200233, China. The company's Data Protection Officer for mainland China can be reached at the same address and by email at cnprivacy@garmin.com.

If you reside outside of the EEA, U.K., Switzerland, and mainland China, then your personal data collected by Garmin is controlled by Garmin International, Inc., 1200 E. 151st St., Olathe, Kansas 66062, USA, which you can contact by email at privacy@garmin.com. The company's Brazilian Data Protection Officer is located at the same address and can be reached by email at privacy@garmin.com. The company's Turkish Representative can be reached by email at trprivacy@garmin.com.

Manage Your Family Data

Within the Garmin Jr. app, you may access and delete your child's information. Garmin also provides a self-service portal, the Account Management Center, to allow you to access, export, correct, or delete your and your child's data at any time. The Account Management Center requires you to sign in with your Garmin credentials to ensure that only you can manage data and exercise rights on behalf of yourself and your child.

If you want to learn more about your or your child's rights based on where you reside, please visit Your Data Protection Rights. In addition, if you reside in the U.S., as a parent or legal guardian, you have the right under the Children's Online Privacy Protection Act (COPPA) to review or delete the information Garmin collects from your child. You may also withdraw your parental consent or refuse to permit further collection or use of the information. To do so, please contact us by calling us at (913) 397-0872 or by emailing us at privacy@garmin.com.

Further Information

If you have any specific questions, please contact us at:

Garmin Privacy Office
Garmin International, Inc.
1200 E. 151st St.
Olathe, KS 66062-3426

Email: privacy@garmin.com
Phone: (913) 397-8200

All trademarks are the property of their respective owners.

Recent Policy Versions

- Nov. 15, 2022
- June 30, 2022
- April 29, 2021
- June 5, 2020

CUSTOMER SERVICE

COMPANY

PLATFORMS

FOR BUSINESS

United States

Site Map Terms of Use Privacy Security Web Accessibility

© Garmin Ltd. or its subsidiaries. All rights reserved.

Cookie-Präferenzen
