# Supplementary material for: Move-PCD—a multi-center longitudinal randomized controlled superiority trial on the effect of a 6-month individualized supported physical activity (PA) program on quality of life (QoL) in children, adolescents, and adults with primary ciliary dyskinesia
Source: Trials. 2024 Aug 15;25:539. doi: 10.1186/s13063-024-08379-0 (PMC11328395; doi:10.1186/s13063-024-08379-0)
Supplement: Supplementary file 6 — Supplementary Material 6 [file 13063_2024_8379_MOESM6_ESM.pdf]

QOL-PCD

Adults 18 years and older

QUESTIONNAIRE

Your treatment team wants to better understand the impact of PCD and the associated therapies on your life. This knowledge will help to support you with your health and tailor the therapy to you. . Therefore, this questionnaire has been developed specifically for people suffering from primary ciliary dyskinesia (PCD). Thank you for your willingness to complete the questionnaire.

Instructions: The following questions relate to your current state of health as you perceive it. We will then better understand how you feel in your daily life.

Please answer all questions. There are **no** right or wrong answers! If you are not sure, choose the answer that comes closest to your situation.

Notes for doctors and researchers:

This questionnaire may only be used with the consent of the developer consortium. The developers request that any data generated through the use of the questionnaire contribute to the further validation of the instrument. Data related to this questionnaire cannot be published until the results of the developer consortium's validation study are published, which is expected to be in the fall of 2016.

Please contact us for further details and permission:

North America: Prof. Alexandra Quittner: aquittner@miami.edu
Europe and other regions outside North America: Prof. Jane Lucas: jlucas1@soton.ac.uk

 ©2015 Lucas, Leigh, Quittner

QOL-PCD

Adults 18 years and older

Part I. Quality of life

Please choose the answer that fits best.

*During the past **week**, did you have difficulties with the following activities?*	**Major difficultie s**	**Some difficultie s**	**Slight difficultie s**	**No difficultie s**
**1.** Performing strenuous activities (for example gardening or sport)	☐	☐	☐	☐
**2.** To go as fast as others...	☐	☐	☐	☐
**3.** Climbing stairs over more than one floor	☐	☐	☐	☐
**4.** Carrying heavy things such as books or shopping bags	☐	☐	☐	☐

***In general, during the past week**, you give*<br>*Please indicate how often:*	**Always**	**Often**	**Sometimes**	**Never**
**5.** You have felt tired	☐	☐	☐	☐
**6.** You were worried about...	☐	☐	☐	☐
**7.** You felt full of energy...	☐	☐	☐	☐
**8.** You have felt exhausted	☐	☐	☐	☐
**9.** You felt sad...	☐	☐	☐	☐
**10.** You have felt depressed	☐	☐	☐	☐

Please check the box that fits best.

Are you currently undergoing any treatments or therapies (such as taking medication, inhalation, physiotherapy or respiratory therapy)

☐ ***Yes*** ☐ ***No (continue with question 15)***

During the last week,

11. How time-consuming was your daily PCD therapy?

☐ Very complex
☐ Quite complex
☐ Low
☐ Almost no effort

©2015 Lucas, Leigh, Quittner

QOL-PCD

Adults 18 years and older

During the last week, you describe how much
QUESTIONNAIRE

	Not at all	Something	Clear	Very strong
**12.** PCD therapy has made your daily life more difficult.	☐	☐	☐	☐
**13.** you have had difficulties integrating PCD therapy into your Integrate daily routine	☐	☐	☐	☐
**14.** Daily treatment of PCD symptoms (physiotherapy/breathing exercises) You are tired	☐	☐	☐	☐

*Think back to your state of health **last week**. Describe how accurately the following statements apply to you.*

	Hits exactly true	Rather true	Rather not applicabl e	Does not apply at all
**15.** It was okay for me to cough in front of others..........................	☐	☐	☐	☐
**16.** I felt uncomfortable in the presence of sick people	☐	☐	☐	☐
**17.** I felt lonely..	☐	☐	☐	☐
**18.** It was okay for me to blow my nose in front of others.......	☐	☐	☐	☐
**19.** It was okay for me to do my therapy in the presence of others. (physiotherapy, respiratory therapy)	☐	☐	☐	☐

Part II School, work or daily activities

During the past week:

20. How difficult was it for you to manage your work, household or other daily activities?

☐ They had no difficulty coping with them
☐ They managed to overcome them, but it was difficult
☐ They only managed it in part
☐ You didn't manage to complete your tasks

21. How much has PCD prevented you from achieving your goals at work, at home, with your family or in your personal life?

☐ Very strong ☐ Clearly ☐ A little bit ☐ Not at all

©2015 Lucas, Leigh, Quittner

QOL-PCD

Adults 18 years and older

QUESTIONNAIRE

*Think back to your state of health last **week** and describe how accurately the following statements apply to you*

	Exactly right	Largely true	True a little	Not true at all
**22.** It was difficult to make plans for the future (e.g. job change, family planning, vacation planning, organization of everyday life).	☐	☐	☐	☐
**23.** I led a normal life..	☐	☐	☐	☐
**24.** I was worried that my health could deteriorate in the future...	☐	☐	☐	☐
**25.** I felt healthy	☐	☐	☐	☐
**26.** I managed to carry out my daily therapies for the PCD....	☐	☐	☐	☐

Please check the box that fits best.

If you look back on the past week:

27. How would you rate your state of health?

☐ xcellent

☐ Good

☐ ufficient

☐ Bad

Part III Symptoms

Please choose the answer that fits best.

*Describe how you felt last **week**:*

	Very strong	Clear	Something	Not at all
**28.** You had mucus in your lungs	☐	☐	☐	☐
**29.** You heard poorly (if you wear a hearing aid: you had difficulty hearing without a hearing aid)	☐	☐	☐	☐
**30.** You felt mucus running from your nose to the back of your throat	☐	☐	☐	☐
**31.** You had pain in the area of the frontal sinuses or maxillary sinuses......	☐	☐	☐	☐
**32.** You had a feeling of tightness in your chest...	☐	☐	☐	☐
**33.** They had to cough up phlegm...	☐	☐	☐	☐ **If "even not", further to question 35**

©2015 Lucas, Leigh, Quittner

QOL-PCD

Adults 18 years and older

During the last week:

QUESTIONNAIRE

34. was your mucus mainly:

- ☐ Clear
- ☐ Clear to yellowish
- Yellowish-greenish
- ☐ Greenish
- ☐ Greenish with traces of blood
- ☐ I don't know

During the past ***week,*** *please indicate how often:*

	Always	Often	Sometimes	Never
**35.** you were short of breath during strenuous exertion (for example when doing housework or gardening)	☐	☐	☐	☐
**36.** you had whistling or wheezing breathing	☐	☐	☐	☐
**37.** you had a runny nose..	☐	☐	☐	☐

Indicate how often in the last ***week:***

	Always	Often	Sometimes	Never
**38.** you were short of breath when speaking	☐	☐	☐	☐
**39.** you had a feeling of pressure in your ears	☐	☐	☐	☐
**40.** your nose was blocked	☐	☐	☐	☐

Please check that you have answered all the questions.

Thank you very much!

©2015 Lucas, Leigh, Quittner

QOL-PCD

Teenagers (13 to 17 years)

QUESTIONNAIRE

Your treatment team wants to better understand the impact of PCD and the associated therapies on your life. This knowledge will help us to further improve treatment. That's why this questionnaire has been developed specifically for people who suffer from primary ciliary dyskinesia (PCD). Thank you very much for your willingness to complete it.

Instructions: The following questions relate to your current state of health as you perceive it. We will then better understand how you feel in your daily life.
Please answer all questions. There are **no** right or wrong answers! If you are not sure, choose the answer that comes closest to your situation.

For further details and permission please contact us:
North America: Prof. Alexandra Quittner: aquittner@miami.edu
Europe and other regions outside North America: Prof. Jane Lucas: jlucas1@soton.ac.uk

 ©2015, Dell, Lucas, Leigh, Quittner

QOL-PCD

Teenagers (13 to 17 years)

Part I. Quality of life

Please check the appropriate box.

In the last ***week****, you had difficulties with the following activities:*

	Major difficulties	Some difficulties	Slight difficulties	No difficulties
**1.** Performing strenuous activities (for example running or sports)	☐	☐	☐	☐
**2.** To go as fast as others	☐	☐	☐	☐
**3.** Climbing stairs as fast as others...	☐	☐	☐	☐

In general, during the past ***week,*** *please indicate how often:*

	Always	Often	Sometimes	Never
**4.** You have been well.	☐	☐	☐	☐
**5.** You were worried about getting sick...	☐	☐	☐	☐
**6.** You have been happy	☐	☐	☐	☐
**7.** You have been tired..	☐	☐	☐	☐
**8.** You felt adventurous	☐	☐	☐	☐
**9.** You have been exhausted	☐	☐	☐	☐
**10.** You have been sad..	☐	☐	☐	☐
**11.** Your PCD therapies have prevented you from carrying out your activities	☐	☐	☐	☐
**12.** The implementation of your therapies has frustrated you	☐	☐	☐	☐

Please mark the answer that fits best. Please choose only one answer for each question.

Remember your state of health in the last ***week****:*

13. How difficult was it for you to integrate your therapies into your daily routine (including taking your medication)?

☐ Not difficult at all

☐ Somewhat difficult

☐ Quite difficult

☐ Very difficult

©2015, Dell, Lucas, Leigh, Quittner

QOL-PCD

Teenagers (13 to 17 years)

QUESTIONNAIRE

Please check the appropriate box.
Remember your state of health in the last ***week.***
Describe how much each sentence is right or wrong for you.

	**Fits exactly**	**Rather true**	**Rather not applicabl e**	**Does not apply at all**
**14.** I had trouble recovering after physical exertion				
**15.** I had to restrict activities such as running or sport	☐	☐	☐	☐
**16.** I was able to talk well with others about my illness	☐	☐	☐	☐
**17.** It was okay for me to blow my nose in front of my friends...............	☐	☐	☐	☐
**18.** The other people were afraid that I would infect them	☐	☐	☐	☐
**19.** I had the feeling that my coughing was disturbing other people....................	☐	☐	☐	☐
**20.** It was difficult to make plans for the future (for example, making decisions about secondary school, vocational training or a job, etc.).	☐	☐	☐	☐
	☐	☐	☐	☐

Part III School, work and daily activities

21. How often have you been absent from school or work or been unable to complete daily activities in the last week due to your illness or therapy?

☐ Always ☐ Often Sometimes ☐ Never

22. To what extent has PCD prevented you from achieving your own goals for school, work or elsewhere?

☐ Very strong ☐ Clearly ☐ A little bit ☐ Not at all

©2015, Dell, Lucas, Leigh, Quittner

QOL-PCD

Teenagers (13 to 17 years)

Part IV Symptoms

Please check the appropriate box.

*Describe how you felt last **week**:*	Very strong	Clear	Something	Not at all
**23.** Did your lungs feel mucousy?	☐	☐	☐	☐
**24.** Did you cough during the day?	☐	☐	☐	☐
**25.** Did you have to cough up mucus (even if you then swallowed it)?	☐	☐	☐	☐ **If "not at all", continue to Question 27**

26. Was your slime mainly:

☐ clear ☐ clear to yellowish ☐ yellowish-greenish ☐ greenish ☐ greenish with some blood ☐ I don't know

	Very much	Clear	Something	Not at all
**27.** Fluid has come out of your ears..	☐	☐	☐	☐
**28.** You had difficulty hearing (if you have a hearing aid: did you have difficulty hearing without your hearing aid?).......................	☐	☐	☐	☐
**29.** You have noticed that mucus runs down the back of your throat.............	☐	☐	☐	☐

*In the last **week**, indicate how often:*	Always	Often	Sometimes	Never
**30.** your breathing has made a noise (whistling, wheezing, rattling)	☐	☐	☐	☐
**31.** you have had a blocked nose	☐	☐	☐	☐
**32.** you slept badly because of breathing problems	☐	☐	☐	☐
**33.** you had trouble breathing	☐	☐	☐	☐
**34.** you have had an earache..	☐	☐	☐	☐
**35.** you have had a runny nose...	☐	☐	☐	☐
**36.** you didn't sleep well because your nose was blocked........	☐	☐	☐	☐
**37.** your ears have been blocked	☐	☐	☐	☐
**38.** you have had a headache...	☐	☐	☐	☐

Please check again that you have answered all the questions.

Thank you very much

©2015, Dell, Lucas, Leigh, Quittner

QOL-PCD QUESTIONNAIRE Children 6-12 years

These questions are for children like you who have PCD. Your answers will help us better understand this disease and learn more about what treatments can help you. Your answers will help you and other children with PCD feel better in the future.

Please answer all questions. There are no right or wrong answers! If you are not sure how to answer, choose the answer that suits you best.

Important information for physicians and study assistants:
Use of the questionnaire without the consent of the publishers is not permitted. For the evaluation of the questionnaire, the publishers request all data generated with the help of this questionnaire. Data collected with the help of this questionnaire can only be published after the evaluation of this questionnaire has been completed and the results have been published by the publisher.

For further information, please contact:
- North America: Prof. Quittner; aquittner@miami.edu
- Europe and regions outside North America: Prof. Jane Lucas; jlucas1@soton.ac.uk

QOL-PCD

Children 6-12 years

QUESTIONNAIRE

Please check the box that is correct for you.

During the last **week**:	Exactly right	Quite right	A little bit true	Not true at all
**1.** You could walk just as fast as other children..................	☐	☐	☐	☐
**2.** You could climb stairs just as quickly as other children...	☐	☐	☐	☐
**3.** You could run, jump and climb as much as you wanted	☐	☐	☐	☐
**4.** You could run as fast and as long as other children...	☐	☐	☐	☐
**5.** You could do all the sports that you enjoy (e.g. Soccer, dancing or others)	☐	☐	☐	☐

Please check the box that is correct for you.

During the last **week**, how often:	Immer	Oft	Sometimes	Nie
**6.** Were you angry	☐	☐	☐	☐
**7.** Were you in a bad mood	☐	☐	☐	☐
**8.** Were you afraid of getting sick	☐	☐	☐	☐
**9.** Were you sad	☐	☐	☐	☐
**10.** Were you annoyed to do your daily exercises (inhalation, nasal rinsing, coughing, gymnastics)..............................	☐	☐	☐	☐
**11.** Did you have to interrupt a game or something else to do your therapies (inhalation, nasal rinsing, coughing, gymnastics)? make...	☐	☐	☐	☐

QOL-PCD

Children 6-12 years

QUESTIONNAIRE

Please check the box that is correct for you.

During the last **week:**	**Exactly right**	**Quite right**	**A little bit true**	**Not true at all**
**12.** You were late for school or absent from school because of the PCD	☐	☐	☐	☐
**13.** You had enough time to do all your therapies (inhalation, nasal rinsing, coughing, gymnastics)	☐	☐	☐	☐
**14.** You found it uncomfortable to do your therapies (inhalation, nasal rinsing, coughing, gymnastics) in front of your friends....	☐	☐	☐	☐
**15.** You spent a lot of time with your friends..........................	☐	☐	☐	☐
**16.** You couldn't play with friends after school because of the PCD	☐	☐	☐	☐
**17.** You have been laughed at by other children because of your runny nose..	☐	☐	☐	☐
**18.** Other people were afraid you would infect them.........	☐	☐	☐	☐
**19.** You had difficulty hearing (if you have a hearing aid: you had difficulty hearing without your hearing aid)	☐	☐	☐	☐
**20.** It bothered you to do your therapy (inhalation, nasal rinsing, coughing, gymnastics)	☐	☐	☐	☐
**21.** Your ears were blocked	☐	☐	☐	☐

QOL-PCD

Children 6-12 years

QUESTIONNAIRE

Please check the box that is correct for you.

Please tell us how often in the last **week**:

	Always	Often	Sometimes	Never
**22.** You coughed during the day	☐	☐	☐	☐
**23.** You had an earache	☐	☐	☐	☐
**24.** You woke up in the night because you were coughing	☐	☐	☐	☐
**25.** You coughed up mucus. Maybe you also swallowed it.	☐	☐	☐	☐

Please tell us how many times in the last **week**:

	Always	Often	Sometimes	Never
**26.** You had trouble breathing..	☐	☐	☐	☐
**27.** Fluid came out of your ears	☐	☐	☐	☐
**28.** You woke up at night because your nose was blocked	☐	☐	☐	☐
**29.** Your chest hurt...	☐	☐	☐	☐
**30.** You had mucus in your lungs...	☐	☐	☐	☐
**31.** You had a runny nose...	☐	☐	☐	☐
**32.** You had a headache (in the forehead or behind the eyes)	☐	☐	☐	☐

33. your nose was blocked.. ☐ ☐ ☐ ☐

34. you had the feeling that mucus was running down the back of your throat....... ☐ ☐ ☐ ☐

Please check again that you have answered all the questions.

Thank you very much

Your treatment team wants to better understand how your child's illness and treatment affect their daily life. This knowledge helps us to further improve care. For this reason, a special quality of life questionnaire has been developed for parents of children with PCD. Thank you for your willingness to complete this questionnaire.

Instructions: The following questions relate to your child's current state of health as the child perceives it. This information will enable us to understand better, how it feels in daily life. Please answer all questions. There are no right or wrong answers! If you are not sure how to answer, choose the answer that comes closest to your child's situation.

Important information for physicians and study assistants:
Use of the questionnaire without the consent of the publishers is not permitted. For the evaluation of the questionnaire, the publishers request all data generated with the help of this questionnaire. Data collected with the help of this questionnaire can only be published after the evaluation of this questionnaire has been completed and the results have been published by the publisher.

For further information, please contact:

- North America: Prof. Quittner; aquittner@miami.edu
- Europe and regions outside North America: Prof. Jane Lucas; jlucas1@soton.ac.uk

 ©2015 Dell, Lucas, Leigh & Quittner

Part I. Quality of life

Please describe how your child felt last week. Please check all that apply.

Did your child have difficulties with the following activities:	Major difficulties	Some difficulties	Slight difficulties	No difficulties
**1.** Performing strenuous activities such as running or playing sports	☐	☐	☐	☐
**2.** Walking as fast as other children...	☐	☐	☐	☐
**3.** Climbing stairs as fast as other children	☐	☐	☐	☐
**4.** Climbing stairs over several floors	☐	☐	☐	☐

Please check the appropriate box.

In general, please indicate how often your child in the past week:	Always	Often	Sometimes	Never
**5.** .... seemed happy	☐	☐	☐	☐
**6.** Concerned about his illness, ...	☐	☐	☐	☐
**7.** Tired appeared	☐	☐	☐	☐
**8.** Looking healthy and vital	☐	☐	☐	☐
**9.** The enterprising	☐	☐	☐	☐
**10** Arrived late to school, was absent from school or missed other activities due to illness or therapy.	☐	☐	☐	☐
**11.** He was frustrated when carrying out his therapy..	☐	☐	☐	☐

©2015 Dell, Lucas, Leigh & Quittner

Please check the appropriate box.

Recall your child's state of health in the last ***week****. Please describe to what extent each sentence applies or does not apply to your child:*

	Exactly right	Rather true	Rather not true	True not at all
**12.** My child had difficulty recovering after physical exertion	☐	☐	☐	☐
**13.** The meals were laborious	☐	☐	☐	☐
**14.** The therapy (inhalation, physiotherapy) hindered my child in his daily activities	☐	☐	☐	☐
**15.** My child felt healthy overall..	☐	☐	☐	☐
**16.** My child got enough help at school to perform well (e.g. sits at the front, gets time to catch up on homework when sick).......	☐	☐	☐	☐
**17.** My child was able to do their homework without any problems and also take part in outdoor activities...	☐	☐	☐	☐
**18.** My child spent a lot of time every day on his therapies (with the medication and physiotherapy exercises)...	☐	☐	☐	☐

Please circle the answer that best suits your child. Please select only one answer per question.

19. How difficult is it for your child to integrate the therapy into his or her daily routine (including taking medication)?

☐ Not difficult at all

☐ Rather difficult

☐ Quite difficult

☐ Very difficult

20. How would you rate your child's current state of health?

☐ Very good

☐ Good

☐ Satisfactory

☐ Bad

 ©2015 Dell, Lucas, Leigh & Quittner

Part II Symptoms

The next questions are designed to find out how often your child has symptoms such as coughing or shortness of breath.

Please indicate how your child has felt in the last week.	**Very strong**	**Clear**	**Something**	**Not at all**
**21.** My child had problems gaining weight	☐	☐	☐	☐
**22.** My child had an earache	☐	☐	☐	☐
**23.** My child's lungs were full of mucus	☐	☐	☐	☐
**24.** My child coughed during the day	☐	☐	☐	☐
**25.** My child had to cough up phlegm. Maybe he also swallowed it.	☐	☐	☐	☐
**26.** My child had a runny nose	☐	☐	☐	☐
**27.** My child had the feeling that mucus was running from the nose to the back of the throat	☐	☐	☐	☐

*During the past **week**:*	**Always**	**Often**	**Sometimes**	**Never**
**28.** My child had difficulties hearing (if they have a hearing aid: did they have difficulties without a hearing aid?)...	☐	☐	☐	☐
**29.** My child was leaking fluid from his ears...	☐	☐	☐	☐
**30.** My child had a headache in the sinus or maxillary sinus area.	☐	☐	☐	☐
**31.** My child had trouble breathing...	☐	☐	☐	☐
**32.** My child woke up at night due to coughing	☐	☐	☐	☐
**33.** My child had a blocked nose	☐	☐	☐	☐
**34.** My child had chest pain	☐	☐	☐	☐
**35.** My child had little appetite	☐	☐	☐	☐

Please check that you have answered all the questions

Thank you very much!

Hier bitte das Logo des eigenen Zentrums einfügen

Sports activities data sheet

Study-ID:________________________ **Date:**__________________

1. Are you regularly active in sports? ☐ yes ☐ no

If so, continue with question 2

If not, continue with question 6

2. Who do you usually do sport with?

☐ alone ☐ in pairs ☐ with several friends

3. How often are you active per week:

☐ 1x ☐ 2x ☐ 3x ☐ > 3x

4. How long does a unit last:

☐ < 20 min ☐ 20 to 40 min ☐ 40 to 60 min ☐ > 60 min

5. What sport do you do regularly:

(Multiple answers possible)

☐ Running ☐ Biking ☐ Swimming ☐ Strength training
☐ ball game ☐ Badminton/ Tennis ☐ Table tennis

6. How strongly do the following influencing factors keep you from exercising?

Weather	☐ not at all	☐ a little	☐ strongly	☐ very strongly
Fatigue	☐ not at all	☐ a little	☐ strongly	☐ very strongly
Shortness of breath	☐ not at all	☐ a little	☐ strongly	☐ very strongly
Lack of desire	☐ not at all	☐ a little	☐ strongly	☐ very strongly
Pain	☐ not at all	☐ a little	☐ strongly	☐ very strongly
Fear of damage to health	☐ not at all	☐ a little	☐ strongly	☐ very strongly
Lack of physical fitness	☐ not at all	☐ a little	☐ strongly	☐ very strongly

GEFÖRDERT VOM
Bundesministerium für Bildung und Forschung

Hier bitte das Logo des eigenen Zentrums einfügen

Training request form

1. Which form of training do you prefer?

(Multiple answers possible)

☐ Endurance training
☐ Muscle building training
☐ Coordination training
☐ Flexibility training

2. How long should an average training session last for you?

☐ 20 min ☐ 40 min ☐ 60 min ☐ > 60 min

Do you have a specific training goal? ☐ yes ☐ no

If so, which? ____

____

____

Hier bitte das Logo des Eigenen Zentrums einfügen

Move-PCD

A multicenter randomized controlled longitudinal study of the impact of a six-month individualized and supervised activity program on quality of life in children, adolescents and adults with primary ciliary dyskinesia (PCD)

Checklist for trainers
Weekly telephone appointments in the intervention group

1. **Date / start of the intervention**

Interview has taken place		yes□	no □
If not:	Time prevented □	sick □	unknown □
The individual training plan was discussed:		yes□	no □
Safety questionnaire* answered:		yes□	no □
→ Proband can start with intervention?		yes□	no □

2. **Follow-up appointments** (must be documented individually)

Interview has taken place		yes□	no □
If no:	Time prevented □	sick □	unknown □
The training plan has been adjusted		yes□	no □
If so, why:	too exhausting □	too time-consuming □	is no fun □
Safety questionnaire* answered		yes□	no □
→ Proband informed about necessary intervention break		yes□	no □

GEFÖRDERT VOM
Bundesministerium für Bildung und Forschung

Hier bitte das Logo des Eigenen Zentrums einfügen

* Safety questionnaire

There are 6 standardized, neutrally formulated questions, which are graded from 0 = very poor to 10 = very good. The probands are given an analog-numerical scale to take home with them during the screening visit (see illustration). If the minimum value is < 3, the immediate information to the lead study center in Bochum will be send by e-mail via the central database. The trainer then will instructed the proband to pause the intervention, until their doctor will inform them to participate again.

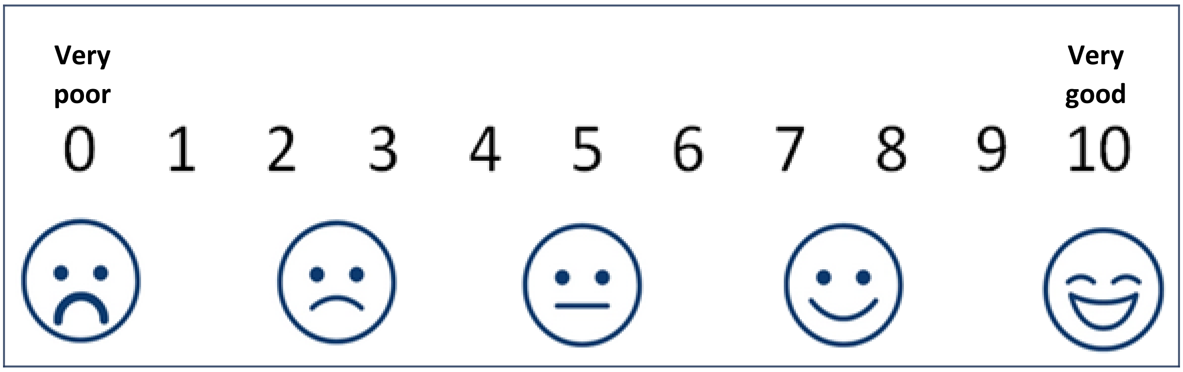

To introduce the questions:
"All questions relate to the past seven days. Please tell me how good your best day was and how bad your worst day was. It can also be the same number."

1. How healthy do you feel?
2. How do you cope with your breathing during training?
3. How well do you cope with your breathing in everyday life?
4. How can you cope with your everyday life?
5. How can you manage your training?
6. How is your sleep?

GEFÖRDERT VOM Bundesministerium für Bildung und Forschung
