# Supplementary material for: Move-PCD—a multi-center longitudinal randomized controlled superiority trial on the effect of a 6-month individualized supported physical activity (PA) program on quality of life (QoL) in children, adolescents, and adults with primary ciliary dyskinesia
Source: Trials. 2024 Aug 15;25:539. doi: 10.1186/s13063-024-08379-0 (PMC11328395; doi:10.1186/s13063-024-08379-0)
Supplement: Supplementary file 8 — Supplementary Material 8 [file 13063_2024_8379_MOESM8_ESM.pdf]

Catholic HospitalBochumocnum

St. Josef Hospital
RUB UNIVERSITÄTSKLINIKUM DER RUHR-UNIVERSITÄT BOCHUM

Univers ltam unit er fiinaer- end oveenö m-dixn - z Pz - Ai--andrine nsid s - ia79j goch u p

Ärztekammer Westfalen-Lippe
Körperschaft des öffentlichen Rechts Ethics Committee
Gartenstrasse 210 - 214
48147 Münster

Department of Pediatrics and Adolescent Medicine Director
Prof. Dr. med. Thomas Lücke

Sandra Kühn (Office)
Phone 0234/509-261 1
Fax 0234/509-2612
sandra.kuehn@klinikum-bochum.de www.kinderklinik-bochum.de

14.06.2024

Subject

Amendment No. 23-7938 (formerly Bochum) **/ No. 2023-64_1** (UK Münster)

Research project: Move-PCD
A multicenter, randomized, controlled, longitudinal study of the impact of a **six-month individualized and supervised activity program on quality of life in** children, **adolescents and** adults **with primary** ciliary dyskinesia (PCD)

Dear Ethics Committee,

\- Enclosed you will find the revised versions as part of an application for amendments to the project **"Move-PCD - A multicenter, randomized, controlled longitudinal study of the impact of** of a **six-month** individualized and supervised **activity program** on the **quality of life in** Children, adolescents **and adults with primary ciliary dyskinesia** (PCD)".

The research project is a multicenter, randomized and controlled study funded by the DLR on behalf of the BMBF within the framework of patient-oriented research. The aim is to include 159 children, adolescents and adults with primary ciliary dyskinesia at the leading study center in Bochum and at 6 other locations in Germany as well as in cooperation with the "Kartagener Syndrom und Primäre Ciliäre Dyskinesie e.V".

Initially, we excluded subjects with specific genetic backgrounds (CCDC39/40, CCNO) due to their predisposition to rapid disease progression with faster loss of lung function. Now that we have received many inquiries from people with PCD with precisely these genetic mutations and interest in participating, we have contacted the DLR. We would now also like to include these patients in the study and evaluate them as a subgroup. All other exclusion criteria remain unaffected. The DLR is in agreement with the procedure. We would like to

now also have it assessed by you as an amendment. We and the patients would be very pleased to receive positive feedback.

In addition, we once again compared the exclusion criteria of the subject information and the study protocol and supplemented them in the appropriate places.

We submit all documents in which we have made changes as general ethics documents for the entire study (clean) as well as the versions for Bochum as a study center (Bochum).

In addition, after consultation with Ms. Raidt (UKM), you will also find attached the amended documents for the Münster study center for joint review.
All changes are marked in green.

If you require further documents, please do not hesitate to contact us. The

following documents are enclosed with the application:

- Move PCD Protocol final signed V1.2 (study protocol, English version)
- Subject information adults V1.2 (clean, Bochum, Münster)
- Subject information for parents and guardians V 1.2(clean, Bochum, Münster)
- Visitsheet inclusion/exclusion criteria V 1.1 (clean, Bochum, Münster)
- Ethics vote no. 23-7938 (Leitethik/Bochum)
- Ethics vote no. 2023-64 1 (Münster)

If you have any questions, please do not hesitate to

contact us by telephone. Yours sincerely

ProDr. med. [illegible] Lake [illegible]
Project management
(Bochum)

Prof. Dr. med. Folke [illegible]
Brinkmann Project
management
