# Supplementary material for: Move-PCD—a multi-center longitudinal randomized controlled superiority trial on the effect of a 6-month individualized supported physical activity (PA) program on quality of life (QoL) in children, adolescents, and adults with primary ciliary dyskinesia
Source: Trials. 2024 Aug 15;25:539. doi: 10.1186/s13063-024-08379-0 (PMC11328395; doi:10.1186/s13063-024-08379-0)
Supplement: Supplementary file 7 — Supplementary Material 7. [file 13063_2024_8379_MOESM7_ESM.pdf]

Patientenschutz | Forschungsfreiheit

ÄRZTEKAMMER WESTFALEN-LIPPE · Universität Münster · UNIVERSITÄT BIELEFELD Medizinische Fakultät OWL

ETHIK KOMMISSION Westfalen-Lippe

Gartenstraße 210 – 214
48147 Münster, Germany
Tel.: +49 (0)251 929 2460
Fax: +49 (0)251 929 2478
E-Mail: ethik-kommission@aekwl.de
www.ethik-kommission-wl.de

Ethik-Kommission Westfalen-Lippe · Gartenstraße 210 – 214 · 48147 Münster

Mr.
Prof. Dr. med. Folke Brinkmann
St. Josef-Hospital
University Clinic for Pediatrics and Adolescent
Medicine Alexandrinenstraße 5
44791 Bochum

by e-mail only:
anna.hoffmann-n28@ruhr-uni-bochum.de

July 1, 2024 CS

**File number** **(please always include):**	**2024-448-f-S**
Title of the research project:	Move-PCD<br>A multicenter randomized controlled longitudinal study of the impact of a six-month individualized and supervised activity program on quality of life in children, adolescents and adults with primary ciliary dyskinesia (PCD)
Here:	03_subst. Amendment, application dated 14.06.2024

Dear Professor Brinkmann,

You have requested the Ethics Committee's advice on subsequent amendments to the above-mentioned research project. The Ethics Committee has discussed and decided on your application in a committee:

The Ethics Committee still has no fundamental ethical or legal objections to the implementation of the research project.

This assessment applies to the research project as it appears on the basis of the documents listed in Annex 1.

The Medical Association of Westphalia-Lippe charges fees for the decision of the Ethics Committee in accordance with its administrative fee schedule. A separate notification of the fees will be issued.

General information:

With this statement, the Ethics Committee advises the physicians belonging to the Westphalia-Lippe Medical Association on the professional ethics and professional law issues associated with the research project in accordance with Section 15 (1) of the ÄKWL Professional Code.
If you have submitted the application on behalf of other physicians involved, you are obliged to inform all participating physicians of the results of this consultation.

The assessment of the Ethics Committee is not binding for the applicant as an open-ended consultation. Irrespective of this opinion, the medical, ethical and legal responsibility for the implementation of the research project remains with the head of the project, the client and all doctors involved in the project.

The Westfalen-Lippe Ethics Committee is organized and works in accordance with the statutory provisions and the GCP guidelines of the ICH.

Yours sincerely

Prof. Dr. med. Wolfgang E. Berdel Chairman of the
Ethics Committee

Appendix 1 The

following documents were submitted to the Ethics Committee:
If there are several versions of a document, the rating refers to the latest version:

**Entrance**	**Dating**	**Attachment**
01.07.2024	14.06.2024	7938-Vote
01.07.2024	14.06.2024	Cover letter to the ethics committee MOVE PCD_Amendement_signed
01.07.2024		14.06.2024 Parent education Move PCD_V1.2_Bochum
01.07.2024		14.06.2024 Clarification for parents Move PCD_V1.2_clean
01.07.2024	14.06.2024	Parent education Move PCD_V1.2_UKM
01.07.2024	14.06.2024	Adult education Move PCD_V1.2_Bochum
01.07.2024		14.06.2024 Enlightenment Adults Move PCD_V1.2_clean
01.07.2024	14.06.2024	Education Adults Move PCD_V1.2_UKM
01.07.2024	14.06.2024	BO_AS_2023_12_12_2023-64_1-other research first
01.07.2024		14.06.2024 Inclusion and exclusion Bochum_V1.1
01.07.2024	14.06.2024	Inclusion and exclusion Clean_V1.1
01.07.2024	14.06.2024	Inclusion and exclusion Münster_V1.1
01.07.2024	14.06.2024	Move_PCD Protocol_final_signed_V1.2
01.07.2024 2023-64_1		14.06.2024 WG_ Amendment No_ 23-7938 (formerly Bochum) _ No_ (UK Münster)
